# Surviving in the Brine: A Multi-Omics Approach for Understanding the Physiology of the Halophile Fungus *Aspergillus sydowii* at Saturated NaCl Concentration

**DOI:** 10.3389/fmicb.2022.840408

**Published:** 2022-05-02

**Authors:** Irina Jiménez-Gómez, Gisell Valdés-Muñoz, Aldo Moreno-Ulloa, Yordanis Pérez-Llano, Tonatiuh Moreno-Perlín, Hortencia Silva-Jiménez, Fernando Barreto-Curiel, María del Rayo Sánchez-Carbente, Jorge Luis Folch-Mallol, Nina Gunde-Cimerman, Asunción Lago-Lestón, Ramón Alberto Batista-García

**Affiliations:** ^1^Centro de Investigación en Dinámica Celular, Instituto de Investigación en Ciencias Básicas y Aplicadas, Universidad Autónoma del Estado de Morelos, Cuernavaca, Mexico; ^2^Departamento de Innovación Biomédica, Centro de Investigación Científica y de Educación Superior de Ensenada, Ensenada, Mexico; ^3^Centro de Ciencias Genómicas, Universidad Nacional Autónoma de México, Cuernavaca, Mexico; ^4^Instituto de Investigaciones Oceanológicas, Universidad Autónoma de Baja California, Ensenada, Mexico; ^5^Facultad de Ciencias Marinas, Universidad Autónoma de Baja California, Ensenada, Mexico; ^6^Centro de Investigación en Biotecnología, Universidad Autónoma del Estado de Morelos, Cuernavaca, Mexico; ^7^Department of Biology, Biotechnical Faculty, University of Ljubljana, Ljubljana, Slovenia

**Keywords:** halophilic fungus, extremophilic fungi, transcriptomics, metabolomics, saturated NaCl solution, water deprivation, low water activity, salt stress

## Abstract

Although various studies have investigated osmoadaptations of halophilic fungi to saline conditions, only few analyzed the fungal mechanisms occurring at saturated NaCl concentrations. Halophilic *Aspergillus sydowii* is a model organism for the study of molecular adaptations of filamentous fungi to hyperosmolarity. For the first time a multi-omics approach (i.e., transcriptomics and metabolomics) was used to compare *A. sydowii* at saturated concentration (5.13 M NaCl) to optimal salinity (1 M NaCl). Analysis revealed 1,842 genes differentially expressed of which 704 were overexpressed. Most differentially expressed genes were involved in metabolism and signal transduction. A gene ontology multi-scale network showed that ATP binding constituted the main network node with direct interactions to phosphorelay signal transduction, polysaccharide metabolism, and transferase activity. Free amino acids significantly decreased and amino acid metabolism was reprogrammed at 5.13 M NaCl. mRNA transcriptional analysis revealed upregulation of genes involved in methionine and cysteine biosynthesis at extreme water deprivation by NaCl. No modifications of membrane fatty acid composition occurred. Upregulated genes were involved in high-osmolarity glycerol signal transduction pathways, biosynthesis of β-1,3-glucans, and cross-membrane ion transporters. Downregulated genes were related to the synthesis of chitin, mannose, cell wall proteins, starvation, pheromone synthesis, and cell cycle. Non-coding RNAs represented the 20% of the total transcripts with 7% classified as long non-coding RNAs (lncRNAs). The 42% and 69% of the total lncRNAs and RNAs encoding transcription factors, respectively, were differentially expressed. A network analysis showed that differentially expressed lncRNAs and RNAs coding transcriptional factors were mainly related to the regulation of metabolic processes, protein phosphorylation, protein kinase activity, and plasma membrane composition. Metabolomic analyses revealed more complex and unknown metabolites at saturated NaCl concentration than at optimal salinity. This study is the first attempt to unravel the molecular ecology of an ascomycetous fungus at extreme water deprivation by NaCl (5.13 M). This work also represents a pioneer study to investigate the importance of lncRNAs and transcriptional factors in the transcriptomic response to high NaCl stress in halophilic fungi.

## Introduction

*Aspergillus sydowii* is an ubiquitous, halophilic, and saprotrophic fungus found in both terrestrial and marine environments (Geiser et al., 1998). It has been described as a human opportunistic pathogen (de Hoog et al., 2020), while it is also considered an important pathogen of coral reefs (Geiser et al., 1998; Ein-Gil et al., 2009). Interestingly, only marine isolates of the fungus can cause the disease of sea-fan corals (*Gorgonia ventalina*; Geiser et al., 1998), exposing the potential of hypersaline marine environments (e.g., salterns) as natural reservoirs for dissemination of the fungus (Chung et al., 2019). Although *A. sydowii* does not have an obligate requirement for NaCl in the media (e.g., Potato Dextrose Agar, Malt Extract Agar, Czapek Dox Agar, Sabouraud Dextrose Agar), it grows optimally at 1 M NaCl and even (albeit weakly) at saturated NaCl (5.13 M; Jiménez-Gómez et al., 2020). Due to its broad tolerance of salinity range, from no NaCl to 5.13 M NaCl, *A. sydowii* is a model to study adaptations to extremely saline conditions in filamentous fungi (Jiménez-Gómez et al., 2020; Pérez-Llano et al., 2020; Rodríguez-Pupo et al., 2021). This fungus grows primarily in hyphal form unlike other extreme halophiles that grow meristematically such as the basidiomycetous black yeast *Hortaea werneckii* (Gunde-Cimerman et al., 2000), the ascomycetous black yeast *Aureobasidium pullulans* (Zalar et al., 2008), or the basidiomycetous filamentous fungus *Wallemia ichthyophaga* (Zajc et al., 2013). At both zero and optimal (1 M) NaCl concentrations, vegetative hyphae display polarized growth, colonies are large with pigmented reverse, and abundant production of conidia. At saturated NaCl (5.13 M), colonies become smaller, flatter, hyaline, and do not produce either conidia or exudates. In addition, the average length of hyphal compartments decreases, while hyphal ramification increases (Jiménez-Gómez et al., 2020). At optimal (1 M) and saturated NaCl concentrations (5.13 M) *A. sydowii* EXF-12860 produces intracellularly a range of compatible solutes, such as trehalose, mannitol, arabitol, erythritol, and glycerol (Jiménez-Gómez et al., 2020; Pérez-Llano et al., 2020; Rodríguez-Pupo et al., 2021). The quantities for these compatible solutes are particularly fine-tuned at optimal 1 M NaCl in comparison with saturated NaCl (5.13 M), with trehalose being the most responsive solute in relation to osmotic changes (Jiménez-Gómez et al., 2020).

The objective of this work is to analyze the osmoadaptations to saturated NaCl concentration in *A. sydowii*. This study is the first multi-omics approach to unravel adaptations to saturated NaCl concentration in an ascomycetous fungus. Thus, the halophilic *A. sydowii* enabled us to focus on molecular adaptations occurring at saturated salt concentrations inhibitive for most fungi by using mRNA transcriptional profiles and gene ontology analysis, measuring lncRNAs and transcriptional factors, conducting differential analysis of amino acids and fatty acids as well as metabolomic profiling.

## Materials and Methods

### Strain Source, Preservation, and Culture Conditions

A halophilic fungal strain (EXF-12860) of *A. sydowii*, isolated from a solid fermentation of sugarcane bagasse in the presence of 2 M NaCl, was used in this work (Batista-García et al., 2014). Fungal cultures were propagated on Yeast Malt Agar (YMA): malt extract 10 g/L, yeast extract 4 g/L, dextrose 4 g/L, mycological peptone 5 g/L, agar 20 g/L. Spores were cryopreserved at −80°C in 20% glycerol and deposited in the Ex Microbial Culture Collection of the Infrastructural Centre Mycosmo, University of Ljubljana, Slovenia.

Two different hypersaline (NaCl) experimental conditions were used for growth of EXF-12860: optimal at 1 M NaCl (*a_w_* = 0.98) and saturated 5.13 M NaCl solution (*a_w_* = 0.75). Omics analysis (i.e., transcriptomics and metabolomics) and determinations of amino acids and fatty acids were performed at both NaCl concentrations. Spores and mycelium obtained from 7-day-old cultures of EXF-12860 supplemented with 1 M NaCl were used as pre-inoculum in all experiments. After 7 days in the presence of 1 M NaCl the liquid cultures were in the last part of the exponential phase (Rodríguez-Pupo et al., 2021). Later, mycelium was transferred to a fresh medium for both conditions (1 M and 5.13 M NaCl) and incubated at 28°C and 150 rpm. In the “control” condition (1 M NaCl), cells were not subjected to osmotic shock, but only to a renewed source of nutrients. In the “treatment” condition, cells were additionally exposed to a hypersaline medium (5.13 M NaCl), which should trigger a transient transcriptional program for immediate response and a sustained transcriptional program for long-term adaptation mechanisms. A growth curve based on the fungal dry weight was performed (Supplementary Figure S1) under optimal salinity condition (1 M NaCl), and we determined that the culture reaches the middle exponential growth phase after 4 days. Mycelia and supernatants obtained from triplicate exponential phase cultures of the fungus (4-day-old cultures) were separated by centrifugation at 10,000 × *g* and used for the subsequent experiments.

### RNA Extraction, Library Construction, Sequencing, and Transcriptomic Analysis of *Aspergillus sydowii* at Hypersaline Conditions

Fungal mycelium was collected by centrifugation and ground in liquid nitrogen. Total RNA was extracted using the TRIzol method (Chomczynski and Sacchi, 2006). After performing RNA quality control (RNA integrity number, RIN > 7) by capillary electrophoresis using an Agilent Technology Bioanalyzer 2,100, RNA samples were selected to proceed to library construction using TruSeq Stranded mRNA LT Sample Prep Kit (Illumina, Catalogue No. RS-122-2101). For this, rRNAs were depleted by oligo-dT selection (Blower et al., 2013) and cDNA was synthetized. The sequencing libraries were prepared by random fragmentation of cDNA sample, followed by 5′ and 3′ adapter ligation. Adapter-ligated fragments were amplified by PCR, purified by gels, and the size of PCR enrichment fragments was verified through the template size distribution obtained in an Agilent Technologies 2,100 Bioanalyzer using a DNA 1000 chip. Libraries were quantified using qPCR according to the Illumina qPCR quantification protocol guide. Sequencing (2 × 150 bp paired-end) was performed in triplicates (*n* = 3) on the Illumina HiSeq-2,500 platform by Macrogen Company (Seoul, South Korea), resulting in a minimum of 70 million reads per sample.

Sequencing quality control was performed using Trimmomatic version 0.39 (Bolger et al., 2014). Low-quality reads, 5′ and 3′ adaptors, and highly overrepresented sequences were removed after demultiplexing. Error correction for Illumina RNA sequencing reads was made using Rcorrector (Song and Florea, 2015). *De novo* assembly algorithm (Trinity version 2.10.0) was run to assemble short-nucleotide RNA sequencing reads into contigs (Grabherr et al., 2011). BUSCO methodology (version 4.0.5) was employed to evaluate the transcriptome completeness (Seppey et al., 2019). Blast2GO was used to generate high-quality functional annotations and the analysis of transcriptomic data (Biobam Bioinformatic, 2019). Briefly, a blast-based workflow using Fungi section in nr database was addressed. Gene ontology (GO) and InterPro annotations were obtained from this high-throughput workstation. Kallisto version 0.46.1 was used to quantify abundances of transcripts (Bray et al., 2016). Unwanted variations in RNA sequencing reads were removed from unnormalized counts using RUVseq (Risso et al., 2014). Differential gene expression analysis was obtained using edgeR version 1.29.5 (Love et al., 2014). GO enrichment was performed using Blast2GO in OmicsBox (Biobam Bioinformatic, 2019), which uses a Fisher’s exact test to determine the false discovery rate (FDR) of GO enrichment. Finally, pathway enrichment analysis was conducted on KEGG (Kyoto Encyclopedia of Genes and Genomes) mapper using the KEGG annotations retrieved from GhostKOALA (Kanehisa et al., 2021).

Long non-coding RNAs (lncRNAs) were also identified using a custom pipeline to identify candidate lncRNAs. Firstly, we assessed the coding potential of all the differentially expressed transcripts (logFC > 1.5, value of *p* < 0.05, FDR < 0.05, and CPM < 0.05) using RNAsamba (Camargo et al., 2020) and CPC2 (Kang et al., 2017). Transcripts classified as non-coding using both tools were identified as differentially expressed lncRNAs. Transcripts classified as coding using both tools were identified as differentially expressed mRNAs. Finally, we used the previously mentioned expression parameters with transcripts annotated as transcription factors (TFs) to identify differentially expressed genes encoding TFs.

To analyze the possible regulatory function of the identified differentially expressed both lncRNAs and genes encoding TFs, we performed a co-expression analysis of those lncRNAs, TFs, and mRNAs differentially expressed (Zhan et al., 2016), based on the Pearson correlation coefficient (*r*) using the R package Hmisc (Pasek, 2020). We selected pairs of mRNA/lncRNA or mRNA/TF with a |*r*| > 0.9 and a value of *p* < 0.05. The same procedure was conducted to obtain the co-expression network to correlate those GO identified as enrichened.

Transcriptomic dataset was deposited for public availability in National Centre for Biotechnology Information (NCBI) under the submission IDs: SUB8102769, BioProject PRJNA662826, BioSample accession: SAMN16095160.

### Amino Acid and Fatty Acid Determinations at Hypersaline Conditions

The crude protein content of the mycelium and cell-free culture medium of each hypersaline growth condition was determined according to the Lowry method (Lowry et al., 1951).

The amino acid content was determined from minced (for mycelium) and homogenized samples which were previously defatted using Soxhlet extraction according to AOAC (1995). To 10 mg of sample, 5 ml of 6 N HCl containing 0.06% phenol was added. To produce free amino acids the mixtures were hydrolyzed for 24 h at 110°C under nitrogen atmosphere to avoid oxidation. The hydrolyzed samples were diluted in 25 ml of deionized water, and 1 ml of 2.5 mM α-amino butyric acid (AABA) was added as an internal standard. Samples were then filtered through a 0.45-μm Teflon filter and stored under nitrogen atmosphere at −30°C. Samples containing the free amino acids were derivatized (Bidlingmeyer et al., 1984), injected into an Agilent 1,260 Infinity High-Performance Liquid Chromatography (HPLC) system (Agilent Technologies, CA, United States) equipped with an Agilent C-18 reversed-phase Zorbax Eclipse AAA column (i.d. 3.5 μm particle, 4.6 × 150 mm), and eluted using an acetonitrile–methanol–water (45:45:10, *v*/*v*) gradient. Column temperature was maintained at 40°C, and a flow of 1 ml/min was used. Injection volume was 5 μl. Samples were monitored using a 1,260 fluorescence detector (FLD, Agilent Technologies, CA, United States) in series. Fluorescence chromatograms were obtained using an excitation wavelength of 340 nm and monitored emission signals at 450 nm. Standard curves were obtained for the amino acid determination using amino acid standard solutions in the range from 25 to 350 pmol (P.N. 061-3330, Agilent Technologies, CA, United States). Agilent OpenLAB ChemStation version C.01.07 software was used for the amino acid quantification. This method does not detect the amino acids glutamine, aspartate, and tryptophan.

Fatty acid methyl ester extracts were obtained from samples of mycelium and cell-free culture medium of each growth hypersaline condition (Folch et al., 1957; Christie, 1993). Fatty acid methyl esters were separated in an Agilent Technologies 7820A Gas Chromatograph (Agilent Technologies, CA, United States) equipped with a flame ionization detector (260°C) and a capillary column (DB-23 Agilent; 60 m × 0.25 mm, film thickness 0.15 μm) using nitrogen as the carrier gas. The initial oven temperature was 120°C. One microliter of the solution containing the fatty acid methyl esters was injected split 10:1. After 1 min the temperature was increased at a rate of 6.5°C/min to 170°C, immediately later, increased at a rate of 6°C/min to 198°C holded by 7 min, and finally increased at a rate of 15°C/min to 230°C holds by 5 min and then maintained at that temperature for an additional 10 min. Fatty acids were identified by comparing the retention times of well-characterized profiles of fatty acid methyl ester standards (37 component fatty acid methyl ester Mix, Supelco/Sigma-Aldrich, CA, United States). The concentration of each fatty acid was determined from the corresponding area within the chromatogram using a C19 fatty acid as internal standard and the software package Agilent OpenLAB ChemStation version C.01.07.

Differences (*p* ≤ 0.05) among the mean amounts of amino acids and fatty acids were determined by Kruskal–Wallis test and Dunn’s test as *post hoc* analysis. Statistical calculations were performed using PRISM 8 (GraphPad software, CA, United States).^1^

### Metabolomics Profiling of *Aspergillus sydowii* EXF-12860 Grown at Optimal and Saturated NaCl Concentrations

One milliliter of cell-free extracts or control media was lyophilized. Then, 500 μl of cold acetone was added to the dry samples, vortex for 1 min, and centrifuged at 14,000 rpm for 10 min at 4°C. These steps were repeated twice and 90% of the solvent volume at each step was collected and combined in a 1.5-mL Eppendorf tube. The solvent was evaporated using a SpeedVac system (Thermo Fisher Scientific, MA, United States) at room temperature. Metabolites were resuspended in 50 μl of a solution of water:acetonitrile at a 95:5 ratio. Quality control samples were prepared by mixing equal volumes of all samples.

We followed the HPLC-Mass spectrometry in Tandem (LC–MS^2^) methodology previously reported with minor modifications (Moreno-Ulloa et al., 2020). In brief, 10 μl of samples was randomly loaded into the Eksigent nanoLC400 system (Eksigent AB Sciex, CA, United States) with a HALO Phenyl-Hexyl column (0.5 × 50 mm, 2.7 mm, 90 Å pore size). Metabolites were separated using a gradient elution with 0.1% formic acid in water (A) and 0.1% formic acid in acetonitrile (B) as mobile phases at a constant flow rate of 5 ml/min. The gradient started at 5% B for 1 min followed by a stepped increased to 100% B over 26 min and held constant for 4 min. Solvent composition was then returned to 5% B over 0.1 min. To ensure column re-equilibration, a 4-minute pre-run with 5% B was applied between samples. A blank sample (10 μl of A:B solution at a 95:5 ratio) was run between experimental sample injections to minimize potential carryover. The eluate from LC was delivered directly to the TurboV source of a TripleTOF (time-of-flight) 5,600+ mass spectrometer (SCIEX, CA, United States) using electrospray ionization (ESI) under positive mode. ESI source conditions were set as follows: IonSpray voltage floating, 5,500 V; source temperature, 350°C; curtain gas, 20 psi; ion source gases 1 and 2 were set to 40 and 45 psi; declustering potential, 100 V. Data were acquired using data-dependent acquisition (DDA) with high-sensitivity mode selected, automatically switching between full-scan MS and MS^2^. The accumulation time for TOF MS was 0.25 s/spectra over the *m/z* range 100–1,500 Da, and for MS^2^ scan was 0.05 s/spectra over the *m/z* 50–1,500 Da. The DDA settings were as follows: charge state +1 to +2, intensity 125 cps, exclude isotopes within 6 Da, mass tolerance 50 mDa, and a maximum number of candidate ions 20. Under IDA settings, the “exclude former target ions” was set as 15 s after two occurrences and “dynamic background subtract” was selected. The manufacturer rolling collision energy (CE) option was used based on the size and charge of the precursor ion using formula CE = *m/z* × 0.0575 + 9. The instrument was automatically calibrated by the batch mode using appropriate positive TOF MS and MS^2^ calibration solutions before sample loading and after injection of six samples (<3.5 working hours) to ensure a mass accuracy of <5 ppm for both MS and MS/MS data. Instrument performance was monitored during data acquisition by including one quality control sample (*n* = 4) every four experimental samples.

Two complementary informatic approaches were utilized to analyze the LC-MS^2^ datasets: (i) Feature extraction, normalization, and statistical analysis (univariate and multivariate analysis) were performed using MarkerView TM software version 1.3 (AB Sciex, CA, United States), and (ii) MS^2^ spectral data extraction for metabolite identification or annotation [level 2 in agreement with the Metabolomics Standards Initiative (MSI) classification; Aron et al., 2020] was performed using MZmine software version 2.53 and the Global Natural Products Social Molecular Networking web platform (GNPS; Aron et al., 2020).^2^ Furthermore, to overcome the limited identification or annotation of metabolites by spectral matching against the GNPS spectral libraries, we utilized the advanced annotation tool of SIRIUS 4 and CSI:Finger ID (level 3 in agreement with the MSI classification; Dührkop et al., 2015).

All experiments were performed in triplicate. For the metabolomics data, taking into account the fold-change compression phenomenon (Yu et al., 2020), features with a fold change ≥1.5 or ≤−1.5, value of *p* < 0.05 (*t*-test) were considered as differentially abundant. For multivariate statistical analysis and to assess for sample clustering behavior and inter-group variation, we utilized principal component analysis (PCA) and principal component variable grouping (PCVG; Ivosev et al., 2008). Log2-transformed data were used for PCA and PCVG analysis. Software PRISM 6.0 (GraphPad software, CA, United States) was used for the creation of volcano plots.

The raw datasets have been deposited on the GNPS/MassIVE public repository (Wang et al., 2016) under the accession number MSV000088136. The parameters for classical molecular networking using all datasets are available on the following link: https://gnps.ucsd.edu/ProteoSAFe/status.jsp?task=a3e71334eb534f89b24b09f31c9bca5d. The NAP job is here: https://proteomics2.ucsd.edu/ProteoSAFe/status.jsp?task=a27935fe261946169af78d93d8d6e312. The MolNetEnhancer job is here: https://gnps.ucsd.edu/ProteoSAFe/status.jsp?task=d974315d59b649be96e6bf583505f2aa. The parameters for FBMN (*A. sydowii* 1 M vs. 5.13 M NaCl) are available on the following link: https://gnps.ucsd.edu/ProteoSAFe/status.jsp?task=5f212d5ccb1a46de86fe6ba4fb402952. The Qemistree job is available in the following link: https://proteomics2.ucsd.edu/ProteoSAFe/status.jsp?task=80ba69060ca54408b52ebf726b91359c.

## Results

### mRNA Transcriptional Profiles and Gene Ontology Analysis Revealed the Importance of Metabolism and Signal Transduction at Saturated NaCl Concentration

To provide deeper insights into the molecular mechanisms of adaptation of the halophilic *A. sydowii* EXF-12860 to extremely low water activity due to high NaCl, we analyzed the mRNA profiles obtained from three independent YMA liquid cultures of *A. sydowii* in exponential phase, grown at optimal, and saturated NaCl concentration. Analysis revealed 41,309 transcribed sequences and 1,842 genes differentially expressed (logFC > 1.5, value of *p* < 0.05) at NaCl saturation as compared to optimal NaCl concentration. In the transcriptional profile of EXF-12860, 704 genes were overexpressed, and 1,138 genes downregulated (Supplementary Tables S1 and S2). Eighty-nine percent of the genes differentially expressed could be annotated with KEGG, while 193 (1.25%) genes encode for proteins with unknown functions.

**Figure 1:**
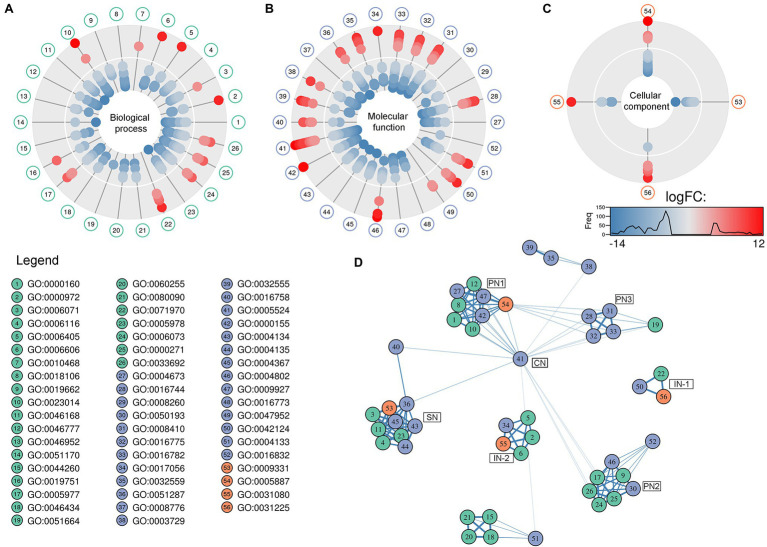
Pie-donuts chart **(A–C)** showing the gene ontology (GO) term enrichment from the overrepresented functional annotations in the set of differentially expressed transcripts of *Aspergillus sydowii* exposed to extreme water deprivation by salt (5.13 M NaCl) compared to optimal salt concentration for growth (1 M NaCl). GOs are named using numbers from 1 to 56. **(A)** Biological process (green circles, 1–26). **(B)** Molecular function (blue circles, 27–52). **(C)** Cellular component (red circles, 53–56). White circle inside each donut represents logFC = 0. The transcripts associated with each GO are represented based on their logFC value as: upregulated transcripts (logFC > 1.5) are red filled circles inside the donuts; downregulated transcripts (logFC < −1.5) are blue filled circles inside the donuts. **(D)** Multi-scale network obtained from the GO enrichment data. One central node (CN), three principal nodes (PN1, PN2, and PN3), one secondary node (SN), and two isolated nodes (IN) are observed. Only the GO sharing more than three transcripts between them are represented in the GO term-based network. GO legend: Biological process: 1-GO:0000160 phosphorelay signal transduction system; 2-GO:0000972 transcription-dependent tethering of RNA polymerase II gene DNA at nuclear periphery; 3-GO:0006071 glycerol metabolic process; 4-GO:0006116 NADH oxidation; 5-GO:0006405 RNA export from nucleus; 6-GO:0006606 protein import into nucleus; 7-GO:0010468 regulation of gene expression; 8-GO:0018106 peptidyl-histidine phosphorylation; 9-GO:0019662 non-glycolytic fermentation; 10-GO:0023014 signal transduction by protein phosphorylation; 11-GO:0046168 glycerol-3-phosphate catabolic process; 12-GO:0046777 protein autophosphorylation; 13-GO:0046952 ketone body catabolic process; 14-GO:0051170 import into nucleus; 15-GO:0044260 cellular macromolecule metabolic process; 16-GO:0019751 polyol metabolic process; 17-GO:0005977 glycogen metabolic process; 18-GO:0046434 organophosphate catabolic process; 19-GO:0051664 nuclear pore localization; 20-GO:0060255 regulation of macromolecule metabolic process; 21-GO:0080090 regulation of primary metabolic process; 22-GO:0071970 fungal-type cell wall (1,3)-β-D-glucan biosynthetic process; 23-GO:0005978 glycogen biosynthetic process; 24-GO:0006073 cellular glucan metabolic process; 25-GO:0000271 polysaccharide biosynthetic process; 26-GO:0033692 cellular polysaccharide biosynthetic process. Molecular function: 27-GO:0004673 protein histidine kinase activity; 28-GO:0016744 transferase activity, transferring aldehyde or ketonic groups; 29-GO:0008260 3-oxoacid CoA-transferase activity; 30-GO:0050193 phosphoketolase activity; 31-GO:0008410 CoA-transferase activity; 32-GO:0016775 phosphotransferase activity, nitrogenous group as acceptor; 33-GO:0016782 transferase activity, transferring sulfur-containing groups; 34-GO:0017056 structural constituent of nuclear pore; 35-GO:0032559 adenyl ribonucleotide binding; 36-GO:0051287 NAD binding; 37-GO:0008776 acetate kinase activity; 38-GO:0003729 mRNA binding; 39-GO:0032555 purine ribonucleotide binding; 40-GO:0016758 hexosyltransferase activity; 41-GO:0005524 ATP binding; 42-GO:0000155 phosphorelay sensor kinase activity; 43-GO:0004134 4-α-glucanotransferase activity; 44-GO:0004135 amylo-α-1,6-glucosidase activity; 45-GO:0004367 glycerol-3-phosphate dehydrogenase [NAD+] activity; 46-GO:0004802 transketolase activity; 47-GO:0009927 histidine phosphotransfer kinase activity; 48-GO:0016773 phosphotransferase activity, alcohol group as acceptor; 49-GO:0047952 glycerol-3-phosphate dehydrogenase [NAD(P)+] activity; 50-GO:0042124 1,3-β-glucanosyltransferase activity; 51-GO:0004133 glycogen debranching enzyme activity; and 52-GO:0016832 aldehyde-lyase activity. Cellular component: 53-GO:0009331 glycerol-3-phosphate dehydrogenase complex; 54-GO:0005887 integral component of plasma membrane; 55-GO:0031080 nuclear pore outer ring; and 56-GO:0031225 anchored component of membrane.

The GO analysis showed 26 biological process, 26 molecular function, and four cellular component GO terms significantly enriched (value of *p* < 0.05, Fisher’s test; Figures 1A–C). Enriched biological processes, overrepresented according to the mRNA profiles, were the phosphorelay signal transduction system, RNA and protein traffic across nucleus, regulation of gene expression, regulation of macromolecules and primary metabolism, non-glycolytic fermentation, polysaccharide biosynthesis, cell wall β-glucan biosynthesis, polyol metabolism (i.e., glycerol metabolism), ketone body catabolism, and NADH oxidation (Figure 1A).

In correlation with the enriched biological processes, our transcriptome data revealed that transferase activity of CoA, glycans, ketonic, aldehyde, phosphate, and sulfur-containing groups, together with NAD, mRNA and ATP binding, and phosphorelay sensor kinase processes were the molecular function GO categories with the highest number of differentially expressed genes (Figure 1B). The set of genes that ensure glycerol-3-phosphate dehydrogenase activity was mainly downregulated (Figure 1B), which is consistent with the low glycerol concentrations at 5.13 M NaCl reported in previous studies of our group (Jiménez-Gómez et al., 2020). This result is very interesting as it is completely opposite to the response to salt stress in other fungi (Zajc et al., 2014; Gunde-Cimerman et al., 2018; Turk and Gostinčar, 2018; Lee et al., 2021). Finally, the overrepresented cellular component categories were related to the membrane structural components, outer ring of nuclear pore, and glycerol-3-phosphate dehydrogenase complex (Figure 1C). In conclusion, the GO category analysis exposed that most differentially expressed genes were involved in primary metabolism and signal transduction.

A GO multi-scale network was obtained from the GO enrichment data (Figure 1D). Illustration of the GO-derived network displayed that the GO term ATP binding constitutes the main network node and shows direct interactions between the central node (CN) and three other principal nodes (PN). The first node (PN1) harbors GO related to phosphorelay signal transduction system, protein autophosphorylation, signal transduction by protein phosphorylation, phosphorelay sensor kinase activity, and phosphotransferase kinase activity. PN2 harbors cellular polysaccharide metabolism processes and non-glycolytic fermentation and PN3 transferase activity of CoA and groups containing sulfur, nitrogen, and phosphorous (Figure 1D). A secondary node (SN) related to metabolism of glycerol, NAD, and carbohydrate was also observed. Moreover, two isolated nodes (IN) were identified, mainly related to: (IN-1) cell wall component metabolism and anchored component of membrane, and (IN-2) processes related to RNA export from nucleus, protein export into nucleus, and structural constituents of nuclear pore.

### Reprogramming of Amino Acid Metabolism Occurred at Saturated NaCl Concentration

**Figure 2:**
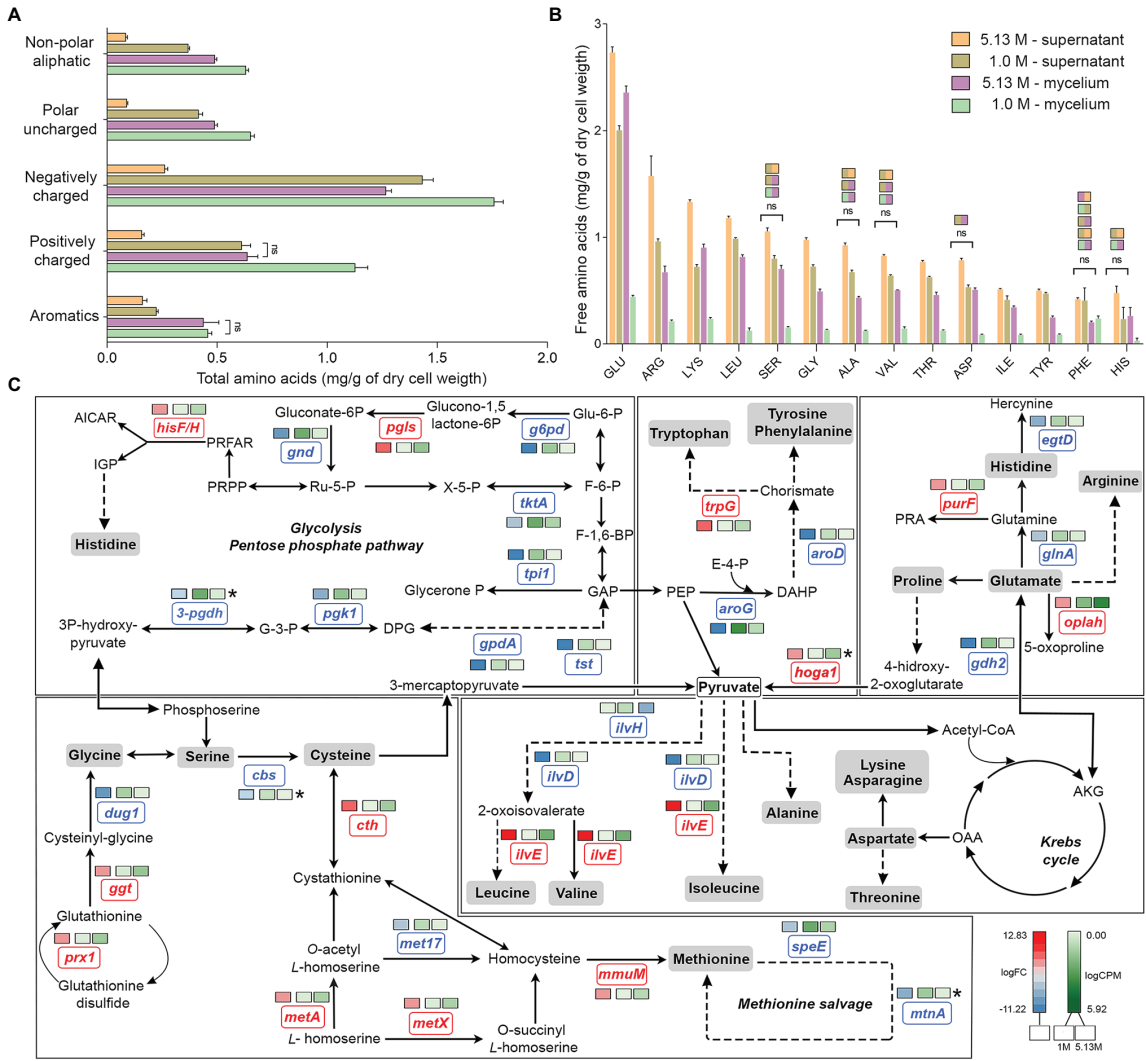
Quantitative profile of amino acids and differential expression of genes related to amino acid metabolism in *A. sydowii*. **(A)** Bar chart showing the free amino acids (mg/g of dry cell weight) in both mycelia and supernatants obtained from *A. sydowii* cultured at 1 M and 5.13 M NaCl. Amino acids are grouped by their side chain characteristics. Except as otherwise noted (ns), all pairwise comparisons are statistically significant by Kruskal–Wallis test—Dunn’s test as *post hoc* analysis. **(B)** Bar chart showing the free amino acids (mg/g of dry cell weight) in both mycelia and supernatants obtained from 1 M and 5.13 M NaCl. All pairwise comparisons are statistically significant by Kruskal–Wallis test—Dunn’s test as *post hoc* analysis—except those marked in the upper color boxes. **(C)** Metabolic map showing differentially expressed genes (condition of 5.13 M NaCl compared to 1 M NaCl) involved in amino acid biosynthesis pathways. Red and blue boxes/letters indicate genes upregulated (logFC > 1.5) and downregulated (logFC < −1.5), respectively. Asterisks indicate that two or more transcript isoforms were identified for that gene. The three squares above each gene box illustrate the logFC values and counts per million (CPM) of transcripts in each salt concentration. Dashed and continuous lines refer to intermediate and direct reactions, respectively. AICAR, 5’-phospho-ribosyl-5-amino-4-imidazole carboxamide; IGP, imidazole glycerol-3P; PRFAR, phosphoribulosyl-formiminio-AICAR-P; PRPP, 5-phosphoribosyl diphosphate; Ru-5-P, ribose-5-phosphate; X-5-P, xilulose-5-phosphate; Glu-6-P, glucose-6-phosphate; F-6-P, fructose-6-phosphate; F-1,6-BP, fructose-1,6-biphosphate; GAP, glyceraldehyde-3-phosphate; PEP, phosphoenolpyruvate; E-4-P, erythrose-4-phosphate; DAHP, 2-dehydro-3-deoxy-arabino-heptonate-7-phosphate; PRA, 5-phosphoribosylamine; OAA, oxaloacetate; and AKG, α-ketoglutarate.

Overall, the amino acid profiles differed significantly (Kruskal–Wallis, value of *p* < 0.05) in optimal and hypersaline conditions. Supernatant of mycelium grown at optimum NaCl concentration displayed higher amounts of free amino acids (Figures 2A,B) in comparison with amounts at NaCl saturation, suggesting decreased synthesis and secretion of extracellular proteins. While the global level of aromatic amino acid was similar in mycelia grown at both optimal and saturated salinity, the amounts of charged and uncharged amino acids and non-polar aliphatic amino acids decreased significantly (Kruskal–Wallis, value of *p* < 0.05) in mycelia exposed to saturated concentration of NaCl (Figure 2A).

The analyses of individual free amino acids composition in the *A. sydowii* cells revealed higher amounts of glutamate at both optimal and saturated NaCl conditions, in both mycelium and supernatant (Figure 2B). Glutamate, arginine, and lysine were the most abundant, similarly as in the halotolerant yeast *H. burtonii* exposed to 1 M NaCl (Lee et al., 2021). Serine, glycine, alanine, valine, histidine, and phenylalanine level did not change in mycelia grown both at optimal salinity and at saturated NaCl condition (Figure 2B), as well as in the respective supernatants. The amino acids cysteine, methionine, and proline were not detected. Globally, the free amino acids were enriched 3.5-fold in supernatants obtained from cultures at optimal salinity compared to supernatants of cultures in saturated NaCl concentration (Figure 2B).

The mRNA transcriptional analysis of the amino acid metabolism revealed 33 differentially expressed genes, including 20 downregulated genes and 13 overexpressed genes at saturated NaCl condition in comparison with optimal 1 M NaCl concentration (Figure 2C; Supplementary Table S3). Although genes related to the synthesis of histidine (*hisF/H*, logFC = 3.98), tryptophan (*trpG*, logFC = 5.63), glycine (*prx1*, logFC = 4.73; *ggt*, logFC = 4.27), cysteine (*cth*, logFC = 7.20; metA, logFC = 3.69), methionine (*metX*, logFC = 3.69; *mmuM*, logFC = 4.75), and leucine, valine, and isoleucine (*ilvE*, logFC = 12.83) were upregulated at saturated NaCl concentration (Figure 2C), the amount of free amino acids did not increase, indicating that transcript and protein levels do not always correlate.

Gene *hoga1* that encodes the enzyme 4-hydroxy-2-oxoglutarate aldolase was overexpressed (logFC = 7.53) at saturated NaCl condition (Figure 2C). This aldolase catalyzes the conversion of 4-hidroxy-2-oxoglutarate into pyruvate increasing the pyruvate availability and driving metabolic flux through the Krebs cycle and probably to biosynthesis of amino acids (Huang et al., 2019).

The transcriptomic analysis revealed a set of upregulated genes involved in methionine and cysteine biosynthesis pathways (Figure 2C). Gene *mmuM* that encodes homocysteine *S*-methyltransferase involved in methionine formation was transcriptionally activated (logFC = 4.75) at saturated NaCl concentration (Figure 2C). The *cth* gene that encodes cystathionine γ-lyase, the enzyme that catalyzes the last step in the transsulfuration pathway from cystathionine to cysteine, was also upregulated (logFC = 7.20; Figure 2C). Other genes such as *metX* and *metA* which encode homoserine acetyltransferase (MetX) and homoserine succinyltransferase (MetA), respectively, were also overexpressed at saturated NaCl condition (logFC = 3.69 and logFC = 3.67, respectively; Figure 2C).

Gene *hisF/H* involved in histidine biosynthesis pathway was also upregulated (logFC = 3.98; Figure 2C). Imidazole glycerol phosphate (IGP) synthase, the *hisF/H* product, catalyzes the conversion of *N*′-(5′-phosphoribosyl)-formimino-5-aminoimidazole-4-carboxamide ribonucleotide (PRFAR) into IGP and 5′-(5-aminoimidazole-4-carboxamide) ribonucleotide (AICAR), connected to both histidine synthesis and nitrogen metabolism and to the *de novo* purine biosynthesis pathway. Finally, gene *ilvE* involved in the biosynthesis of valine, leucine, and isoleucine was also overexpressed (logFC = 12.83; Figure 2C).

### Fatty Acid Metabolism at Saturated NaCl Concentration Remained Mainly Unchanged

**Figure 3:**
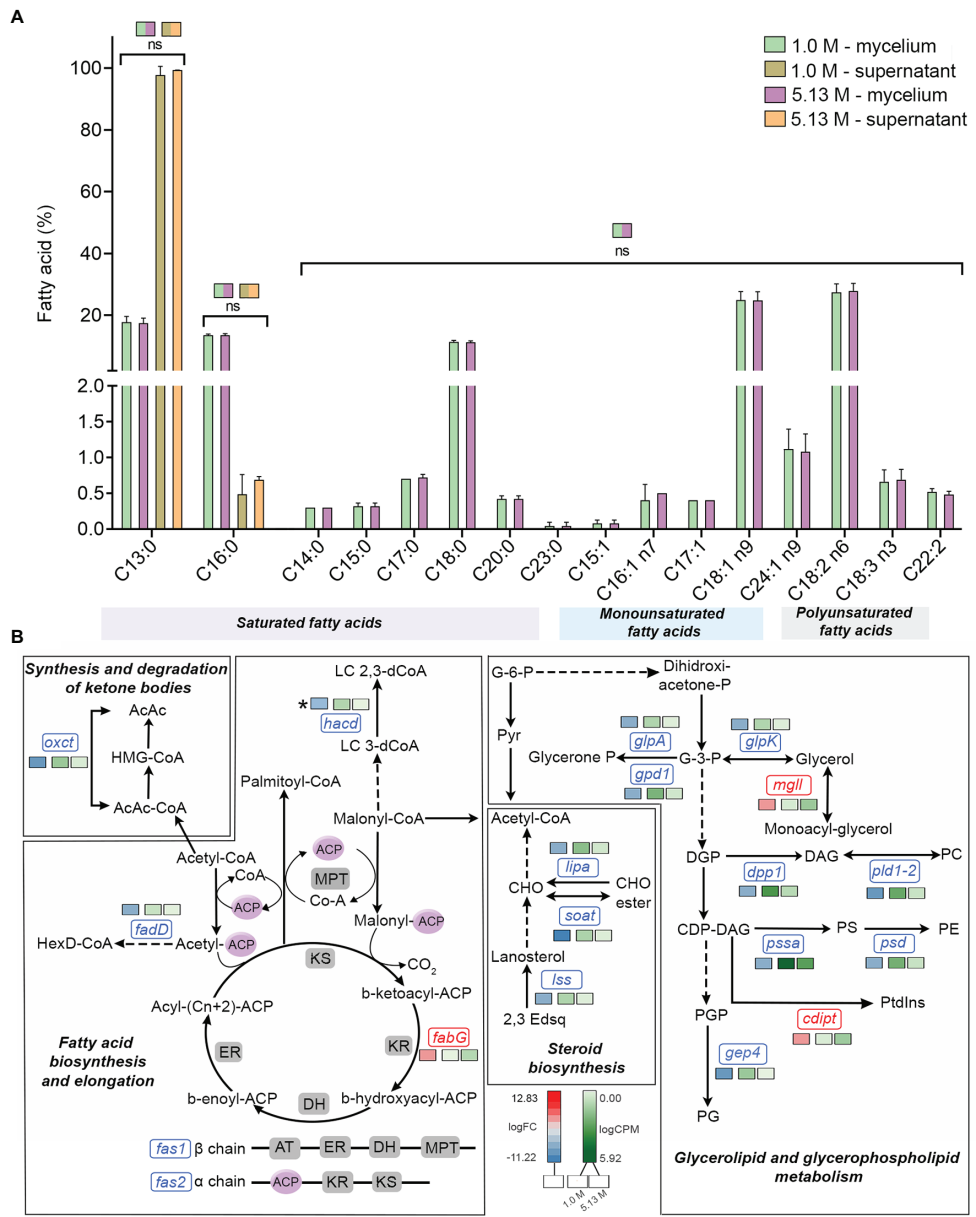
Quantitative profile of fatty acids and differential expression of genes related to fatty acid metabolism in *A. sydowii* exposed to 5.13 M NaCl compared to 1 M NaCl. **(A)** Bar chart showing the percentage of saturated (C13:0–C23:0), monounsaturated (C15:1–C24:1 n9), and polyunsaturated (C18:2 n6–C22:2) in both mycelia and supernatants collected from fungal cultures at 1 M and 5.13 M NaCl. In **(B)** the statistics shown refer to the comparisons that were not statistically significant (ns); thus, the rest of the comparisons are statistically significant. **(B)** Metabolic map showing differentially expressed genes involved in fatty acid biosynthesis pathways in *A. sydowii* cultured at 5.13 M NaCl compared to 1 M NaCl. Red and blue boxes/letters indicate genes upregulated (logFC > 1.5) and downregulated (logFC < −1.5), respectively. Asterisks indicate that two or more transcript isoforms were identified for that gene. The three squares above each gene box illustrate the logFC values and CPM of transcripts in each salt concentration. Dashed and continuous lines refer to intermediate and direct reactions, respectively. AcAc, acetoacetyl-CoA; HMG-CoA, S-3-hydroxy-3-methylglutaryl-CoA; HexD-CoA, hexadecanoyl-CoA; LC 2,3-dCoA, long-chain trans-2,3-dehydroacyl-CoA; LC 3-dCoA, long-chain-3-hydroxyacyl-CoA; G-6-P, glucose-6-phosphate; Pyr, pyruvate; CHO, cholesterol; 2,3-Edsq, S-squalene; G-3-P, glycerol-3-phosphate; DGP, 1,2-diacyl-glycerol-3P; DAG, 1,2-diacyl-glycerol; CDP-DAG, CDP-diacylglycerol; PGP, phosphatidyl-glycerophosphate; PG, phosphatidylglycerol; PC, phosphatidylcholine; PE, phosphatidylethanolamine; PS, phosphatidylserine; Ptdlns, phosphatidyl-1D-myo-inositol; ACP, acyl carrier protein; ER, β-enoyl reductase; DH, dehydratase; KR, β-ketoacyl reductase; KS, β-ketoacyl synthase; and MPT, malonyl/palmitoyl transferase.

Surprisingly in *A. sydowii* no modifications of fatty acid composition occurred neither at optimal nor at saturated NaCl concentrations (Figure 3A). Tridecanoic acid (C13:0) and palmitic acid (C16:0) were detected in both supernatants and mycelia of *A. sydowii* grown at both optimal NaCl and saturated NaCl concentrations, while the other fatty acids were found only in mycelia (Figure 3A). Oleic acid (C18:0) and its unsaturated derivates *cis*-9-octadecenoic acid (C18:1 n9), linoleic acid (C18:2 n6), and α-linolenic acid (C18:3 n3) represented on average 65% of the global fatty acid composition, reflecting the important physiological role of oleic acid in supporting fungal growth at hypersaline conditions. Overall, C18 monounsaturated and polyunsaturated fatty acids represented at both optimal and saturated NaCl concentrations 55% of the total C18 fatty acid composition, with C18:1 n9 and C18:2 n6 as the most prevalent (Figure 3A).

The transcriptomic data in *A. sydowii* grown at saturated NaCl concentration revealed that the fatty acid biosynthesis and elongation, synthesis and degradation of ketone bodies, steroid biosynthesis, and glycerolipid and glycerophospholipid metabolism were turned off transcriptionally (Figure 3B; Supplementary Table S4). Only the 3-ketoacyl-acyl carrier protein (3-ketoacyl-ACP) reductase (*fabG*), monoacyl glycerol lipase (*mgll*), and phosphatidylinositol synthase (*cdipt*) genes were upregulated (logFC = 4.26, logFC = 3.88, and logFC = 4.04, respectively; Figure 3B). In *A. sydowii* transcriptome *hacd* (logFC = −5.67), *fadD* (logFC = −4.31), and *fas1*/*fas2* (logFC = −6.92/−4.47) genes that encodes 3-hydroxyacyl-CoA dehydratase, inner membrane-associated acyl-CoA synthase, and fatty acid synthase complex, respectively, were repressed at saturated NaCl concentration (Figure 3B). Genes *hacd* and *fadD* are involved in fatty acid elongation (Fujita el at., 2007), while *fas* gene products catalyze all C16-C18 fatty acid biosynthesis (Runguphan and Keasling, 2014).

### Analysis of Molecular Adaptations at Saturated NaCl Exposed the Importance of High-Osmolarity Glycerol Signal Transduction Pathway and Cell Wall Ultrastructure and Morphology

**Figure 4:**
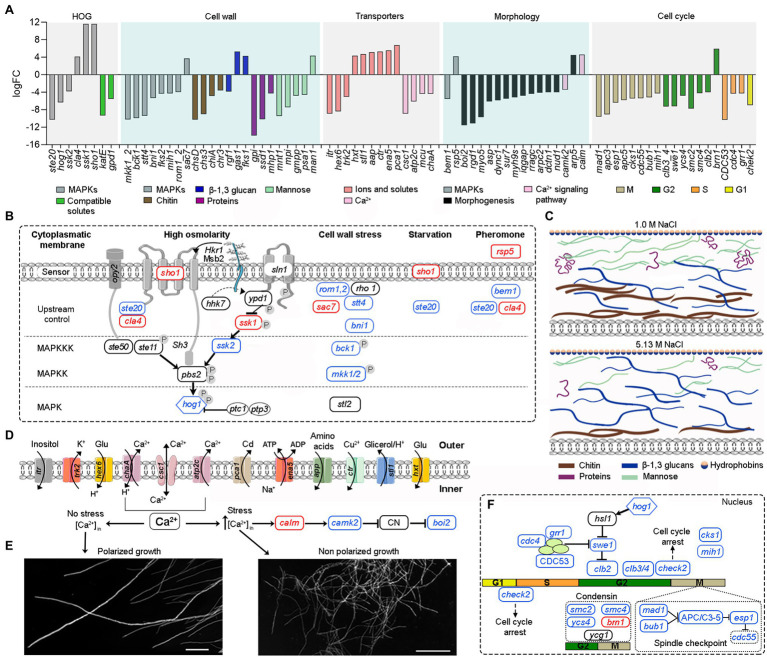
Overview of the main tolerance mechanisms of *A. sydowii* cultured at saturated NaCl concentration (5.13 M NaCl) compared to optimal salt concentration for growth (1 M NaCl). **(A)** Bar graph showing the logFC values of all differentially expressed genes involved in high osmolarity glycerol pathway (HOG), cell wall biosynthesis, cellular membrane transporters, mycelium morphology, and cell cycle. **(B)** Genes related to the MAPK signaling pathways that respond to a hyperosmolarity condition. Red, blue, and black boxes/letters indicate genes upregulated, downregulated, and non-differentially expressed, respectively. **(C)** Composition of the cell wall in the two conditions studied inferred from the expression of genes involved in the synthesis of the cell wall components (chitin, proteins—hydrophobins are shown particularly—β-13-glucans and mannose). **(D)** Differentially expressed cytoplasmic membrane transporters at saturated NaCl concentration. **(E)** Electron microscopy of *A. sydowii* cultured at 1 M NaCl (polarized growth) and 5.13 M NaCl (non-polarized growth). Scale bars = 100 μm. **(F)** Differentially expressed genes related to the cell cycle.

An overview of differentially expressed genes at saturated NaCl concentration is presented in Figure 4; Supplementary Table S5.

In *A. sydowii sho1* (logFC = 11.65), *cla4* (logFC = 4.15), and *ssk1* (logFC = 11.65) were upregulated at saturated NaCl concentration, while other HOG-related genes such as *hog1* (logFC = −6.31), *ste20* (logFC = −10.28), and *ssk2* (logFC = −3.81) were downregulated (Figures 4A,B). Interestingly, the hybrid histidine kinase gene *sln1* with a putative role in osmosensing kept its transcriptional levels at both optimal and saturated NaCl concentrations. Although *ssk1* gene encoding a phosphorelay response regulator, recognized as an activator of Hog1 MAPK, was upregulated at saturated NaCl concentration, the MAPKKK gene *ssk2* was turned off resulting in a downregulation of *hog1* gene.

Saturated NaCl concentration also induces major changes in the transcriptional levels of genes related to cell wall ultrastructure and morphology (Gunde-Cimerman et al., 2018). In *A. sydowii* EXF-12860 grown at saturated NaCl concentration genes related to the synthesis of chitin, mannose, and cell wall proteins were downregulated, while *gas1* coding for glucanosyltransferase-domain-containing protein and *fks1* gene coding for β-1,3 glucan synthase, involved in the biosynthesis of β-1,3 glucans, were upregulated (Figure 4A). MAPKs related to cell wall damage response pathway, except for *sac7* gene encoding a GTPase-activating protein of Rho1 (Cid et al., 1998), were downregulated in *A. sydowii* EXF-12860 at saturated NaCl concentration (Figures 4A,B). Interestingly, genes encoding hydrophobins were not differentially expressed in *A. sydowii* exposed to saturated NaCl concentration. According to the transcriptional profile observed in *A. sydowii* EXF-12860, we propose two types of cell wall adaptations, one related to 1 M and the other to saturated NaCl concentration (Figure 4C). We hypothesize that the cell wall at saturated NaCl concentration contains a higher amount of β-1, 3 glucans with increased porosity and less rigidity due to repressed chitin biosynthesis. This hypothesis should be further evaluated by high-resolution nuclear magnetic resonance.

Additional differentially expressed genes at saturated NaCl concentration were related to starvation and pheromone synthesis (Figure 4B), such as the upregulated (logFC = 4.19) *rsp5* gene coding for E3 ubiquitin-protein ligase required for mating triggered by pheromones (Wang et al., 1999). Typically, this mating signal binds to a Ste (i.e., Ste2/Ste3) cell surface receptor, which in turn activates a MAP kinase cascade resulting in the expression of mating promoting genes (Zhu et al., 2011). Genes *cla4* and *ste20* coding for kinases involved in pheromone response (Melanie et al., 2007) was differentially expressed in *A. sydowii* exposed to saturated NaCl concentration (logFC = 4.15 and logFC = −10.28, respectively). In pheromone response the product of *bem1* gene, which was downregulated (logFC = −5.53), is necessary for the activation of Cdc42 into juxtaposition with Ste20. Since this step is critical for triggering the pheromone fungal response (Alvaro and Thorner, 2016), it seems that extreme NaCl conditions decrease the mating in *A. sydowii*.

At high NaCl concentrations fungal cells are exposed to toxic levels of sodium ions (Na^+^). To maintain transmembrane ionic homeostasis, membrane potential, intracellular pH, and other related physiological processes, they must vary transmembrane ion transporters (Ariño et al., 2010). In *A. sydowii* EXF-12860 the transcription of genes coding for transporters that facilitate the influx of glucose, amino acids, and Na^+^ was upregulated (Figures 4A,D). In *A. sydowii* at saturated NaCl concentration the transcription of the glycerol/H^+^ symporter STL1 that regulates the intracellular glycerol levels was upregulated (logFC = 4.76). Moreover, eight and two genes encoding ABC transporters were downregulated (−10,33 < logFC <−3.86) </logFC < −3.86)> and upregulated (logFC = 3,82; logFC = 4,04) at 5.13 M NaCl, respectively. These transporters are mainly related to the xenobiotic transporter activity and iron–sulfur transport across the cytoplasmic membrane.

In *A. sydowii* at saturated NaCl several Ca^2+^ transporters were downregulated (Figure 4A) and consequentially polarized growth was drastically affected (Figure 4E). This was further supported by the transcriptional repression of the *bem1* gene (logFC = −5.53) required for fungal cell polarity (Leberer et al., 1996). Other genes related to morphogenesis, cytokinesis, polarized growth, and the formation of germ tubes, such as *boi2* (logFC = −11.57), *rgd1* (logFC = −11.13), *myo5* (logFC = −9.71), and *dync1* (logFC = −6.0), were also downregulated (Figure 4A).

At saturated NaCl concentration genes involved in the cell cycle were downregulated (Figures 4A,F) as well providing strong evidence of the influence of extremely high NaCl concentrations on cell growth and increased energy requirements to maintain metabolic fluxes and physiological processes in *A. sydowii* at this extreme condition. In *A. sydowii* exposed to hyperosmolarity (5.13 M NaCl) cell cycle progression is arrested in the S phase, but mainly in the G2 phase and G2/M transition (Figures 4A,F). It has been suggested that delaying in the S phase during extreme water deprivation prevents DNA replication to avoid interferences with the transcription of genes involved in osmoadaptative responses. The transition from S into G2 phase is mainly governed by mitotic cyclins such as Clb1 and Clb2. Later, the cell-cycle transition from G2 into M phase is controlled by the morphogenetic checkpoint (Saito and Posas, 2012). In *A. sydowii* exposed to 5.13 M NaCl we found that *clb2* and different genes involved in the morphogenetic checkpoint were downregulated (Figures 4A,F).

### Importance of lncRNA and Transcriptional Factors in the Transcriptome of *Aspergillus sydowii* at Saturated NaCl Concentration

**Figure 5:**
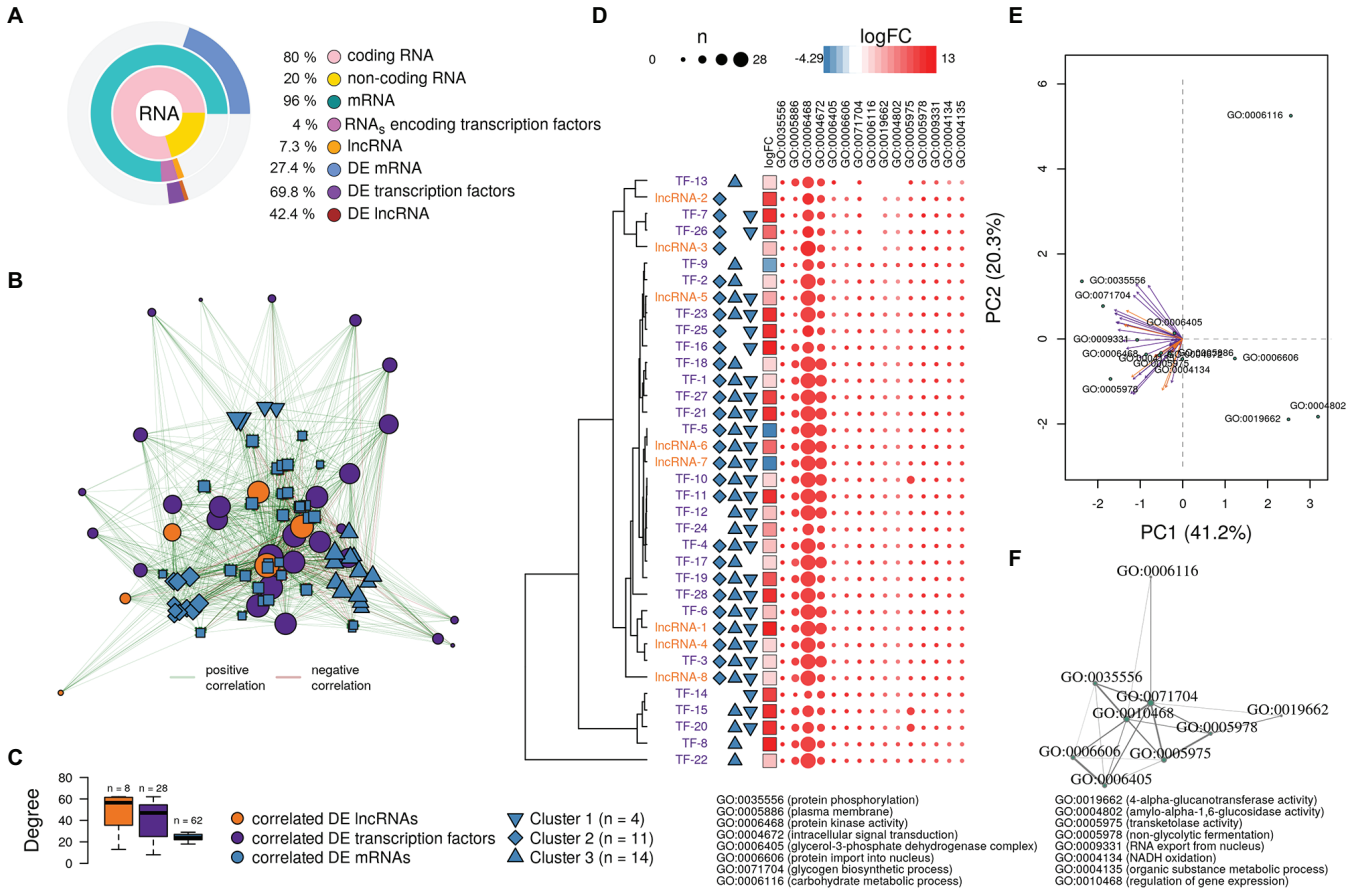
Analysis of the putative function of long non-coding RNAs (lncRNAs) and transcription factors found in the *A. sydowii* transcriptome (condition of 5.13 M NaCl compared to 1 M NaCl). **(A)** Relative amounts of differentially expressed (DE) mRNAs, lncRNAs, and RNAs encoding transcription factors (TFs) found in the *A. sydowii* transcriptome. **(B)** Topology of the directed network built with the most relevant DE lncRNAs and RNAs encoding TFs and their co-expressed DE mRNAs. **(C)** Distribution degree of DE lncRNAs and RNAs encoding TFs and their co-expressed DE mRNAs observed in the directed network. **(D)** LogFC of the selected lncRNAs and RNAs encoding TFs, their association with the identified clusters of DE mRNAs, and a heatmap of the counts of DE mRNAs that each regulatory molecule was correlated per GO term of interest. **(E)** Canonical correspondence analysis of the selected lncRNAs and RNAs encoding TFs and the GO terms related to biological functions relevant to overcome extreme osmotic stress. **(F)** Semantic similarity analysis of the GO terms annotated to DE mRNA co-expressed with DE lncRNAs and RNAs encoding TFs.

We found that ~80% and ~20% of transcripts were classified as coding and non-coding RNA, respectively (Figure 5A). Further, RNAs encoding transcription factors (TFs) represented 4% of the coding RNA and 7.3% of the non-coding RNA was classified as lncRNAs. The remaining non-coding transcripts were classified as tRNAs, snRNAs, and sRNAs, among others (Figure 5A). Despite their relatively low abundance, a great proportion of lncRNAs and RNAs encoding TFs were differentially expressed: 42.4% and 69.8% of the total lncRNAs and RNAs encoding TFs, respectively.

Previously it has been reported that the trans-role of lncRNAs is related to their co-expressed genes (Zhan et al., 2016). Gene expression is regulated both by TFs and lncRNAs. To identify possible gene regulatory modules playing a role in response to salinity, we derived an mRNA-TF-lncRNA network from the Pearson correlation coefficient matrix of differentially expressed transcripts (*r* > 0.95, value of *p* < 0.05; Jiang et al., 2016). The differentially expressed mRNAs evaluated are associated with GO terms with key roles in osmotic tolerance (i.e., protein phosphorylation, protein kinase activity, intracellular signal transduction, glycerol-3-phosphate dehydrogenase complex, and plasma membrane composition, among others). Fifteen (93.8%) of the differentially expressed lncRNAs and 62 (77.5%) of the differentially expressed RNAs encoding TFs were strongly correlated (*r* > 0.9, value of *p* < 0.05) to the 54.4% of the differentially expressed mRNAs (*n* = 309).

To analyze the correlation of the co-expression levels between the transcriptional regulators (lncRNAs and TFs) and their putative targets (regulated elements), we constructed a directed interaction network that included the differentially expressed lncRNAs and RNAs coding TFs that showed the highest co-expression levels (*r* > 0.95, value of *p* < 0.05) to mRNAs associated with GO terms relevant for osmotic stress (Figure 5B). The distribution degree (number of edges—connections—per vertices), shown in Figure 5C, indicates that lncRNAs and TFs displayed higher number of connections than mRNAs. The network reflects a hierarchical interactome where a regulatory element [i.e., lncRNAs (*n* = 8) or TFs (*n* = 28)] acts on several targets [mRNA (*n* = 62)]. Each differentially expressed lncRNA was related on average to ~48 differentially expressed mRNAs, while each RNA coding a TF was related on average to ~41 differentially expressed mRNAs. Interestingly, the differentially expressed mRNAs were related on average to only ~24 transcriptional regulators (i.e., lncRNAs and TFs), suggesting that the expression of each gene could be under the influence of both lncRNAs and TFs.

The network topology (Figure 5B) shows different clusters formed by differentially expressed mRNAs with a similar connection (edges) profile with differentially expressed lncRNAs and RNAs coding TFs. We identified three specific, distinctly separated clusters of differentially expressed mRNAs (Figure 5C). Their deeper analysis indicated that mRNAs grouped in cluster 1 (*n* = 4) were associated with GO terms related to metabolic processes (GO:0071704). Cluster 2 (*n* = 11) and cluster 3 (*n* = 14) were mRNAs associated with GO terms related to protein phosphorylation processes (GO:0006468) and protein kinase activity (GO:0004672), and plasma membrane composition (GO:0005886), respectively. In this analysis, “*n*” refers to the number of mRNAs in each cluster.

We used the correlation descriptors of the co-expression network (Figure 5B) to select the lncRNAs and RNAs encoding TFs that showed the highest Pearson correlations and the highest number of connections to differentially expressed mRNAs related to GOs relevant to hypersaline stress. Figure 5D shows the logFC of lncRNAs and RNAs encoding TFs correlated with the transcriptional level of the differentially expressed mRNAs grouped into the three identified mRNA clusters, but also the number of differentially expressed mRNAs that each regulatory molecule was correlated per GO term. It seems that almost all the RNAs encoding TFs and lncRNAs were correlated to at least one mRNA annotated into each GO term analyzed. Moreover, many of the RNAs encoding TFs and lncRNAs were correlated with at least two of the gene clusters identified in Figure 5B. Overall, lncRNAs and TFs identified could regulate the expression of mRNA associated with phosphorylation processes (GO:0006468), protein kinase activity (GO:0004672), intracellular signal transduction (GO:0006468), glycerol-3-phosphate dehydrogenase complex (GO:0006405), and plasma membrane composition (GO:0005886), which are pivotal biological functions needed to overcome hypersaline stress and were recognized as such in the halophilic basidiomycetous *W. ichthyophaga* and extremotolerant black yeast *H. werneckii* (Zajc et al., 2013; Plemenitaš et al., 2014; Gunde-Cimerman et al., 2018). Interestingly, some lncRNAs (i.e., lncRNA-2 and lncRNA-3) appear to have a regulating role over mRNAs grouped only into cluster 2 (Figure 5D), which encode proteins involved in phosphorylation processes and protein kinase activity. Other lncRNAs (i.e., lncRNA-6 and lncRNA-7) seem to have an antagonist role during the transcription of the same clusters of mRNAs. This observation was confirmed by a canonical correspondence analysis (Figure 5E) that showed that GO:0006116 (carbohydrate metabolic process), GO:0006606 (protein import into nucleus), GO:0004802 (amylo-α-1,6-glucosidase activity), and GO:0019662 (4-α-glucanotransferase activity) have an inverse association to the expression of some of the lncRNAs and RNAs encoding TFs found in this analysis.

Finally, we conducted a semantic similarity analysis (Yu et al., 2010) to produce a GO term-based network to visualize the correlation profile between GO terms associated with the mRNAs correlated with lncRNAs and TFs (Figure 5F). The network highlights the role of both regulatory elements in the transcriptional profiles of genes involved in relevant biological responses related to osmotic stress such as carbohydrates metabolism, particularly glycogen biosynthesis and transketolase activity, protein phosphorylation, glycerol-3-phosphate dehydrogenase complex, and protein kinase activity.

### Changed Metabolomic Profile at Saturated NaCl Solution in Comparison With 1 M NaCl

**Figure 6:**
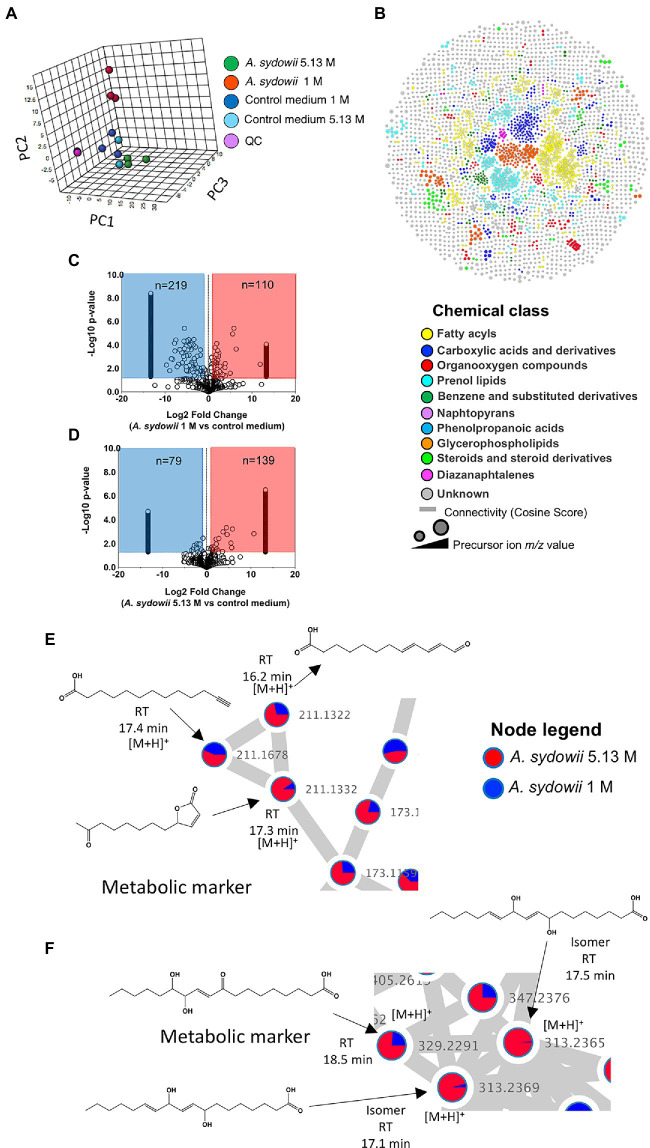
Untargeted metabolomics and chemoinformatic analyses of *A. sydowii* EXF-12860 grown with either low 1 M NaCl or high 5.13 M NaCl concentrations. **(A)** 3D principal component analysis (PCA) displaying components 1 and 2 of features (abundance normalized) detected in 1 M and 5.13 M NaCl concentrations, including appropriate media controls. Quality control (QC) samples are displayed to assure the method reliability and stability. **(B)** Molecular classes (retrieved by Classyfire) of the metabolome identified by the MolNetEnhancer workflow and visualized using Cytoscape version 3.8.2. Each node represents a unique feature, and the color of the node denotes the associated chemical class. The thickness of the edge (connectivity) indicates the MS^2^ similarity (Cosine score) among features. The *m/z* value of the feature is shown inside the node and is proportional to the size of the node. **(C)** Volcano plot showing the quantified features among 1 M NaCl media supernatant with and without *A. sydowii*. **(D)** Volcano plot showing the quantified features among 5.13 M NaCl media supernatant with and without *A. sydowii*. Each point represents a feature with assigned charge and isotope cluster; otherwise, it was excluded from the analysis. Molecular subnetworks 1 **(E)** and 2 **(F)** of the metabolites contributing to the separation between samples driven by the analysis of the principal component variable grouping (PCVG) loading plots. Each node represents a precursor ion (MS^1^), and the edge between nodes indicates similarity in MS^2^ fragmentation patterns using a cosine score of at least 0.6. The metabolites abundance is denoted by the colored area of the node, and the precursor ion mass is shown next to the node. The structures were derived from SIRIUS 4 and CSI:FingerID prediction.

The effects of salinity on the growth of *A. sydowii* at the metabolome level were evaluated by PCA (Figure 6A). *Aspergillus sydowii* grown at optimal and saturated NaCl concentration clustered separately, potentially indicating differences in the consumption of media nutrients and production of secondary metabolites. The tight clustering of quality control samples in the 3D plot (Figure 6A) assured the method reliability and stability, suggesting in turn that the differences in the metabolic profile as indicated by PCA are of biological origin and not due to technical variability. Our methodological pipeline allowed us to detect and identify various chemical classes of metabolites in the supernatants of *A. sydowii* grown in both saline conditions, ranging from fatty acyls to diazanaphtalenes in the top 10 (Figure 6B). To further mine the complex data and identify differences among the metabolome of *A. sydowii* grown at optimal and at saturated NaCl concentrations, we compared the features abundance in both salt conditions using their respective media control (blank media containing 1 M or 5.13 M NaCl and nutrients; Figures 6C,D). This enabled detection of changes in the metabolism (downregulated or upregulated) of media components or nutrients and to identify secondary metabolites linked specifically to hypersaline environment. We quantified 1,137 and 1,090 features at 1 M and 5.13 M NaCl, respectively.

There were more downregulated features in the 1 M vs. 5.13 M NaCl supernatant, probably related to *A. sydowii* thriving and better metabolizing nutrients in the media at optimal (1 M) NaCl concentration (Figures 6C,D). To identify or annotate the potential metabolites used by the fungus as energy source, we utilized the GNPS platform and SIRIUS/CSI:FingerID (Dührkop et al., 2015). We observed downregulation of various unsaturated C18 fatty acids only in supernatants obtained at 1 M NaCl, but not at saturated NaCl concentration (Table 1). In contrast, two aromatic metabolites (i.e., benzoic acid ester and phenyl-containing alanine derivative) completely vanished from the media with saturated NaCl concentration after being exposed to *A. sydowii* (Table 1).

**Table 1:** List of putatively annotated metabolites with increased and reduced abundance in low and high salt conditions.

Condition (NaCl)	Chemical class^a^	Compound name	Retention time (s)	Experimental mass [adduct]	Exact mass	Mass error (ppm)	Fold change^b^	Putative structure^c^
1.0 M	Fatty acyls	9-Oxo-10E,12Z-octadecadienoic acid	20.3	295.2262 [M + H]^+^	295.2266	−1.3	0.02	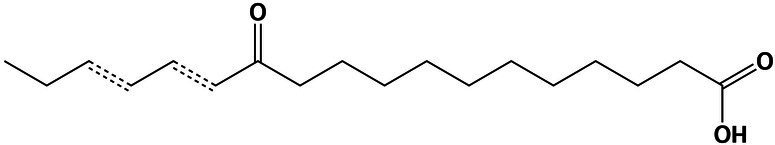
			18.5	295.2253 [M + H]^+^	295.2266	−4.4	0.03	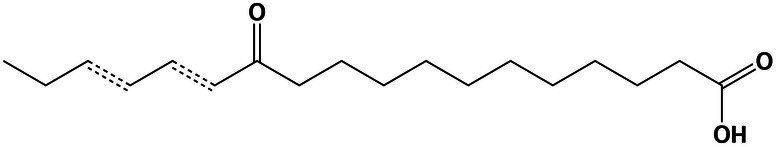
			16.4	295.2268 [M + H]^+^	295.2266	0.6	0.06	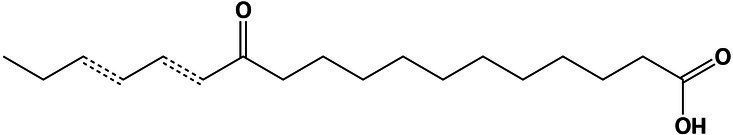
		12(13)-Epoxy-9Z-octadecenoic acid	19.09	297.2423 [M + H]^+^	297.2423	0	0.03	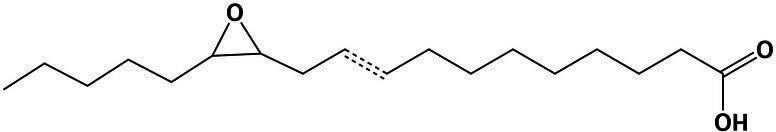
		12,13-Dihydroxy-9Z-octadecenoic acid	19.09	315.2531 [M + H]^+^	315.2529	0.6	0.03	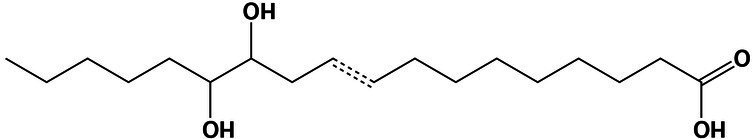
		13S-Hydroxy-9Z,11E,15Z-octadecatrienoic acid	16.4	277.216 [M + H-H_2_O]^+^	277.2161	−0.3	0.09	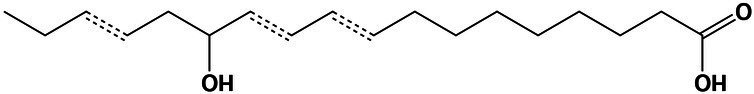
	Unclassified	(*Z*)-*N*-(2-([1,1′-biphenyl] -2-yloxy)ethyl)-4-phenyltetrahydro-2*H*-pyran-4-carbimidic acid	22.01	402.2013 [M + H]^+^	402.2062	−12.1	0.2	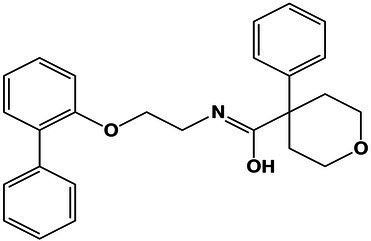
	Steroids and steroids derivatives	5-Alpha-androsterone	16.6	273.2207 [M + H-H_2_O]^+^	273.2219	4.3	1.7	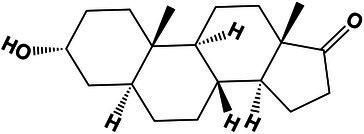
		17-Hydroxy-10,13-dimethyl-2,3,6,7,8,9,10,11,12,13,14,15,16,17-tetradecahydro-1*H*-cyclopenta[*a*]phenanthren-1-one	17.1	289.2153 [M + H]^+^	289.2168	−5.1	2.2	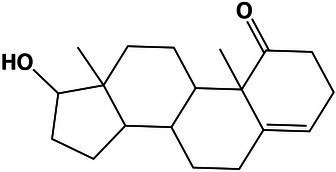
	Prenol lipids	1-Phenanthrenecarboxylic acid, 1,2,3,4,4a,9,10,10a-octahydro-9-hydroxy-1,4a-dimethyl-7-(1-methylethyl)-, (1S,9R)-	19.8	299.2005 [M + H-H_2_O]^+^	299.2004	−2.3	4.8	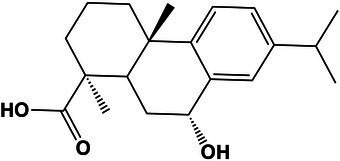
	Phenols	N-Acetyl tyramine	10.8	180.1012 [M + H]^+^	180.1018	−3.3	5.1	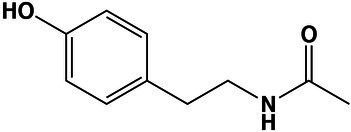
	Glycerolipids	Monolaurin	20.5	257.2082 [M + H-H_2_O]^+^	257.2118	−13.9	5.9	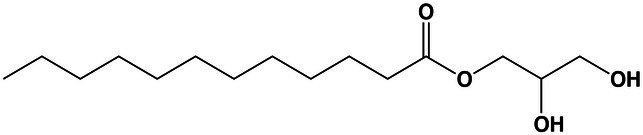
1.0 M	Unclassified	(*E*)-2-((7-((1-Hydroxybutan-2-yl)amino)-3-isopropyl-2,4-dihydro-5*H*-pyrazolo[4,3-*d*]pyrimidin-5-ylidene)amino)butan-1-ol	19.1	337.2348 [M + H]^+^	337.2345	0.8	<1,000	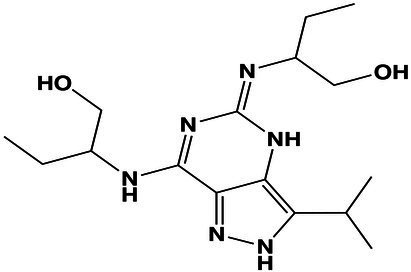
	Unclassified	(*Z*)-16-((2-Ethoxyethyl)imino)-16-hydroxyhexadecanoic acid	20.3	358.2944 [M + H]^+^	358.2951	−1.9	<1,000	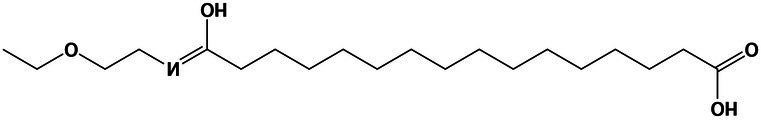
	Benzene and substituted derivatives	(*E*)-Prop-1-en-1-yl benzoate	21.9	163.0745 [M + H]^+^	163.0752	−4.2	<1,000	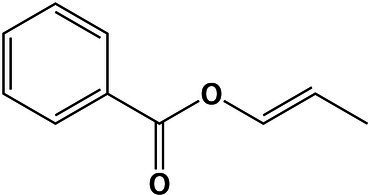
5.13 M	Steroids and steroids derivatives	Cholic acid	16.1	391.2816 [M + H-H_2_O]^+^	391.2849	−8.4	2.8	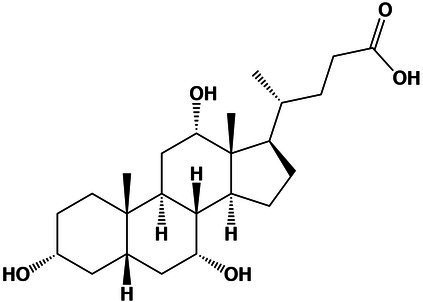
	Carboxylic acids and derivatives	Val-Phe	10.8	265.1524 [M + H]^+^	265.1552	−10.5	3.1	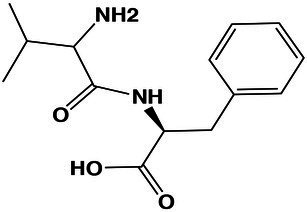
	Benzene and substituted derivatives	(*E*)-Prop-1-en-1-yl benzoate	22.03	163.0746 [M + H]^+^	163.0752	−3.6	<1,000	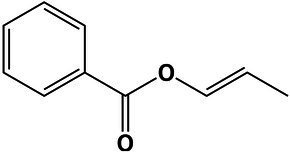
	Unclassified	(2-Ethylphenyl)alanine	19.1	194.1161 [M + H]^+^	194.1174	−6.6	<1,000	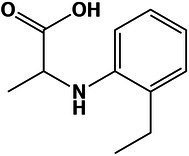

a Classification by Classyfire according to the structure predicted by SIRIUS 4.4 (when available) or spectral match against GNPS libraries.

b Fold change with respect to their respective medium control; 1 M NaCl/control or 5.13 M NaCl/control.

c Structure predicted by SIRIUS 4.4 (when available) or spectral match against GNPS public spectral libraries.

To further reduce the complexity of the metabolomic analysis and focus on specific NaCl-related metabolites, we mined data using PCA and PCVG analyses (Supplementary Figure S2; Ivosev et al., 2008). Careful inspection of the grouping patterns revealed that components 1 and 2 were relevant for identification of downregulated features at optimal and saturated NaCl concentration. In contrast, components 3 and 4 allowed detection of features that dominated in *A. sydowii* grown at either optimal or saturated NaCl concentrations (denominated hereafter as “metabolic markers”; Figures 6D,E; Table 2). Noteworthy, more metabolites were detected at saturated NaCl concentration, and three among them were indicated as metabolic markers for this salt concentration. Their presence was confirmed by the extracted ion chromatograms and profile plots (intensity of the peak across biological replicates; Supplementary Figure S2).

**Table 2:** List of annotated metabolites denoted by PCVG as metabolic markers in low and high salt conditions.

Condition (NaCl)	Chemical class^a^	Compound name	Retention time (s)	Experimental mass [adduct]	Exact mass	Mass error (ppm)	Fold change^b^	Putative structure^c^
1.0 M	Unclassified	(2-Hydroxy-4-methylpentanoyl)leucine	15.7	246.1692 [M + H]^+^	246.1699	−2.8	1.6	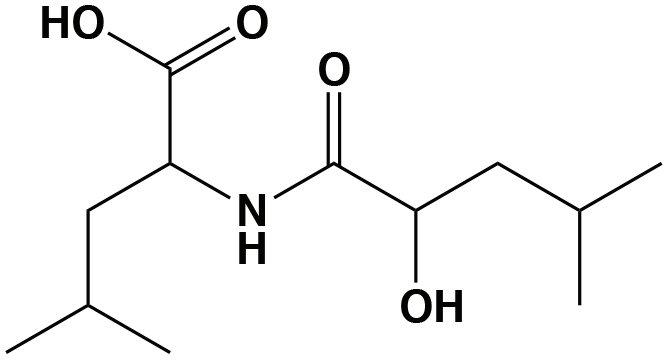
	Prenol lipids	17-Hydroxy-10,13-dimethyl-1-phenanthrenecarboxylic acid, 1,2,3,4,4a,9,10,10a-octahydro-9-hydroxy-1,4a-dimethyl-7-(1-methylethyl)-, (1S,9R)-	19.8	299.2005 [M + H-H_2_O]^+^	299.2004	0.3	3.4	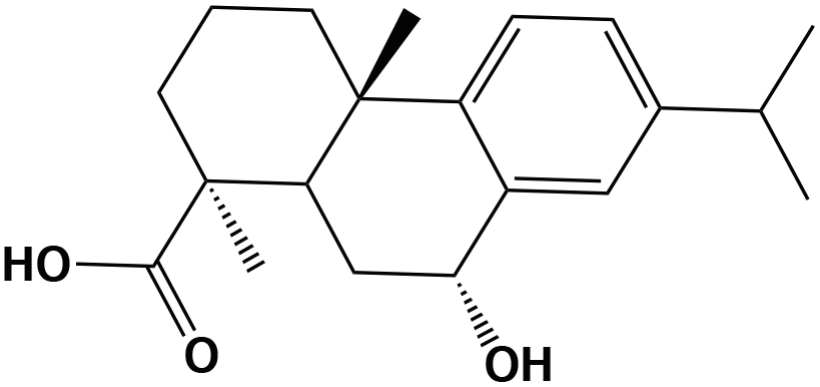
	Carboxylic acids and derivatives	4,4′-(Cyclohexane-1,2-diyl)dibutyric acid	15.1	257.1740 [M + H]^+^	257.1746	−2.3	3.6	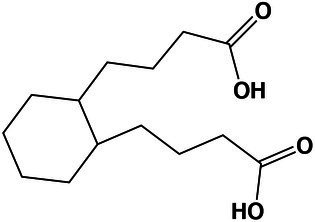
	Phenols	N-Acetyl tyramine	10.8	180.1012 [M + H]^+^	180.1018	−3.3	4.9	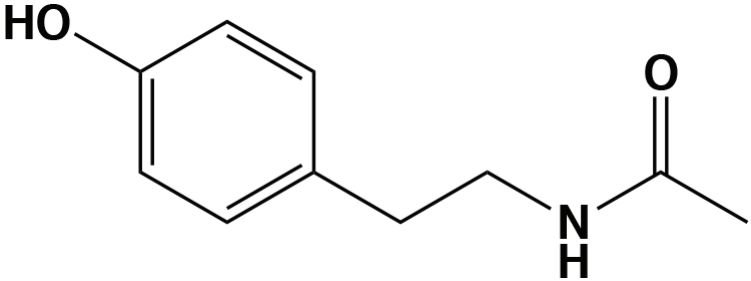
	Unclassified	Diethyl 3,3′-[butane-1,4-diylbis(azaneylylidene)](3*Z*,3′*E*)-dibutyrate	19.2	313.2175 [M + H]^+^	313.2121	17.2	>1,000	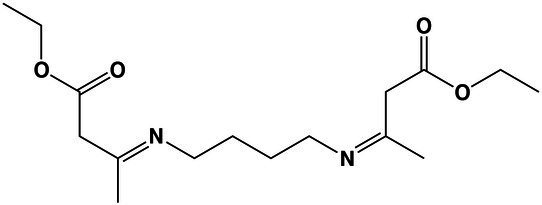
5.13 M	Dihydrofurans	2-(4-Oxo-5-pentylcyclopent-2-en-1-yl) acetic acid	17.3	211.1332 [M + H]^+^	211.1327	2.3	13.4	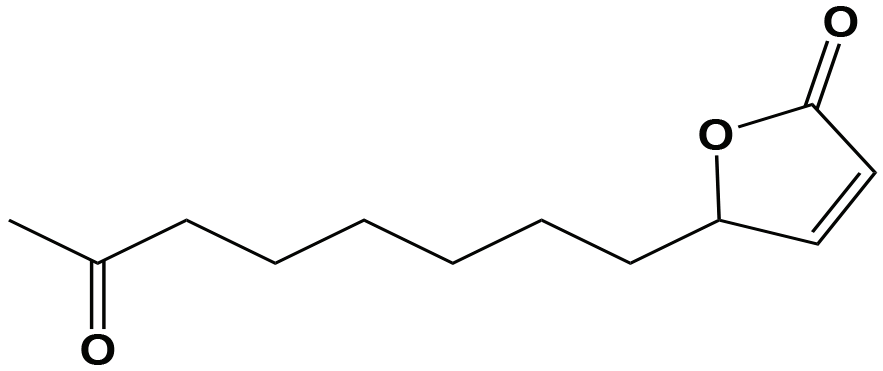
	Fatty acyls	(E)-12,13-Dihydroxy-9-oxooctadec-10-enoic acid^b^	18.5	329.2291 [M + H]^+^	329.2321	−9.1	14.3	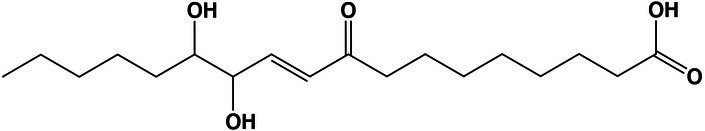
	Unclassified	1-(2-(Dimethylamino)ethyl)-2,2-dihydroxyhydrazine-1-carboximidamide	9.6	178.1316 [M + H]^+^	178.1297	10.6	>1,000	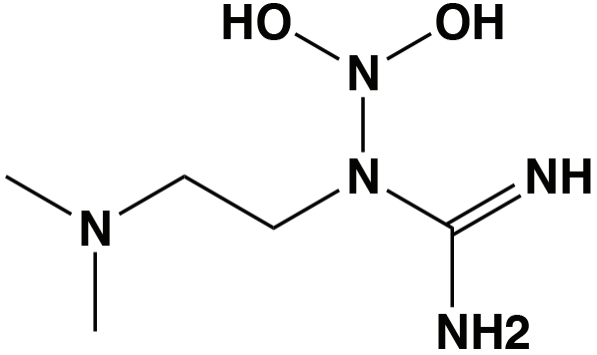

a Classification by Classyfire according to the structure predicted by SIRIUS 4.4 (when available) or spectral match against GNPS libraries.

b Fold change with respect to their respective medium control; 1 M NaCl/control or 5.13 M NaCl/control.

c Structure predicted by SRIUS 4.4 (when available) or spectral match against GNPS public spectral libraries.

Using molecular networks (MS^2^ similarity across all detected metabolites) retrieved by GNPS, we could propagate the recognition of the noted metabolic markers. Their putative chemical structures, retrieved by CSI:Finger ID, are shown in Table 2. We could annotate only a small fraction of the features that appeared at saturated NaCl concentration even with CSI:Finger ID, probably due to the complex and unknown structures of the metabolites not yet contained in libraries or databases. In Figure 6E we observed a cluster (subnetwork 1) of metabolites, structurally related to the PCVG-derived metabolic marker [M + H]^+^ 211.1332 *m/z*. The predicted chemical structures of these groups of metabolites were related to a butenolide derivative and two fatty acids (Figure 6E). Likewise, we noted a cluster (subnetwork 2) containing the metabolic marker 329.2291 [M + H]^+^ and two structurally related C18 hydroxylated polyunsaturated fatty acids (isomers) as predicted by SIRIUS and CSI:FingerID (Figure 6F). The metabolic markers found at optimal NaCl concentration were structurally more diverse; therefore, we could not identify subnetworks of structurally related metabolites. Nevertheless, two amino acid derivatives (N-acetyl tyramine and leucine-containing dipeptide) were noted in this group (Table 2).

## Discussion

Various studies investigated adaptations of halotolerant and halophilic fungi to saline and hypersaline conditions (Marjetka et al., 2010; Zajc et al., 2013; Liu et al., 2017; Gunde-Cimerman et al., 2018; Turk and Gostinčar, 2018; Ding et al., 2019; Tafer et al., 2019; Lee et al., 2021). However, only few investigated the fungal mechanisms occurring at saturated NaCl concentrations (≥5 M NaCl; Kogej et al., 2007; Kunčič et al., 2010; Zajc et al., 2013). This is the first study aimed at analyzing the mRNA profile of a halophilic ascomycetous filamentous fungus (*A. sydowii*) exposed to saturated concentration of NaCl (5.13 M).

The biological processes enriched in *A. sydowii* at 5.13 M NaCl (e.g., phosphorelay signal transduction system, RNA and protein traffic across nucleus, regulation of gene expression, regulation of macromolecules and primary metabolism, non-glycolytic fermentation, polysaccharide biosynthesis, cell wall β-glucan biosynthesis, polyol metabolism, ketone body catabolism, and NADH oxidation) reflect the needed extensive physiological reprogramming of the fungal cell at saturated NaCl concentration (Figure 1A). Some of these metabolic processes, including cell wall glycan and polysaccharide biosynthesis, phosphorylation signal transduction system, and polyol metabolism, were identified as crucial in the two most investigated model fungi for adaptations to hypersaline conditions, the obligately halophilic basidiomycetous *Wallemia ichthyophaga*, and the extremely halotolerant black yeast *Hortaea werneckii* (Kogej et al., 2007; Zajc et al., 2013; Gunde-Cimerman et al., 2018). Glycerol metabolism and regulation of primary metabolism were also enriched in the non-conventional halotolerant yeasts *Hyphopichia burtonii* and *Hyphopichia pseudoburtonii* when exposed to salt stress (Lee et al., 2021). The overrepresented biological functions of EXF-12860 at saturated NaCl concentration are also related to adaptations needed for growth at low temperatures, since both conditions induce low water activity. Recently described psychrophilic yeast *Rhodotorula frigidialcoholis*, when grown at 0°C and 23°C, has at 0°C more abundant transcripts related to carbohydrate metabolism, primary metabolism, signal transduction, and glycan biosynthesis (Touchette et al., 2021).

The GO multi-scale network analysis reveals a comprehensive picture showing informative GO signature clusters, indicating relevant biological processes and molecular functions related to hypersaline tolerance in *A. sydowii* EXF-12860: phosphorelay signal transduction, polysaccharide metabolism, and transferase activity (Figure 1D).

*Aspergillus sydowii* also reprogrammed the amino acid metabolism at saturated NaCl concentration (Figures 2A,B). The free amino acids composition revealed that glutamate showed higher amounts at both optimal and saturated NaCl concentrations, in both mycelium and supernatant (Figure 2B). Increased levels of negatively charged amino acids (i.e., glutamic acid and aspartic acid) have been recognized as a major adaptation of proteins synthetized by halophilic microorganisms including halophilic archaea (Abu-Seidah, 2007; Gunde-Cimerman et al., 2018; Tafer et al., 2019; Lee et al., 2021). The amino acid metabolism was also reprogrammed at transcriptional level being 33 genes differentially expressed (Figure 2C; Supplementary Table S3). Interestingly, the upregulation of genes related to the synthesis of histidine, tryptophan, glycine, cysteine, methionine, leucine, valine, and isoleucine was not accompanied by an increase in the amount of free amino acids. This was also shown in the halotolerant yeast *H. burtonii*, which overexpressed genes involved in amino acid anabolism without a positive correlation with the free amino acid levels (Lee et al., 2021).

Gene *hoga1* involved in the synthesis of pyruvate was upregulated at 5.13 M NaCl (Figure 2C). Pyruvate, a key metabolite of the intermediary carbon metabolism, is a crucial metabolic branch point for the biosynthesis of leucine, valine, isoleucine, and alanine (Bromke, 2013; Figure 2C). Pyruvate levels also regulate the Krebs cycle flux, in particular the synthesis of α-ketoglutarate and oxaloacetic acid, key intermediates involved in the formation of glutamate and aspartate, respectively (Huang et al., 2019). At the same time, aspartate is a branch node for the biosynthesis of lysine, asparagine, and threonine (Lunt et al., 2015; Walvekar and Laxman, 2019; Figure 2C). Thus, pyruvate contents do not only have important implications in the cell energetic balance, but small limitations in pyruvate availability could also alter the amino acid biosynthesis (Lunt et al., 2015). The observed increase in the level of *hoga1* transcripts suggests that pyruvate in *A. sydowii* grown at saturated NaCl concentration could represent an anabolic redirect node toward primary energetic metabolism (ATP biosynthesis) and biosynthesis of amino acids.

Genes involved in methionine (*mmuM*) and cysteine (*cth*) biosynthesis were also upregulated at saturated NaCl concentration (Figure 2C). Methionine is a sulfur-containing amino acid that regulates the initiation of translation, turns on anabolic pathways in fungi, and mediates several methyl-transferase metabolic reactions (Walvekar and Laxman, 2019). CTH enzymes have broad substrate specificity and transform cystathionine derivates from methionine into cysteine (Jurkowska et al., 2014). Cysteine residues play crucial roles in stabilizing the tridimensional structure of proteins by disulfide bridges, important in preserving the protein functions at denaturalizing conditions imposed by high NaCl concentrations. Also, cysteine has key roles in the redox balance homeostasis, biosynthesis of coenzyme A, iron–sulfur (Fe-S) cluster biogenesis, detoxification processes, and protein trafficking (Fani et al., 2007; Jurkowska et al., 2014). Genes encoding homoserine acetyltransferase (*metX*) and homoserine succinyltransferase (*metA*) were also overexpressed (Figure 2C). These homoserine transferases yield *O*-acetyl-*L*-homoserine and *O*-succinyl-*L*-homoserine, respectively, two anabolic intermediates that maintain the metabolic flux to synthesize both cysteine and methionine, respectively (Shrivastava et al., 2021). In *A. sydowii L*-homoserine seems to be a critical metabolic substrate, connected to both cysteine and methionine biosynthesis pathways. As gene *met17* that encodes *O*-acetylhomoserine sulfhydrylase was downregulated (logFC = −3.84; Figure 2C), the synthesis of methionine could occur probably *via O*-succinyl-*L*-homoserine. In brief, transcriptomic data suggest that direct sulfhydrylation reaction is not the main pathway to produce homocysteine, a direct precursor for methionine biosynthesis.

The upregulated gene *hisF/H* mediates the central metabolic reaction of the histidine biosynthesis pathway due to synthesis of the imidazole ring of the histidine precursor (IGP; Fani et al., 2007). In this context *egtD* gene was downregulated (logFC = −4.72), since its product *L*-histidine tri-methyltransferase catalyzes the consumption of histidine to form hercynine, probably resulting in a decrease of the available histidine levels. The transcriptional activation of histidine biosynthetic metabolism at extremely low water activity due to saturated NaCl concentration could be related to the role of histidine in membrane stress sensors histidine kinases (Marin et al., 2003), one of the molecular function GO terms enriched at saturated NaCl concentration. Since several histidine kinases respond to both osmotic stress and cold stress (Mikami et al., 2002), upregulation of the histidine biosynthesis pathway has been observed as well in *R. frigidialcoholis* and *Mrakia blollopsis* during growth at zero and subzero temperatures, respectively (Tsuji, 2016; Touchette et al., 2021). Histidine levels also increased in the halotolerant yeast *H. burtonii* at osmotically stressed conditions, although no differential transcriptomic response was observed (Lee et al., 2021). Additionally, it showed an overexpressed transcriptomic profile related to the synthesis of leucine and methionine (Lee et al., 2021), as shown in *A. sydowii* exposed to saturated NaCl concentration (Figure 2C). This study demonstrates that extreme water deprivation by saturated NaCl concentration imposes a considerable transcriptional reprogramming, resulting in altered amino acid biosynthesis pathways.

Hypersaline conditions also cause in halophilic fungi the turnover of the synthesis of unique fatty acids, increase the concentration of free fatty acids trigger modifications in the fatty acid chain elongation, and increase overall unsaturation (Gunde-Cimerman et al., 2018). All these modifications influence the plasma membrane composition and/or fluidity, as it has been reported for several halophilic/halotolerant microorganisms, such as the alga *Dunaliella salina* (Katz et al., 2007), the protist *Halocafeteria seosinensis* (Harding et al., 2017), and fungi *A. pullulans*, *H. werneckii*, and *W. ichthyophaga* (Gunde-Cimerman et al., 2018).

The C18 monounsaturated and polyunsaturated fatty acid profile in *A. sydowii* at both optimal and saturated NaCl concentrations (Figure 3A) is in agreement with results obtained for the alga *D. salina* (Katz et al., 2007), black yeast-like fungi *A. pullulans*, *H. werneckii*, and *Phaeotheca triangularis*, with a higher ratio of mostly unsaturated C18 fatty acids at hypersaline conditions (Turk et al., 2004). Although 10%–17% NaCl concentrations induced an increase in the fatty acid unsaturation in *A. pullulans* and *H. werneckii*, higher concentrations (25% NaCl) did not increase the unsaturation level in extremely halotolerant *H. werneckii* (Turk et al., 2004), in agreement with the fatty acid unsaturation profile in *A. sydowii* at NaCl saturation.

Transcriptomic analysis in *A. sydowii* exposed to 5.13 M NaCl showed that several anabolic and catabolic pathways related to the fatty acid metabolism were turned off transcriptionally (Figure 3B; Supplementary Table S4). Only the genes *fabG*, *mgll*, and *cdipt* were found to be overexpressed in the transcriptome. Gene *fabG* product that catalyzes an NADPH-dependent reduction of 3-ketoacyl-ACP to the (*R*)-3-hydroxyacyl-CoA substrates is essential for type II fatty acid biosynthesis (Nomura et al., 2020). Probably in *A. sydowii* exposed to high NaCl concentration FabG enzyme mediates the formation of (*R*)-3-hydroxyacyl-CoA substrates that could be incorporated in the secondary metabolism to produce polymers stress-related polyesters (Nomura et al., 2020). Recently, an observed increase in the level of *fabG* transcripts in the filamentous fungus *Glarea lozoyensis* has been related to the synthesis of secondary metabolites such as pneumocandin, the precursor of the antifungal drug caspofungin (Zhang et al., 2020). Upregulation of *fabG* in *A. sydowii* exposed to saturated NaCl concentration might be correlated to the synthesis of different secondary metabolites or to the redirection of more acetyl-CoA to the fatty acid biosynthesis, resulting in the formation of lipids, since free fatty acid content did not increase at saturated NaCl concentration.

On the other hand, the product of the *mgll* gene plays a key role in the conversion of monoacyl-glycerol into glycerol, the most frequent and relevant compatible solute to compensate for hypersaline stress in halophilic fungi (Gunde-Cimerman et al., 2018). MGL are a unique class of lipases with very well-understood biological function in mammals and bacteria (Rengachari et al., 2013), but not in fungi. It is known that MGLs obtained from fungi *Aspergillus oryzae*, *Candida rugosa*, *Mucor miehei*, *Penicillium camembertii*, *Penicillium cyclopium*, *Stemphylium lycopersici*, and *Sordaria* sp. act on mono- and diacylglycerols, even on triacylglycerols (Yamaguchi and Mase, 1991; Toida et al., 1998; Benjamin and Pandey, 2001; Jermsuntiea et al., 2011; Rocha et al., 2020). In *A. sydowii* MGL could be involved in the conversion of high range of acylglycerols to maintain the high intracellular amounts of glycerol needed at saturated NaCl concentrations. The downregulation of *glpK* (logFC = −4.30) and *adh* (logFC = −3.86) genes could support this notion (Figure 3B). Finally, the third overexpressed gene (*cdipt*, logFC = 4.04) encodes the enzyme that catalyze the last step of the *de novo* biosynthesis of phosphatidylinositol (PtdIns), importantly involved in fatty acid metabolism, intracellular signal transduction, and energy metabolism in eukaryotes (Blunsom and Cockcroft, 2019), all key metabolic processes at low water activity induced by saturated NaCl concentration.

Our transcriptomic data are consistent with the results obtained for the halophilic protist *H. seosinensis* where the shorter fatty acid chains increased at hypersaline conditions (Harding et al., 2017). Transcriptional downregulation of fatty acid elongation observed in *A. sydowii* at saturated NaCl concentration was observed as well in the extremely halotolerant black yeast *H. werneckii* and filamentous *W. ichthyophaga* (Plemenitaš et al., 2008; Gunde-Cimerman et al., 2018).

The analysis of molecular adaptations at saturated NaCl also showed the importance of high-osmolarity glycerol (HOG) signal transduction pathway (Figure 4). HOG pathway mediates sensing and responding to osmotic stress in halophilic and halotolerant microorganisms, including fungi (Gunde-Cimerman et al., 2018; Plemenitaš, 2021). The transmembrane osmosensor gene *sho1* which plays a critical role in sensing water deprivation was transcriptionally overexpressed. Interestingly, while *ssk1* gene was upregulated, the MAPKKK gene *ssk2* was downregulated as well *hog1* gene. These results suggest that osmoresponsive genes could be temporally transcriptionally activated and that other factors could also play a key role in their transcriptional regulation (Kejžar et al., 2015).

As demonstrated for other halophilic fungi (Gunde-Cimerman et al., 2018), saturated NaCl concentration also induced major changes in the transcriptional levels of genes related to cell wall ultrastructure and morphology in *A. sydowii*. MAPKs genes involved in the cell wall damage response pathway were downregulated. This set of MAPKs (i.e., Sac7, Rho1, Stt4, Bck1, Mkk1/2, Bni1, Rom1/2, etc.) mediates the fungal ability to switch between different cell wall structuring in response to saline stress (Levin, 2005), crucial to regain cell wall rigidity, porosity, and permeability in halophilic fungi (Gunde-Cimerman et al., 2018). In contrast to that observed in *A. sydowii* grown at 0.5 M and 2 M NaCl (Pérez-Llano et al., 2020), hydrophobins were not found to be differentially expressed at 5.13 M NaCl. Hydrophobins are cell wall-associated amphipathic proteins, which were transcriptionally upregulated in the obligate halophilic fungus *W. ichthyophaga*, indicating their important role in the adaptation of cell wall to salinity (Turk and Gostinčar, 2018).

In addition, genes related to starvation and pheromone biosynthesis were differentially expressed in *A. sydowii* exposed to extreme water deprivation by salt (5.13 M NaCl). Our transcriptomic results suggest that saturated NaCl concentration decreases the mating in *A. sydowii*. It needs to be added that the crosstalk between fungal pheromone signaling and high NaCl concentrations is still poorly understood.

As observed in other halophilic fungi, *A. sydowii* varied the transmembrane ion transporter repertory at saturated NaCl concentrations (Gunde-Cimerman et al., 2018). Our transcriptomic analysis is in agreement with the observation for the obligate halophile *W. ichthyophaga*, which increased the transcriptional rate of several Na^+^-exporting Ena ATPs to preserve the intracellular K^+^/Na^+^ ratio and increased the uptake of glucose to maintain metabolic fluxes at limiting NaCl concentrations (Zajc et al., 2013). *A. sydowii* also upregulated the expression of the glycerol/H^+^ symporter STL1 involved in the maintaining of the intracellular glycerol levels. Similar results were observed in *Saccharomyces cerevisiae* exposed to hyperosmotic shock and to other fungi grown at hypersaline conditions (Kogej et al., 2007; Zajc et al., 2013).

Cell cycle was also severely influenced by saturated NaCl concentration. Cell cycle arrest is a well-documented strategy in yeasts and filamentous fungi to contend with hyperosmolarity (Solé et al., 2014). In the presence of different stimuli (i.e., osmostress), cells must delay the cell cycle progression to allow the adaptive responses to the new environmental conditions before cells trigger vulnerable cell-cycle transition periods. In yeasts such as *S. cerevisiae* and *Schizosaccharomyces pombe*, water deprivation induces the phosphorylation of Hog1 kinase, which is involved in regulating all phases of the cell cycle and a rapid cell cycle arrest is observed (Saito and Posas, 2012). Our results suggest that mitogenic checkpoint is sensitive to saturated NaCl concentrations (Saito and Posas, 2012).

Our data also revealed the importance of lncRNAs and RNAs encoding TFs in the transcriptome of *A. sydowii* at hypersalinity (5.13 M NaCl). While the 42.4% and 69.8% of the total lncRNAs and RNAs encoding TFs were differentially expressed, respectively, only the 27.4% of the mRNAs was differentially expressed. These results reflect the key role that lncRNAs and TFs might play in the transcriptional response of *A. sydowii* exposed to extremely saline conditions. This study, which represents the first attempt to clarify the role of lncRNAs in response to high NaCl stress in halophilic fungi, opened a new perspective to elucidate the biological functions of lncRNAs in the regulation of gene expression, as exemplified by a great number of lncRNAs that were upregulated at saturated NaCl concentration.

Although molecular adaptations have been studied in some halophilic and halotolerant fungi (Zajc et al., 2013; Liu et al., 2017; Gunde-Cimerman et al., 2018; Turk and Gostinčar, 2018; Ding et al., 2019; Tafer et al., 2019; Lee et al., 2021), so far no high-throughput metabolomics studies were performed to show the potential metabolic reprogramming at high NaCl concentrations. This study is the first attempt at obtaining a comprehensive metabolomic picture in a halophilic fungus. The metabolomic profile showed that various unsaturated C18 fatty acids were downregulated exclusively in supernatants obtained at 1 M NaCl (Table 1). Likewise, the higher abundance of unsaturated C18 fatty acids was observed with *A. pullulans*, *D. salina*, *H. werneckii*, and *P. triangularis*, when grown under at hypersaline conditions (Turk et al., 2004; Katz et al., 2007; Gunde-Cimerman et al., 2018), and may be attributed to a lower degradation of such compounds. At the same time the aromatic metabolites benzoic acid ester and phenyl-containing alanine derivative were consumed at 5.13 M NaCl reflecting a metabolic switch of the fungus toward non-lipid sources at conditions of NaCl-induced extremely low water activity. An increase in energy production is one of the fundamental adaptations that maintain ion homeostasis and osmotic equilibrium in a hyperosmotic environment (Gostinčar et al., 2011). Priority is given to processes that are essential for cell survival. This metabolic switch we mention seems to allow the fungus to allocate mainly its metabolic energy to ensure processes with high carbohydrate demand such as the biosynthesis of the cell wall and compatible solutes (glycerol, mannitol, arabitol, erythritol, and trehalose). In both, glucose is the starting compound for these synthetic processes. Cell wall thickening by increased β-1,3-glucan and synthesis of compatible solutes are two of the main adaptations to halophily reported for fungi, specifically in the genus *Aspergillus*: *A. niger* (Diano et al., 2006), *A. nidulans* (Mansour, 2017), *A. oryzae* (Ruijter et al., 2004), *A. sydowii* (Pérez-Llano et al., 2020; Rodríguez-Pupo et al., 2021) and also reported in this work. This shift to non-lipid sources may also be related to the maintenance of appropriate cytoplasmic membrane fluidity, an important factor for the indispensable functioning of transporter families that are activated in response to osmotic changes in halophilic and halotolerant fungi (Turk et al., 2004).

Interestingly, some metabolites showed massive fold changes (i.e., <1,000; Tables 1 and 2). For example, (*E*)-prop-1-en-1-yl benzoate, an intermediate in the synthesis of kasanosin C, showed a decrease by more than 10-fold at both NaCl concentrations (1 M and 5.13 M) compared to control cultures obtained in the absence of salt (Table 1). Kasanosin C is an azaphilone, a structurally diverse class of fungal polyketide pigments with affinity for ammonia and amines (Shao et al., 2020). Although polyketide pigments are involved in the resistance to different environmental perturbations, it has been observed that salinity and low temperatures drastically affect the pigment production in fungi such as *Talaromyces albobiverticillius* (Venkatachalam et al., 2019) and *Monascus* sp. (Ahn et al., 2006), respectively. Our results also suggested that in *A. sydowii* high salinity could negatively regulate the production of polyketide pigments (i.e., kasanosin C). These metabolomic findings may be the starting point for designing selective experiments to get a comprehensive understanding about the role of certain metabolites during the fungal growth in the presence of salt stress (Venkatachalam et al., 2019).

## Conclusion

Based on our previous studies (Jiménez-Gómez et al., 2020; Pérez-Llano et al., 2020; Rodríguez-Pupo et al., 2021), *A. sydowii* is recognized as a model organism to study the adaptations of filamentous fungi to hypersaline conditions. *A. sydowii* grows optimally at 1 M NaCl and can grow within the full range from zero to saturated NaCl (5.13 M). In this study a multi-omics approach was used for the first time to study responses of *A. sydowii*, particularly during extremely growth-limited 5.13 M NaCl.

Analyzing the mRNA profile at saturated NaCl showed 1,842 genes significantly differentially expressed, of which 704 were overexpressed. As revealed by GO analysis, the enriched biological process reflected extensive physiological adaptation to high salt concentrations (5.13 M NaCl), mainly on metabolism and signal transduction. Processes identified previously in other halophilic fungi as crucial for adaptations to hypersaline conditions (Plemenitaš et al., 2008; Zajc et al., 2013), were restructuring of the cell wall, synthesis of compatible solutes, and phosphorylation of the signal transduction system. These adaptations are also crucial for the growth of fungi at low temperatures.

Major changes at the transcriptional level included the HOG signal transduction pathway, ion transporters, and cell wall ultrastructure and morphology. Interestingly, genes encoding chitin synthesis were repressed, exposing the important role on cell growth and increased energy requirements at saturated NaCl of β-1,3-glucans, Ca^2+^ transporters, and gene products related to polarized growth, morphogenesis, and the cell cycle.

This study also is the first attempt to clarify the role of lncRNAs in response to stress caused by high NaCl concentrations. A great proportion of lncRNAs were upregulated, and the correlation of co-expression levels between the transcriptional regulators (lncRNAs and TFs) and their putative targets associated with GO terms reflected the importance of metabolic processes, protein phosphorylation, protein kinase activity, and plasma membrane composition for the adaptation to high salt concentrations.

Changes at the level of transcriptional reprogramming of amino acid metabolism resulted in altered amino acid biosynthesis pathways, differences in amino acid profiles, and lower quantities of free amino acids. The essential roles of methionine, cysteine, and histidine were particularly exposed. Unlike in other halophilic fungi (Turk et al., 2004), hypersaline conditions did not influence the plasma membrane composition or fluidity. This first metabolomic profiling described the adaptation of a halophilic fungus to saturated NaCl conditions by changing the consumption of media nutrients, including a metabolic switch toward non-lipid sources and differences in the production of secondary metabolites. In summary, this study signals the beginning of an “omic,” and molecular understanding of how halophilic fungi adapt to most extreme salinities that are hostile to most eukaryotic microorganisms.

## Data Availability Statement

The datasets presented in this study can be found in online repositories. The names of the repository/repositories and accession number(s) can be found in the article/Supplementary Material.

## Author Contributions

RB-G conceptualized this study and wrote the manuscript. IJ-G, GV-M, TM-P, and YP-L analyzed the transcriptomic data. HS-J and FB-C determined the content of amino acids and fatty acids. AM-U performed the metabolomic analysis. NG-C, AL-L, JF-M, and MS-C participated in the interpretation of results. All authors contributed to the article and approved the submitted version.

## Funding

This work was supported by Consejo Nacional de Ciencia y Tecnología of Mexico (CONACyT): Project CONACyT 315114 and Project CONACyT-SEP CB-285816.

## Conflict of Interest

The authors declare that the research was conducted in the absence of any commercial or financial relationships that could be construed as a potential conflict of interest.

## Publisher’s Note

All claims expressed in this article are solely those of the authors and do not necessarily represent those of their affiliated organizations, or those of the publisher, the editors and the reviewers. Any product that may be evaluated in this article, or claim that may be made by its manufacturer, is not guaranteed or endorsed by the publisher.

## Footnotes

^1^ https://www.graphpad.com

^2^ https://gnps.ucsd.edu/ProteoSAFe/static/gnps-splash.jsp

## Supplementary Material

Supplementary Figure S1 *Aspergillus sydowii* growth curve for 12 days. Fungus was grown in Yeast Malt Agar (YMA): malt extract 10 g/L, yeast extract 4 g/L, dextrose 4 g/L, mycological peptone 5 g/L, agar 20 g/L. A growth curve based on the fungal dry weight was performed under optimal salinity condition (1 M NaCl). The culture reaches the middle exponential growth phase after 4 days. Mycelia and supernatants obtained from triplicate exponential phase cultures of the fungus (4-day-old cultures) were used for all experiments (i.e., determination of amino acids and fatty acids, transcriptomics, and metabolomics).

Supplementary Figure S2 Principal component analysis (PCA) and principal component variable grouping (PCVG) of *A. sydowii* grown under low and high-salt conditions. The scores plot (top left panel) was used to visualize the pattern among samples. The loading plots (top right panel) with PCVG were utilized to detect the features that contributed to the separation of the samples. The profile plots (bottom panels) were used to verify the intensity of the features differentially abundant in *A. sydowii* grown in low (1 M NaCl) and high salt conditions (5.13 M NaCl). The meaning of the circle’s color (excepting those in PCVG) is as following: black circles, *A. sydowii* in 1 M NaCl; green circles, *A. sydowii* in 5.13 M NaCl; red circles, control media 1 M NaCl; blue circles, control media 5.13 M NaCl, and pink circles, QC (pooled samples at equal amounts) samples. Figures were retrieved from MarkerView Software. Chemical structures were drawn by ChemDraw Professional 16.0.

Supplementary Table S1 Upregulated transcripts of *A. sydowii* EXF-12860 at 5.13 M NaCl compared to 1 M NaCl.

Supplementary Table S2 Downregulated transcripts of *A. sydowii* EXF-12860 at 5.13 M NaCl compared to 1 M NaCl.

Supplementary Table S3 Transcriptomic expression of *A. sydowii* EXF-12860’s genes related to amino acid metabolism at 5.13 M NaCl compared to 1 M NaCl.

Supplementary Table S4 Transcriptomic expression of *A. sydowii* EXF-12860’s genes related to fatty acids metabolism at 5.13 M NaCl compared to 1 M NaCl.

Supplementary Table S5 Transcriptomic expression of *A. sydowii* EXF-12860’s genes related to cell cycle, high glycerol pathway (HOG), compatible solutes, cell wall, pheromone synthesis, cellular membrane transporters, calcium pathway, and morphology at 5.13 M NaCl compared to 1 M NaCl.
