# Supplementary figures and images for: Surviving in the Brine: A Multi-Omics Approach for Understanding the Physiology of the Halophile Fungus Aspergillus sydowii at Saturated NaCl Concentration
Source: Front Microbiol. 2022 May 2;13:840408. doi: 10.3389/fmicb.2022.840408 (PMC9108488; doi:10.3389/fmicb.2022.840408)

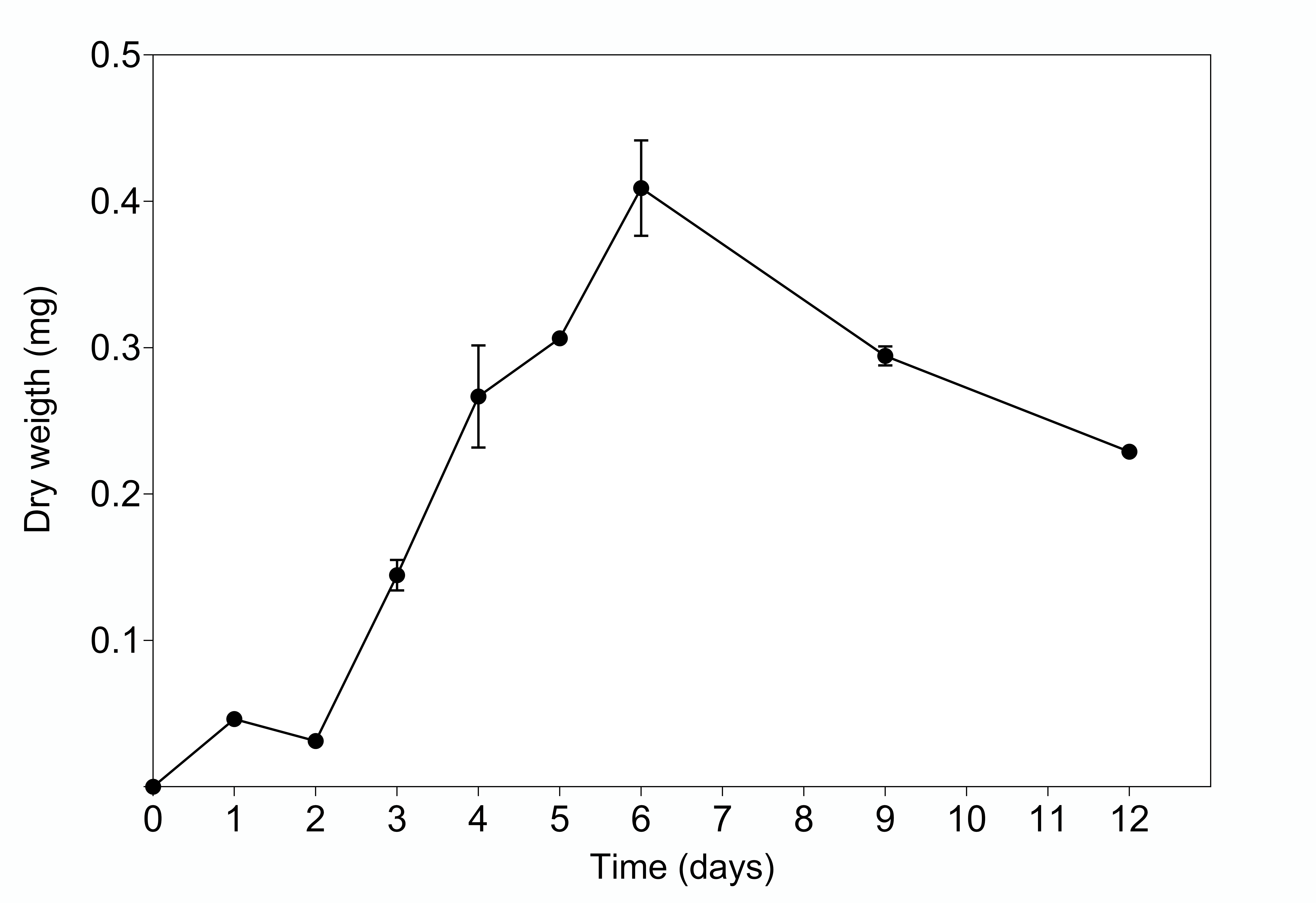

Supplement: Supplementary Figure S1 — Aspergillus sydowii growth curve for 12 days. Fungus was grown in Yeast Malt Agar (YMA): malt extract 10 g/L, yeast extract 4 g/L, dextrose 4 g/L, mycological peptone 5 g/L, agar 20 g/L. A growth curve based on the fungal dry weight was performed under optimal salinity condition (1 M NaCl). The culture reaches the middle exponential growth phase after 4 days. Mycelia and supernatants obtained from triplicate exponential phase cultures of the fungus (4-day-old cultures) were used for all experiments (i.e., determination of amino acids and fatty acids, transcriptomics, and metabolomics). [file Image_1.TIF]

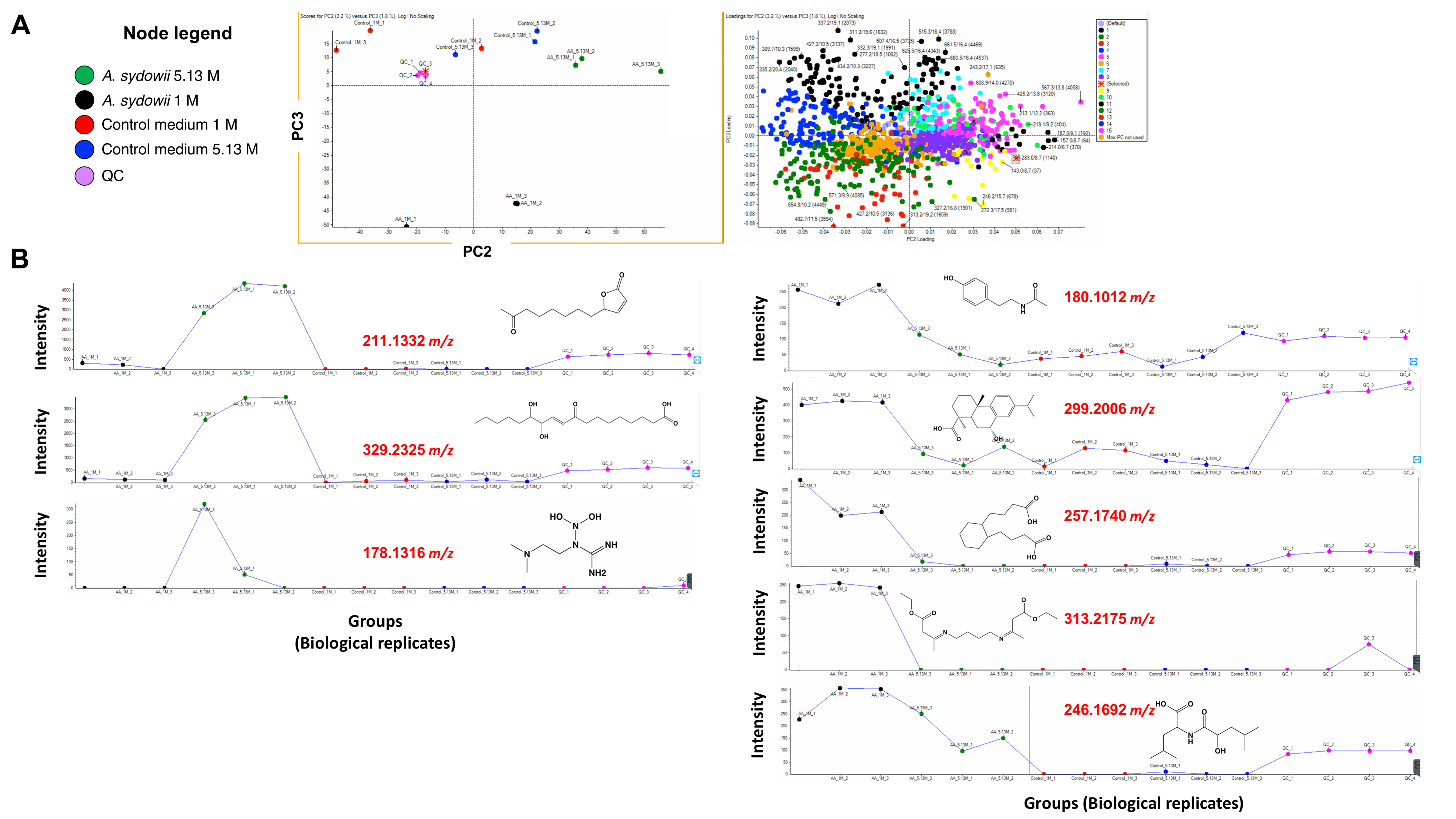

Supplement: Supplementary Figure S2 — Principal component analysis (PCA) and principal component variable grouping (PCVG) of A. sydowii grown under low and high-salt conditions. The scores plot (top left panel) was used to visualize the pattern among samples. The loading plots (top right panel) with PCVG were utilized to detect the features that contributed to the separation of the samples. The profile plots (bottom panels) were used to verify the intensity of the features differentially abundant in A. sydowii grown in low (1 M NaCl) and high salt conditions (5.13 M NaCl). The meaning of the circle’s color (excepting those in PCVG) is as following: black circles, A. sydowii in 1 M NaCl; green circles, A. sydowii in 5.13 M NaCl; red circles, control media 1 M NaCl; blue circles, control media 5.13 M NaCl, and pink circles, QC (pooled samples at equal amounts) samples. Figures were retrieved from MarkerView Software. Chemical structures were drawn by ChemDraw Professional 16.0. [file Image_2.TIF]
