# Supplementary material for: Surviving in the Brine: A Multi-Omics Approach for Understanding the Physiology of the Halophile Fungus Aspergillus sydowii at Saturated NaCl Concentration
Source: Front Microbiol. 2022 May 2;13:840408. doi: 10.3389/fmicb.2022.840408 (PMC9108488; doi:10.3389/fmicb.2022.840408)
Supplement: Supplementary Table S1 — Upregulated transcripts of A. sydowii EXF-12860 at 5.13 M NaCl compared to 1 M NaCl. [file Data_Sheet_1.PDF]

Supplementary Table S1. Upregulated transcripts of *Aspergillus sydowii* EXF-12860 at 5.13 M NaCl compared to 1 M NaCl.

Trinity ID	LogFC	FDR
TRINITY_DN802_c0_g1_i5	3,56	9,94E-03
TRINITY_DN510_c0_g1_i27	3,57	9,45E-03
TRINITY_DN1224_c0_g1_i4	3,57	9,54E-03
TRINITY_DN12591_c0_g1_i1	3,57	9,50E-03
TRINITY_DN3908_c0_g1_i2	3,58	9,58E-03
TRINITY_DN442_c1_g1_i18	3,59	9,42E-03
TRINITY_DN419_c1_g1_i18	3,59	9,63E-03
TRINITY_DN1955_c0_g1_i1	3,60	1,00E-02
TRINITY_DN565_c1_g2_i10	3,61	8,84E-03
TRINITY_DN5303_c0_g1_i1	3,61	8,84E-03
TRINITY_DN254_c0_g1_i8	3,61	9,27E-03
TRINITY_DN3373_c0_g1_i1	3,61	9,42E-03
TRINITY_DN499_c0_g2_i5	3,62	9,31E-03
TRINITY_DN1811_c0_g1_i3	3,62	8,88E-03
TRINITY_DN10819_c0_g1_i1	3,62	9,77E-03
TRINITY_DN5200_c0_g2_i1	3,62	9,38E-03
TRINITY_DN11405_c0_g1_i1	3,64	8,85E-03
TRINITY_DN2201_c0_g3_i1	3,65	9,53E-03
TRINITY_DN1499_c1_g1_i2	3,65	8,07E-03
TRINITY_DN1483_c0_g1_i15	3,65	8,36E-03
TRINITY_DN902_c0_g1_i4	3,65	7,91E-03
TRINITY_DN620_c0_g1_i2	3,65	8,86E-03
TRINITY_DN1879_c1_g1_i2	3,65	8,95E-03
TRINITY_DN288_c1_g1_i1	3,65	8,21E-03
TRINITY_DN1197_c0_g1_i6	3,66	8,26E-03
TRINITY_DN10513_c0_g1_i1	3,66	9,45E-03
TRINITY_DN1501_c0_g1_i2	3,66	8,76E-03
TRINITY_DN1661_c0_g1_i5	3,66	8,25E-03
TRINITY_DN2342_c0_g1_i3	3,66	8,26E-03
TRINITY_DN2935_c0_g1_i6	3,66	8,32E-03
TRINITY_DN4556_c0_g1_i1	3,67	9,44E-03
TRINITY_DN2833_c0_g2_i1	3,67	9,09E-03
TRINITY_DN4112_c0_g1_i2	3,67	8,11E-03
TRINITY_DN167_c1_g1_i2	3,67	8,96E-03
TRINITY_DN1971_c0_g1_i9	3,67	8,17E-03
TRINITY_DN673_c0_g1_i5	3,68	7,86E-03
TRINITY_DN1182_c0_g1_i1	3,68	7,62E-03
TRINITY_DN287_c0_g2_i3	3,69	9,74E-03
TRINITY_DN2996_c0_g1_i1	3,69	8,91E-03
TRINITY_DN11941_c0_g1_i1	3,69	7,38E-03
TRINITY_DN1869_c0_g1_i2	3,69	8,91E-03
TRINITY_DN1180_c0_g1_i6	3,70	9,52E-03
TRINITY_DN1339_c0_g1_i1	3,70	7,69E-03

Trinity ID	LogFC	FDR
TRINITY_DN7856_c0_g1_i1	3,71	8,38E-03
TRINITY_DN1332_c0_g1_i1	3,71	6,94E-03
TRINITY_DN111_c0_g1_i4	3,71	7,44E-03
TRINITY_DN896_c0_g2_i1	3,71	6,85E-03
TRINITY_DN2158_c1_g2_i1	3,71	8,78E-03
TRINITY_DN328_c0_g1_i3	3,72	6,93E-03
TRINITY_DN3205_c0_g1_i1	3,72	8,62E-03
TRINITY_DN10734_c0_g1_i1	3,72	8,63E-03
TRINITY_DN213_c0_g1_i5	3,73	7,11E-03
TRINITY_DN1685_c0_g1_i3	3,73	7,69E-03
TRINITY_DN167_c1_g1_i26	3,73	7,43E-03
TRINITY_DN277_c0_g1_i8	3,74	7,26E-03
TRINITY_DN1635_c0_g1_i3	3,74	8,17E-03
TRINITY_DN80_c0_g1_i2	3,74	7,47E-03
TRINITY_DN6156_c0_g1_i12	3,74	8,16E-03
TRINITY_DN1523_c0_g1_i2	3,74	6,72E-03
TRINITY_DN2140_c0_g1_i2	3,75	6,90E-03
TRINITY_DN76_c0_g2_i6	3,75	6,77E-03
TRINITY_DN131_c1_g1_i2	3,75	6,94E-03
TRINITY_DN2074_c0_g2_i1	3,75	7,84E-03
TRINITY_DN2742_c0_g1_i1	3,75	6,94E-03
TRINITY_DN3763_c0_g2_i4	3,75	7,35E-03
TRINITY_DN1909_c0_g3_i6	3,75	7,48E-03
TRINITY_DN213_c0_g1_i2	3,75	6,36E-03
TRINITY_DN167_c1_g2_i2	3,75	6,51E-03
TRINITY_DN598_c1_g1_i3	3,76	7,89E-03
TRINITY_DN1688_c1_g1_i2	3,76	8,75E-03
TRINITY_DN276_c0_g1_i26	3,76	6,94E-03
TRINITY_DN167_c1_g1_i16	3,76	6,70E-03
TRINITY_DN2186_c0_g1_i5	3,76	7,11E-03
TRINITY_DN3162_c0_g1_i1	3,76	7,77E-03
TRINITY_DN2515_c0_g1_i2	3,76	9,27E-03
TRINITY_DN8170_c0_g1_i1	3,76	8,35E-03
TRINITY_DN733_c0_g1_i29	3,77	7,22E-03
TRINITY_DN9836_c0_g1_i1	3,77	6,02E-03
TRINITY_DN520_c0_g1_i24	3,77	8,49E-03
TRINITY_DN180_c0_g1_i13	3,77	7,43E-03
TRINITY_DN5615_c0_g1_i1	3,77	5,98E-03
TRINITY_DN1662_c0_g1_i2	3,78	8,09E-03
TRINITY_DN567_c0_g1_i9	3,78	8,84E-03
TRINITY_DN6846_c0_g1_i1	3,78	7,49E-03
TRINITY_DN957_c0_g1_i3	3,78	6,63E-03

Trinity ID	LogFC	FDR
TRINITY_DN3687_c0_g1_i1	3,79	5,94E-03
TRINITY_DN277_c0_g1_i5	3,79	8,42E-03
TRINITY_DN143_c0_g1_i1	3,79	7,28E-03
TRINITY_DN1412_c0_g2_i2	3,79	5,73E-03
TRINITY_DN1065_c0_g1_i10	3,80	6,26E-03
TRINITY_DN9211_c0_g2_i1	3,80	5,60E-03
TRINITY_DN1499_c1_g2_i3	3,80	7,17E-03
TRINITY_DN2570_c0_g1_i2	3,80	7,12E-03
TRINITY_DN93_c0_g1_i37	3,81	8,10E-03
TRINITY_DN1337_c0_g1_i2	3,81	6,19E-03
TRINITY_DN104_c0_g1_i11	3,81	5,48E-03
TRINITY_DN598_c1_g1_i4	3,81	6,06E-03
TRINITY_DN429_c0_g1_i10	3,81	5,80E-03
TRINITY_DN4511_c0_g1_i7	3,82	8,83E-03
TRINITY_DN254_c0_g1_i6	3,82	6,20E-03
TRINITY_DN2342_c0_g1_i10	3,82	7,35E-03
TRINITY_DN5377_c1_g1_i3	3,82	6,10E-03
TRINITY_DN319_c0_g1_i12	3,82	5,88E-03
TRINITY_DN103_c3_g1_i5	3,82	5,87E-03
TRINITY_DN9208_c0_g2_i1	3,83	5,81E-03
TRINITY_DN737_c1_g1_i6	3,83	6,98E-03
TRINITY_DN5835_c0_g1_i1	3,83	9,27E-03
TRINITY_DN3007_c0_g2_i2	3,84	5,13E-03
TRINITY_DN1825_c0_g1_i2	3,84	5,89E-03
TRINITY_DN319_c0_g1_i7	3,84	5,42E-03
TRINITY_DN2960_c1_g1_i1	3,85	7,84E-03
TRINITY_DN2716_c0_g2_i1	3,85	5,32E-03
TRINITY_DN682_c0_g1_i6	3,85	6,77E-03
TRINITY_DN5961_c1_g1_i6	3,85	5,45E-03
TRINITY_DN1065_c0_g1_i5	3,86	5,38E-03
TRINITY_DN1701_c0_g1_i1	3,86	6,77E-03
TRINITY_DN10632_c0_g1_i1	3,87	5,31E-03
TRINITY_DN6932_c0_g2_i2	3,87	9,08E-03
TRINITY_DN233_c0_g1_i2	3,87	7,03E-03
TRINITY_DN545_c0_g1_i1	3,87	6,47E-03
TRINITY_DN5549_c1_g1_i1	3,87	5,87E-03
TRINITY_DN1825_c0_g1_i1	3,88	4,66E-03
TRINITY_DN688_c0_g1_i7	3,88	6,82E-03
TRINITY_DN2850_c0_g1_i2	3,88	4,99E-03
TRINITY_DN1262_c0_g1_i5	3,88	5,40E-03
TRINITY_DN2001_c1_g1_i1	3,89	5,10E-03
TRINITY_DN1204_c0_g1_i1	3,89	4,99E-03

Trinity ID	LogFC	FDR
TRINITY_DN917_c0_g2_i1	3,90	9,95E-03
TRINITY_DN11491_c0_g1_i1	3,90	5,26E-03
TRINITY_DN3595_c0_g1_i1	3,91	5,15E-03
TRINITY_DN3413_c0_g1_i2	3,91	6,31E-03
TRINITY_DN242_c0_g1_i20	3,91	4,90E-03
TRINITY_DN1446_c0_g1_i4	3,91	4,32E-03
TRINITY_DN3520_c0_g1_i1	3,92	4,17E-03
TRINITY_DN93_c0_g1_i55	3,92	4,22E-03
TRINITY_DN2222_c0_g1_i2	3,93	6,40E-03
TRINITY_DN93_c0_g1_i65	3,94	4,38E-03
TRINITY_DN1323_c0_g1_i4	3,94	5,68E-03
TRINITY_DN991_c0_g1_i5	3,95	5,07E-03
TRINITY_DN160_c1_g1_i4	3,95	4,45E-03
TRINITY_DN31_c0_g1_i5	3,95	5,01E-03
TRINITY_DN3668_c0_g1_i3	3,95	5,21E-03
TRINITY_DN2391_c0_g1_i3	3,95	6,17E-03
TRINITY_DN429_c0_g1_i9	3,95	4,13E-03
TRINITY_DN133_c0_g1_i13	3,95	5,03E-03
TRINITY_DN2252_c1_g1_i6	3,96	3,96E-03
TRINITY_DN2131_c0_g1_i15	3,96	4,59E-03
TRINITY_DN254_c0_g1_i23	3,96	5,59E-03
TRINITY_DN2691_c0_g1_i1	3,96	4,51E-03
TRINITY_DN12713_c0_g1_i1	3,97	4,52E-03
TRINITY_DN5193_c0_g1_i1	3,97	4,04E-03
TRINITY_DN745_c2_g1_i33	3,98	5,23E-03
TRINITY_DN93_c0_g1_i47	3,98	4,99E-03
TRINITY_DN9302_c0_g1_i1	3,99	5,12E-03
TRINITY_DN2914_c0_g1_i3	3,99	5,11E-03
TRINITY_DN1603_c0_g1_i2	3,99	4,06E-03
TRINITY_DN2625_c0_g2_i12	4,00	3,93E-03
TRINITY_DN1032_c0_g1_i1	4,01	3,70E-03
TRINITY_DN30_c1_g1_i1	4,01	3,96E-03
TRINITY_DN2101_c0_g1_i1	4,01	4,42E-03
TRINITY_DN2451_c1_g1_i1	4,01	8,06E-03
TRINITY_DN802_c0_g1_i2	4,01	4,18E-03
TRINITY_DN1958_c0_g1_i94	4,01	7,41E-03
TRINITY_DN12054_c0_g1_i1	4,01	3,77E-03
TRINITY_DN442_c0_g1_i8	4,02	4,98E-03
TRINITY_DN402_c0_g1_i2	4,02	3,62E-03
TRINITY_DN5421_c0_g1_i3	4,02	7,85E-03
TRINITY_DN1776_c2_g1_i4	4,03	3,44E-03
TRINITY_DN3294_c1_g1_i1	4,03	3,28E-03

Trinity ID	LogFC	FDR
TRINITY_DN254_c0_g1_i5	4,03	3,51E-03
TRINITY_DN195_c0_g1_i29	4,04	3,68E-03
TRINITY_DN915_c0_g1_i7	4,04	4,54E-03
TRINITY_DN5037_c0_g1_i1	4,04	5,21E-03
TRINITY_DN3908_c0_g1_i3	4,04	4,12E-03
TRINITY_DN1082_c0_g1_i13	4,04	3,34E-03
TRINITY_DN1004_c0_g1_i2	4,04	4,33E-03
TRINITY_DN2252_c1_g1_i2	4,04	3,23E-03
TRINITY_DN130_c0_g1_i3	4,04	3,65E-03
TRINITY_DN254_c0_g1_i4	4,05	3,26E-03
TRINITY_DN7165_c0_g1_i1	4,06	5,09E-03
TRINITY_DN5971_c0_g1_i1	4,06	4,20E-03
TRINITY_DN6170_c0_g1_i1	4,06	3,96E-03
TRINITY_DN1285_c0_g1_i43	4,06	4,16E-03
TRINITY_DN2569_c2_g3_i3	4,07	3,24E-03
TRINITY_DN991_c0_g1_i2	4,07	2,94E-03
TRINITY_DN1363_c0_g1_i1	4,08	3,58E-03
TRINITY_DN3539_c0_g1_i1	4,08	3,57E-03
TRINITY_DN254_c0_g1_i27	4,08	3,61E-03
TRINITY_DN1250_c0_g1_i10	4,09	2,93E-03
TRINITY_DN1082_c0_g1_i20	4,10	3,09E-03
TRINITY_DN1526_c0_g1_i1	4,10	3,14E-03
TRINITY_DN8717_c0_g1_i1	4,10	5,62E-03
TRINITY_DN741_c0_g1_i14	4,11	2,96E-03
TRINITY_DN1004_c0_g1_i1	4,11	3,21E-03
TRINITY_DN319_c0_g1_i6	4,11	2,86E-03
TRINITY_DN2336_c0_g1_i2	4,11	2,90E-03
TRINITY_DN1397_c2_g1_i1	4,13	2,78E-03
TRINITY_DN1367_c1_g1_i7	4,13	2,65E-03
TRINITY_DN12963_c0_g2_i1	4,14	6,74E-03
TRINITY_DN1335_c1_g1_i4	4,14	4,41E-03
TRINITY_DN93_c0_g1_i33	4,14	2,70E-03
TRINITY_DN1525_c0_g1_i2	4,14	2,64E-03
TRINITY_DN11178_c0_g1_i1	4,14	2,62E-03
TRINITY_DN1926_c0_g1_i1	4,14	2,90E-03
TRINITY_DN5556_c0_g2_i1	4,14	2,51E-03
TRINITY_DN919_c0_g1_i2	4,15	2,58E-03
TRINITY_DN2090_c0_g1_i3	4,15	4,59E-03
TRINITY_DN1206_c0_g1_i1	4,15	2,66E-03
TRINITY_DN12805_c0_g1_i1	4,15	2,50E-03
TRINITY_DN8213_c1_g1_i3	4,15	2,42E-03
TRINITY_DN688_c0_g1_i1	4,15	2,56E-03

Trinity ID	LogFC	FDR
TRINITY_DN2105_c0_g1_i4	4,15	3,81E-03
TRINITY_DN1726_c0_g1_i3	4,15	3,72E-03
TRINITY_DN863_c0_g1_i4	4,16	2,90E-03
TRINITY_DN89_c0_g1_i1	4,16	4,12E-03
TRINITY_DN950_c0_g1_i9	4,17	3,49E-03
TRINITY_DN848_c0_g1_i5	4,17	3,85E-03
TRINITY_DN2658_c0_g1_i3	4,17	2,59E-03
TRINITY_DN2210_c0_g1_i2	4,17	2,59E-03
TRINITY_DN9890_c0_g2_i1	4,17	4,54E-03
TRINITY_DN810_c0_g1_i1	4,17	3,00E-03
TRINITY_DN12823_c0_g1_i1	4,18	2,76E-03
TRINITY_DN602_c0_g1_i3	4,18	2,36E-03
TRINITY_DN2299_c0_g1_i1	4,18	2,47E-03
TRINITY_DN530_c0_g1_i39	4,19	2,73E-03
TRINITY_DN565_c1_g2_i7	4,19	2,20E-03
TRINITY_DN1070_c0_g1_i2	4,19	2,50E-03
TRINITY_DN3343_c0_g1_i1	4,19	4,50E-03
TRINITY_DN1446_c0_g1_i2	4,19	2,31E-03
TRINITY_DN2038_c0_g1_i3	4,19	2,73E-03
TRINITY_DN2935_c0_g1_i9	4,19	3,93E-03
TRINITY_DN2569_c2_g2_i1	4,19	2,90E-03
TRINITY_DN2182_c3_g1_i1	4,20	2,16E-03
TRINITY_DN472_c0_g1_i2	4,20	2,51E-03
TRINITY_DN499_c0_g2_i3	4,21	2,45E-03
TRINITY_DN11887_c0_g1_i1	4,21	4,66E-03
TRINITY_DN1311_c0_g1_i3	4,21	2,33E-03
TRINITY_DN6381_c1_g1_i1	4,21	3,71E-03
TRINITY_DN902_c0_g1_i5	4,21	3,24E-03
TRINITY_DN1651_c1_g1_i2	4,22	4,41E-03
TRINITY_DN2474_c0_g1_i2	4,22	2,03E-03
TRINITY_DN781_c0_g1_i4	4,22	3,30E-03
TRINITY_DN2035_c1_g1_i1	4,22	2,09E-03
TRINITY_DN1824_c0_g1_i2	4,22	2,47E-03
TRINITY_DN1791_c0_g1_i4	4,22	2,47E-03
TRINITY_DN5237_c0_g1_i1	4,23	4,34E-03
TRINITY_DN110_c0_g1_i28	4,23	2,30E-03
TRINITY_DN2431_c0_g1_i3	4,23	3,31E-03
TRINITY_DN802_c0_g1_i3	4,23	3,64E-03
TRINITY_DN1207_c0_g1_i11	4,23	2,03E-03
TRINITY_DN890_c0_g1_i5	4,23	2,05E-03
TRINITY_DN620_c0_g1_i13	4,23	3,06E-03
TRINITY_DN88_c0_g2_i5	4,24	1,94E-03
TRINITY_DN1754_c0_g2_i1	4,25	2,47E-03

Trinity ID	LogFC	FDR
TRINITY_DN2334_c0_g1_i1	4,26	2,31E-03
TRINITY_DN12104_c0_g1_i1	4,26	1,90E-03
TRINITY_DN5377_c1_g1_i2	4,26	2,33E-03
TRINITY_DN4304_c0_g1_i3	4,26	1,86E-03
TRINITY_DN2534_c1_g2_i6	4,27	2,23E-03
TRINITY_DN3846_c0_g1_i2	4,28	2,16E-03
TRINITY_DN721_c0_g1_i8	4,28	1,83E-03
TRINITY_DN953_c1_g1_i2	4,29	1,77E-03
TRINITY_DN1731_c0_g1_i6	4,29	2,39E-03
TRINITY_DN1776_c2_g1_i2	4,30	1,84E-03
TRINITY_DN310_c0_g1_i4	4,30	2,76E-03
TRINITY_DN1431_c2_g3_i1	4,31	3,72E-03
TRINITY_DN867_c0_g1_i8	4,31	1,76E-03
TRINITY_DN195_c0_g1_i9	4,32	1,63E-03
TRINITY_DN3563_c0_g2_i7	4,32	2,43E-03
TRINITY_DN2710_c0_g1_i2	4,32	2,43E-03
TRINITY_DN501_c0_g1_i4	4,33	1,89E-03
TRINITY_DN686_c1_g1_i4	4,33	1,55E-03
TRINITY_DN429_c1_g1_i3	4,33	1,84E-03
TRINITY_DN1991_c0_g1_i3	4,33	1,86E-03
TRINITY_DN276_c0_g1_i27	4,34	1,54E-03
TRINITY_DN987_c0_g1_i18	4,35	1,65E-03
TRINITY_DN3234_c0_g1_i3	4,37	1,84E-03
TRINITY_DN2569_c0_g1_i3	4,37	1,43E-03
TRINITY_DN2598_c0_g1_i3	4,38	1,66E-03
TRINITY_DN1443_c0_g1_i3	4,39	1,36E-03
TRINITY_DN1318_c0_g1_i5	4,39	1,92E-03
TRINITY_DN4861_c0_g2_i5	4,39	2,22E-03
TRINITY_DN741_c0_g1_i21	4,39	1,50E-03
TRINITY_DN953_c0_g1_i10	4,39	2,88E-03
TRINITY_DN1638_c0_g1_i2	4,40	1,60E-03
TRINITY_DN1200_c0_g1_i1	4,40	1,39E-03
TRINITY_DN530_c0_g1_i41	4,40	2,43E-03
TRINITY_DN2210_c0_g1_i3	4,40	1,29E-03
TRINITY_DN876_c0_g1_i1	4,40	1,43E-03
TRINITY_DN3385_c1_g1_i1	4,40	2,74E-03
TRINITY_DN1184_c0_g1_i4	4,41	1,80E-03
TRINITY_DN1753_c0_g2_i4	4,41	1,38E-03
TRINITY_DN1332_c0_g1_i2	4,42	1,31E-03
TRINITY_DN4050_c0_g1_i13	4,42	1,53E-03
TRINITY_DN639_c0_g2_i4	4,42	1,37E-03
TRINITY_DN1397_c2_g1_i3	4,43	1,56E-03
TRINITY_DN2827_c0_g1_i2	4,43	1,36E-03

Trinity ID	LogFC	FDR
TRINITY_DN3766_c0_g1_i2	4,43	1,22E-03
TRINITY_DN2069_c0_g1_i4	4,44	1,30E-03
TRINITY_DN13071_c0_g1_i1	4,44	1,43E-03
TRINITY_DN3678_c0_g1_i1	4,45	1,34E-03
TRINITY_DN90_c0_g1_i3	4,45	1,42E-03
TRINITY_DN5260_c0_g1_i1	4,45	1,53E-03
TRINITY_DN2072_c0_g1_i6	4,46	1,28E-03
TRINITY_DN2884_c1_g1_i2	4,46	1,82E-03
TRINITY_DN431_c0_g1_i3	4,46	2,77E-03
TRINITY_DN4273_c0_g1_i2	4,47	1,45E-03
TRINITY_DN3373_c0_g1_i2	4,47	1,47E-03
TRINITY_DN431_c0_g1_i1	4,47	2,68E-03
TRINITY_DN12972_c0_g1_i1	4,48	3,41E-03
TRINITY_DN1151_c0_g1_i4	4,49	1,49E-03
TRINITY_DN2714_c0_g1_i2	4,49	2,66E-03
TRINITY_DN11965_c0_g1_i1	4,49	1,30E-03
TRINITY_DN2928_c0_g1_i6	4,51	1,51E-03
TRINITY_DN233_c0_g1_i4	4,52	1,20E-03
TRINITY_DN12171_c0_g2_i1	4,52	2,51E-03
TRINITY_DN1446_c0_g1_i1	4,53	1,53E-03
TRINITY_DN2402_c0_g1_i23	4,53	1,09E-03
TRINITY_DN3197_c0_g1_i4	4,53	1,83E-03
TRINITY_DN310_c0_g1_i6	4,53	1,08E-03
TRINITY_DN5958_c0_g1_i1	4,53	2,27E-03
TRINITY_DN2569_c0_g1_i9	4,54	1,12E-03
TRINITY_DN4084_c0_g1_i1	4,54	9,87E-04
TRINITY_DN1958_c0_g1_i15	4,54	1,38E-03
TRINITY_DN593_c0_g2_i7	4,54	1,27E-03
TRINITY_DN276_c0_g1_i25	4,55	9,06E-04
TRINITY_DN241_c0_g1_i4	4,56	1,12E-03
TRINITY_DN739_c0_g1_i4	4,56	1,47E-03
TRINITY_DN1776_c3_g1_i5	4,56	1,60E-03
TRINITY_DN2010_c0_g1_i5	4,56	1,03E-03
TRINITY_DN151_c0_g1_i16	4,57	1,29E-03
TRINITY_DN2103_c0_g1_i1	4,58	1,57E-03
TRINITY_DN1204_c0_g1_i11	4,60	1,27E-03
TRINITY_DN4364_c1_g1_i1	4,60	1,39E-03
TRINITY_DN2205_c0_g1_i1	4,60	2,25E-03
TRINITY_DN2560_c0_g1_i5	4,61	1,25E-03
TRINITY_DN2431_c0_g1_i6	4,61	1,82E-03
TRINITY_DN2625_c0_g2_i5	4,61	7,83E-04
TRINITY_DN4861_c0_g2_i2	4,62	8,65E-04
TRINITY_DN791_c0_g1_i1	4,62	1,39E-03

Trinity ID	LogFC	FDR
TRINITY_DN10774_c0_g1_i1	4,62	9,11E-04
TRINITY_DN573_c0_g1_i6	4,63	1,07E-03
TRINITY_DN151_c0_g1_i17	4,63	2,50E-03
TRINITY_DN2010_c0_g1_i10	4,65	7,22E-04
TRINITY_DN1355_c0_g1_i2	4,65	1,55E-03
TRINITY_DN3935_c0_g1_i1	4,65	1,50E-03
TRINITY_DN519_c0_g1_i3	4,65	1,08E-03
TRINITY_DN341_c1_g1_i2	4,66	1,15E-03
TRINITY_DN3452_c0_g1_i2	4,66	1,70E-03
TRINITY_DN213_c0_g1_i11	4,67	7,13E-04
TRINITY_DN2972_c0_g1_i1	4,67	1,53E-03
TRINITY_DN4124_c0_g2_i1	4,67	7,90E-04
TRINITY_DN1821_c0_g4_i1	4,67	1,09E-03
TRINITY_DN1158_c0_g1_i1	4,67	1,13E-03
TRINITY_DN442_c0_g1_i1	4,68	7,05E-04
TRINITY_DN1250_c0_g1_i9	4,69	9,66E-04
TRINITY_DN5105_c1_g1_i1	4,69	9,37E-04
TRINITY_DN264_c0_g1_i24	4,70	7,12E-04
TRINITY_DN1078_c0_g1_i8	4,70	9,87E-04
TRINITY_DN721_c0_g1_i16	4,70	8,90E-04
TRINITY_DN1740_c0_g1_i1	4,71	1,02E-03
TRINITY_DN319_c0_g1_i10	4,71	6,06E-04
TRINITY_DN3941_c0_g1_i2	4,72	9,69E-04
TRINITY_DN2105_c0_g1_i12	4,72	6,47E-04
TRINITY_DN167_c0_g1_i7	4,73	5,64E-04
TRINITY_DN681_c0_g1_i14	4,73	7,03E-04
TRINITY_DN4044_c0_g1_i2	4,74	1,37E-03
TRINITY_DN12609_c0_g1_i1	4,74	6,81E-04
TRINITY_DN2777_c0_g1_i1	4,75	1,50E-03
TRINITY_DN6381_c0_g2_i1	4,75	7,05E-04
TRINITY_DN3296_c0_g1_i1	4,75	6,86E-04
TRINITY_DN1888_c0_g2_i3	4,75	1,12E-03
TRINITY_DN1519_c0_g1_i1	4,75	7,53E-04
TRINITY_DN2542_c0_g1_i21	4,76	7,99E-04
TRINITY_DN1335_c1_g1_i9	4,76	7,10E-04
TRINITY_DN1223_c0_g1_i1	4,76	6,06E-04
TRINITY_DN1401_c0_g1_i2	4,77	9,98E-04
TRINITY_DN2487_c0_g1_i2	4,78	7,34E-04
TRINITY_DN1446_c0_g1_i6	4,78	9,04E-04
TRINITY_DN863_c0_g1_i12	4,79	5,74E-04
TRINITY_DN11964_c0_g1_i1	4,79	1,19E-03
TRINITY_DN1667_c0_g1_i3	4,80	5,05E-04
TRINITY_DN1852_c0_g1_i6	4,80	5,36E-04

Trinity ID	LogFC	FDR
TRINITY_DN179_c0_g1_i9	4,81	6,19E-04
TRINITY_DN133_c0_g1_i5	4,81	5,64E-04
TRINITY_DN7059_c0_g1_i1	4,82	7,87E-04
TRINITY_DN419_c3_g1_i1	4,82	9,18E-04
TRINITY_DN254_c0_g1_i25	4,83	5,98E-04
TRINITY_DN10727_c0_g1_i1	4,83	4,25E-04
TRINITY_DN319_c0_g1_i9	4,84	5,00E-04
TRINITY_DN1168_c0_g2_i5	4,84	8,67E-04
TRINITY_DN626_c4_g1_i1	4,86	4,24E-04
TRINITY_DN10009_c0_g1_i1	4,87	1,59E-03
TRINITY_DN1092_c0_g1_i18	4,88	4,35E-04
TRINITY_DN430_c0_g1_i1	4,88	4,49E-04
TRINITY_DN2010_c0_g1_i16	4,88	3,88E-04
TRINITY_DN3385_c0_g1_i1	4,90	3,93E-04
TRINITY_DN1042_c0_g1_i3	4,90	4,20E-04
TRINITY_DN2291_c0_g1_i1	4,90	7,54E-04
TRINITY_DN9215_c0_g1_i1	4,91	4,57E-04
TRINITY_DN442_c0_g1_i7	4,92	4,49E-04
TRINITY_DN9561_c0_g1_i1	4,92	1,20E-03
TRINITY_DN1843_c0_g1_i1	4,95	3,32E-04
TRINITY_DN4873_c0_g1_i1	4,95	3,73E-04
TRINITY_DN5905_c0_g1_i1	4,95	3,34E-04
TRINITY_DN2515_c0_g1_i1	4,96	8,89E-04
TRINITY_DN1150_c0_g1_i1	4,97	9,27E-04
TRINITY_DN1958_c0_g1_i17	4,97	7,09E-04
TRINITY_DN291_c0_g1_i3	4,97	5,38E-04
TRINITY_DN1766_c0_g1_i3	4,98	4,06E-04
TRINITY_DN897_c0_g1_i2	4,98	7,16E-04
TRINITY_DN3520_c0_g1_i3	4,99	4,39E-04
TRINITY_DN4657_c0_g1_i1	4,99	3,30E-04
TRINITY_DN2436_c0_g1_i3	4,99	7,16E-04
TRINITY_DN264_c0_g1_i26	4,99	3,26E-04
TRINITY_DN1766_c0_g1_i1	4,99	8,40E-04
TRINITY_DN1780_c0_g1_i6	5,00	4,25E-04
TRINITY_DN2157_c0_g1_i6	5,00	3,58E-04
TRINITY_DN2598_c0_g1_i4	5,01	2,74E-04
TRINITY_DN802_c0_g1_i1	5,03	3,13E-04
TRINITY_DN1270_c0_g1_i1	5,04	2,39E-04
TRINITY_DN1824_c0_g1_i3	5,04	4,58E-04
TRINITY_DN2342_c0_g1_i7	5,05	3,14E-04
TRINITY_DN322_c0_g1_i16	5,07	7,15E-04
TRINITY_DN991_c0_g1_i3	5,08	3,18E-04
TRINITY_DN10120_c0_g1_i2	5,08	9,75E-04

Trinity ID	LogFC	FDR
TRINITY_DN1631_c1_g1_i2	5,08	2,43E-04
TRINITY_DN2883_c0_g1_i4	5,10	4,63E-04
TRINITY_DN1160_c0_g1_i10	5,12	2,17E-04
TRINITY_DN1131_c1_g1_i2	5,13	2,78E-04
TRINITY_DN2714_c0_g1_i1	5,13	3,87E-04
TRINITY_DN2736_c0_g1_i2	5,15	4,35E-04
TRINITY_DN1013_c0_g1_i2	5,15	8,15E-04
TRINITY_DN1135_c0_g1_i1	5,18	2,54E-04
TRINITY_DN10053_c1_g1_i1	5,20	1,89E-04
TRINITY_DN167_c0_g1_i19	5,20	2,66E-04
TRINITY_DN319_c0_g1_i1	5,22	2,14E-04
TRINITY_DN2788_c0_g1_i3	5,24	1,49E-04
TRINITY_DN4283_c0_g1_i1	5,24	3,21E-04
TRINITY_DN1793_c0_g1_i1	5,24	1,51E-04
TRINITY_DN2199_c0_g1_i4	5,25	2,17E-04
TRINITY_DN1852_c0_g1_i1	5,25	1,49E-04
TRINITY_DN1574_c0_g1_i2	5,25	1,47E-04
TRINITY_DN11463_c0_g1_i1	5,25	5,93E-04
TRINITY_DN342_c0_g1_i2	5,27	1,94E-04
TRINITY_DN3672_c0_g1_i1	5,27	1,34E-04
TRINITY_DN1926_c0_g1_i3	5,27	2,14E-04
TRINITY_DN1120_c0_g1_i4	5,28	3,78E-04
TRINITY_DN458_c0_g1_i6	5,28	2,38E-04
TRINITY_DN754_c0_g1_i4	5,28	1,81E-04
TRINITY_DN153_c0_g1_i3	5,30	1,24E-04
TRINITY_DN514_c0_g1_i5	5,31	1,55E-04
TRINITY_DN217_c0_g1_i14	5,32	2,31E-04
TRINITY_DN1164_c0_g1_i5	5,32	2,15E-04
TRINITY_DN133_c0_g1_i1	5,32	2,46E-04
TRINITY_DN209_c0_g1_i7	5,33	1,32E-04
TRINITY_DN217_c0_g1_i35	5,34	2,17E-04
TRINITY_DN1918_c0_g1_i1	5,34	7,98E-04
TRINITY_DN10581_c0_g1_i1	5,36	3,72E-04
TRINITY_DN883_c2_g1_i7	5,36	1,30E-04
TRINITY_DN1412_c0_g1_i4	5,36	1,43E-04
TRINITY_DN1335_c1_g1_i33	5,39	2,56E-04
TRINITY_DN1761_c1_g1_i4	5,39	3,94E-04
TRINITY_DN9468_c0_g1_i1	5,41	2,14E-04
TRINITY_DN1339_c0_g1_i2	5,41	1,29E-04
TRINITY_DN93_c0_g1_i59	5,42	2,38E-04
TRINITY_DN686_c1_g1_i5	5,42	1,58E-04
TRINITY_DN4068_c0_g1_i2	5,43	1,56E-04
TRINITY_DN442_c0_g1_i6	5,44	5,13E-04

Trinity ID	LogFC	FDR
TRINITY_DN926_c0_g1_i8	5,44	2,07E-04
TRINITY_DN2311_c0_g1_i1	5,44	6,70E-04
TRINITY_DN2157_c0_g1_i5	5,45	8,20E-05
TRINITY_DN497_c0_g1_i5	5,46	1,36E-04
TRINITY_DN966_c1_g1_i1	5,48	2,64E-04
TRINITY_DN565_c1_g2_i1	5,48	7,02E-05
TRINITY_DN1512_c0_g1_i1	5,50	8,46E-05
TRINITY_DN1102_c0_g1_i1	5,52	1,32E-04
TRINITY_DN1776_c2_g1_i8	5,53	9,36E-05
TRINITY_DN5359_c0_g1_i1	5,54	6,54E-05
TRINITY_DN1151_c0_g1_i6	5,54	1,82E-04
TRINITY_DN2063_c0_g1_i8	5,55	7,11E-05
TRINITY_DN290_c1_g1_i1	5,55	2,03E-04
TRINITY_DN8515_c0_g1_i1	5,56	4,79E-04
TRINITY_DN1027_c0_g1_i15	5,57	5,42E-05
TRINITY_DN603_c0_g1_i8	5,57	5,88E-05
TRINITY_DN276_c0_g1_i14	5,58	6,60E-05
TRINITY_DN1075_c1_g1_i1	5,59	1,39E-04
TRINITY_DN639_c1_g1_i1	5,62	5,88E-05
TRINITY_DN1027_c0_g1_i14	5,62	4,74E-05
TRINITY_DN1690_c0_g1_i15	5,63	7,60E-05
TRINITY_DN1700_c0_g1_i5	5,63	1,83E-04
TRINITY_DN2431_c0_g1_i4	5,67	7,97E-05
TRINITY_DN2106_c0_g1_i4	5,68	1,56E-04
TRINITY_DN264_c0_g1_i8	5,68	1,02E-04
TRINITY_DN1200_c0_g1_i4	5,69	6,39E-05
TRINITY_DN941_c0_g1_i7	5,71	6,09E-05
TRINITY_DN2502_c0_g1_i2	5,72	4,43E-05
TRINITY_DN93_c0_g1_i58	5,72	4,33E-05
TRINITY_DN88_c0_g2_i1	5,72	2,10E-04
TRINITY_DN88_c0_g2_i4	5,73	7,39E-05
TRINITY_DN2972_c0_g1_i3	5,75	1,24E-04
TRINITY_DN2345_c0_g1_i2	5,75	3,57E-05
TRINITY_DN1662_c0_g1_i1	5,79	4,15E-05
TRINITY_DN7459_c0_g2_i1	5,79	1,73E-04
TRINITY_DN1335_c1_g1_i20	5,80	6,44E-05
TRINITY_DN866_c1_g1_i2	5,82	6,41E-05
TRINITY_DN991_c0_g1_i7	5,84	2,90E-05
TRINITY_DN2314_c0_g1_i3	5,85	3,49E-05
TRINITY_DN1958_c0_g1_i71	5,86	5,33E-05
TRINITY_DN1243_c2_g1_i22	5,87	5,10E-05
TRINITY_DN2431_c0_g1_i2	5,87	1,68E-04
TRINITY_DN2314_c0_g1_i2	5,87	3,50E-04

Trinity ID	LogFC	FDR
TRINITY_DN1648_c0_g1_i16	5,87	3,39E-05
TRINITY_DN1975_c0_g1_i1	5,89	7,20E-05
TRINITY_DN505_c0_g1_i9	5,90	3,68E-05
TRINITY_DN2072_c0_g1_i3	5,91	5,47E-05
TRINITY_DN1975_c0_g1_i2	5,91	2,36E-05
TRINITY_DN1082_c0_g1_i5	5,91	3,88E-05
TRINITY_DN2290_c0_g1_i4	5,93	2,55E-05
TRINITY_DN3693_c0_g1_i3	5,96	4,47E-05
TRINITY_DN5036_c1_g1_i1	5,98	2,72E-04
TRINITY_DN104_c0_g1_i7	6,00	2,40E-05
TRINITY_DN2063_c0_g1_i2	6,02	3,36E-05
TRINITY_DN503_c0_g1_i11	6,02	1,99E-05
TRINITY_DN755_c1_g1_i5	6,04	7,14E-05
TRINITY_DN93_c0_g1_i27	6,04	7,05E-05
TRINITY_DN501_c0_g1_i9	6,06	2,41E-05
TRINITY_DN4819_c0_g1_i13	6,06	2,18E-05
TRINITY_DN4195_c0_g2_i1	6,11	4,61E-05
TRINITY_DN4819_c0_g1_i15	6,13	3,76E-05
TRINITY_DN2350_c0_g1_i1	6,15	9,36E-05
TRINITY_DN1027_c0_g1_i9	6,16	1,95E-05
TRINITY_DN2107_c0_g1_i18	6,17	1,97E-05
TRINITY_DN1390_c1_g1_i1	6,21	3,48E-05
TRINITY_DN1027_c0_g1_i16	6,23	8,64E-06
TRINITY_DN345_c0_g1_i9	6,23	2,52E-05
TRINITY_DN1135_c0_g1_i4	6,30	7,77E-06
TRINITY_DN167_c0_g1_i14	6,32	7,48E-06
TRINITY_DN715_c0_g1_i21	6,34	6,70E-05
TRINITY_DN603_c0_g1_i12	6,37	2,97E-05
TRINITY_DN1263_c0_g1_i1	6,37	8,82E-05
TRINITY_DN217_c0_g1_i22	6,42	1,36E-05
TRINITY_DN167_c0_g1_i17	6,42	8,54E-06
TRINITY_DN1198_c0_g2_i4	6,48	5,84E-06
TRINITY_DN4028_c0_g1_i8	6,55	2,05E-05
TRINITY_DN217_c0_g1_i18	6,57	2,52E-05
TRINITY_DN1027_c0_g1_i7	6,57	3,60E-06
TRINITY_DN1027_c0_g1_i1	6,59	3,31E-06
TRINITY_DN602_c0_g1_i14	6,60	1,45E-05
TRINITY_DN1376_c0_g1_i4	6,64	3,82E-05
TRINITY_DN3588_c0_g1_i1	6,65	4,76E-06
TRINITY_DN1493_c0_g1_i1	6,65	5,84E-06
TRINITY_DN685_c0_g1_i8	6,66	6,42E-05
TRINITY_DN1027_c0_g1_i10	6,67	2,62E-06
TRINITY_DN93_c0_g1_i39	6,73	8,33E-06

Trinity ID	LogFC	FDR
TRINITY_DN1285_c0_g1_i15	6,75	3,82E-06
TRINITY_DN60_c0_g1_i7	6,78	2,07E-06
TRINITY_DN62_c0_g1_i17	6,79	1,54E-05
TRINITY_DN924_c0_g1_i1	6,79	2,22E-06
TRINITY_DN1027_c0_g1_i8	6,86	1,62E-06
TRINITY_DN217_c0_g1_i27	6,89	2,29E-06
TRINITY_DN264_c0_g1_i22	6,93	3,37E-06
TRINITY_DN217_c0_g1_i32	6,96	3,22E-05
TRINITY_DN1274_c0_g3_i5	6,98	5,18E-06
TRINITY_DN1664_c0_g1_i5	6,99	5,12E-06
TRINITY_DN2290_c0_g1_i3	7,00	4,71E-06
TRINITY_DN1274_c0_g3_i3	7,03	1,93E-05
TRINITY_DN565_c1_g2_i2	7,04	1,50E-06
TRINITY_DN242_c0_g1_i32	7,07	3,18E-06
TRINITY_DN217_c0_g1_i21	7,08	9,35E-06
TRINITY_DN167_c0_g1_i20	7,09	2,61E-06
TRINITY_DN1027_c0_g1_i3	7,14	8,83E-07
TRINITY_DN48_c0_g1_i3	7,14	1,86E-06
TRINITY_DN1197_c0_g1_i7	7,20	3,63E-06
TRINITY_DN1975_c0_g1_i3	7,27	3,44E-06
TRINITY_DN345_c0_g1_i5	7,28	4,84E-06
TRINITY_DN1224_c0_g1_i1	7,35	3,05E-06
TRINITY_DN4170_c0_g1_i1	7,46	4,54E-06
TRINITY_DN1243_c2_g1_i8	7,48	1,14E-06
TRINITY_DN242_c0_g1_i48	7,49	9,10E-07
TRINITY_DN345_c0_g1_i1	7,52	6,93E-07
TRINITY_DN1151_c0_g1_i10	7,62	1,55E-06
TRINITY_DN9676_c0_g1_i1	7,63	7,26E-07
TRINITY_DN863_c0_g1_i5	7,65	3,55E-07
TRINITY_DN530_c0_g1_i34	7,67	3,08E-07
TRINITY_DN1664_c0_g1_i19	7,81	1,76E-06
TRINITY_DN1434_c0_g1_i1	7,83	2,67E-07
TRINITY_DN281_c0_g1_i21	7,84	1,96E-06
TRINITY_DN167_c0_g1_i9	7,88	9,46E-07
TRINITY_DN626_c2_g1_i6	7,97	4,54E-06
TRINITY_DN3911_c0_g1_i1	7,98	1,24E-04
TRINITY_DN4050_c0_g1_i59	8,02	5,40E-06
TRINITY_DN2569_c0_g1_i8	8,02	4,63E-07
TRINITY_DN153_c0_g1_i41	8,05	2,70E-07
TRINITY_DN4050_c0_g1_i29	8,15	7,71E-06
TRINITY_DN565_c1_g2_i6	8,20	7,43E-07
TRINITY_DN420_c0_g1_i3	8,24	2,37E-06
TRINITY_DN30_c1_g1_i3	8,27	5,47E-05

Trinity ID	LogFC	FDR
TRINITY_DN254_c0_g1_i28	8,29	4,65E-05
TRINITY_DN532_c0_g1_i17	8,31	4,50E-05
TRINITY_DN857_c0_g1_i4	8,35	5,56E-08
TRINITY_DN1519_c0_g1_i5	8,39	3,93E-05
TRINITY_DN298_c0_g1_i7	8,41	3,70E-05
TRINITY_DN48_c1_g2_i1	8,44	3,48E-05
TRINITY_DN1582_c0_g1_i2	8,46	3,36E-07
TRINITY_DN510_c0_g1_i14	8,49	3,11E-05
TRINITY_DN345_c0_g1_i2	8,52	1,23E-07
TRINITY_DN3927_c0_g1_i5	8,56	2,46E-05
TRINITY_DN510_c0_g1_i9	8,65	2,00E-05
TRINITY_DN1664_c0_g1_i4	8,71	3,07E-07
TRINITY_DN3519_c0_g2_i15	8,74	1,57E-05
TRINITY_DN12993_c0_g1_i1	8,74	6,22E-08
TRINITY_DN4050_c0_g1_i18	8,76	3,42E-07
TRINITY_DN1661_c2_g1_i1	8,77	5,59E-06
TRINITY_DN4029_c0_g1_i8	8,78	9,40E-07
TRINITY_DN2391_c0_g1_i1	8,82	1,22E-05
TRINITY_DN4050_c0_g1_i6	8,84	2,51E-07
TRINITY_DN1412_c0_g2_i1	8,85	1,14E-05
TRINITY_DN2350_c0_g1_i3	8,87	1,06E-05
TRINITY_DN4029_c0_g1_i2	8,88	7,12E-06
TRINITY_DN5661_c0_g1_i1	8,89	1,01E-05
TRINITY_DN254_c0_g1_i18	8,96	8,34E-06
TRINITY_DN751_c0_g1_i1	9,00	7,58E-06
TRINITY_DN1974_c0_g1_i1	9,01	1,92E-06
TRINITY_DN4053_c0_g1_i4	9,05	6,68E-06
TRINITY_DN51_c0_g1_i3	9,11	5,21E-06
TRINITY_DN980_c0_g1_i9	9,15	5,04E-06
TRINITY_DN3305_c0_g1_i5	9,16	4,97E-06
TRINITY_DN510_c0_g1_i23	9,20	4,47E-06
TRINITY_DN1027_c0_g1_i4	9,27	3,67E-06
TRINITY_DN3715_c0_g1_i3	9,29	3,50E-06
TRINITY_DN1664_c0_g1_i11	9,29	4,43E-07
TRINITY_DN153_c0_g1_i27	9,31	1,26E-06
TRINITY_DN829_c0_g2_i3	9,32	3,18E-06
TRINITY_DN1077_c0_g1_i5	9,37	2,76E-06
TRINITY_DN905_c0_g1_i5	9,41	2,51E-06
TRINITY_DN565_c1_g2_i8	9,41	2,51E-06
TRINITY_DN1726_c0_g1_i4	9,43	2,40E-06
TRINITY_DN323_c0_g1_i23	9,44	2,34E-06
TRINITY_DN1761_c1_g1_i5	9,45	3,45E-09
TRINITY_DN857_c0_g1_i5	9,48	1,24E-07

Trinity ID	LogFC	FDR
TRINITY_DN2290_c0_g1_i2	9,51	2,00E-06
TRINITY_DN217_c0_g1_i23	9,56	2,38E-08
TRINITY_DN93_c0_g1_i12	9,62	1,48E-06
TRINITY_DN638_c0_g2_i31	9,65	1,42E-06
TRINITY_DN1378_c0_g1_i1	9,67	1,31E-06
TRINITY_DN2786_c0_g2_i1	9,72	1,22E-06
TRINITY_DN1007_c0_g1_i19	9,73	1,19E-06
TRINITY_DN3000_c0_g1_i10	9,76	1,12E-06
TRINITY_DN95_c0_g1_i32	9,82	8,42E-07
TRINITY_DN2112_c0_g1_i19	9,84	9,10E-07
TRINITY_DN258_c0_g1_i14	9,89	8,66E-08
TRINITY_DN2072_c0_g1_i4	9,91	7,49E-07
TRINITY_DN322_c0_g1_i20	9,93	7,26E-07
TRINITY_DN1515_c0_g1_i2	9,96	6,63E-07
TRINITY_DN2072_c0_g1_i1	9,97	6,46E-07
TRINITY_DN1688_c0_g1_i3	9,98	6,40E-07
TRINITY_DN60_c0_g1_i8	10,01	5,84E-07
TRINITY_DN2910_c0_g1_i1	10,20	3,56E-07
TRINITY_DN914_c0_g1_i4	10,20	3,51E-07
TRINITY_DN3153_c0_g1_i2	10,23	3,33E-07
TRINITY_DN167_c1_g1_i31	10,26	3,12E-07
TRINITY_DN638_c0_g2_i20	10,28	3,02E-07
TRINITY_DN217_c0_g1_i20	10,43	3,84E-08
TRINITY_DN2474_c0_g1_i5	10,47	1,91E-07
TRINITY_DN792_c0_g1_i31	10,52	1,70E-07
TRINITY_DN281_c0_g1_i53	10,53	4,64E-10
TRINITY_DN167_c1_g1_i8	10,54	1,64E-07
TRINITY_DN1007_c0_g1_i3	10,54	1,64E-07
TRINITY_DN1434_c0_g1_i6	10,65	1,97E-09
TRINITY_DN217_c0_g1_i5	10,73	6,49E-08
TRINITY_DN590_c0_g1_i5	10,78	8,99E-08
TRINITY_DN1582_c0_g1_i4	10,85	1,09E-09
TRINITY_DN2177_c0_g1_i26	10,99	5,29E-08
TRINITY_DN167_c1_g1_i5	11,08	4,28E-08
TRINITY_DN980_c0_g1_i29	11,09	4,11E-08
TRINITY_DN2345_c0_g1_i1	11,14	3,72E-08
TRINITY_DN565_c1_g2_i9	11,18	3,30E-08
TRINITY_DN462_c0_g1_i10	11,23	3,00E-09
TRINITY_DN3178_c0_g1_i1	11,28	4,59E-10
TRINITY_DN322_c0_g1_i3	11,31	2,38E-08
TRINITY_DN702_c1_g1_i9	11,49	1,59E-08
TRINITY_DN1443_c0_g1_i1	11,62	1,09E-08
TRINITY_DN362_c0_g1_i9	11,71	8,80E-09

Trinity ID	LogFC	FDR
TRINITY_DN535_c0_g1_i1	11,73	8,36E-09
TRINITY_DN810_c0_g1_i40	11,79	6,13E-09
TRINITY_DN702_c1_g1_i8	11,80	6,97E-09
TRINITY_DN718_c0_g1_i13	11,85	6,25E-09
TRINITY_DN362_c0_g1_i5	11,93	1,80E-09
TRINITY_DN1330_c0_g1_i2	11,93	5,14E-09
TRINITY_DN362_c0_g1_i20	12,03	4,03E-09
TRINITY_DN1078_c0_g1_i15	12,13	3,14E-09
TRINITY_DN3153_c0_g1_i6	12,17	3,00E-09
TRINITY_DN3519_c0_g1_i1	12,37	1,80E-09
TRINITY_DN718_c0_g1_i9	12,45	1,44E-09
TRINITY_DN8_c0_g1_i3	12,61	9,85E-10
TRINITY_DN4132_c0_g1_i2	12,61	9,85E-10
TRINITY_DN1412_c0_g2_i4	12,65	9,20E-10
TRINITY_DN462_c0_g1_i13	12,65	9,20E-10
TRINITY_DN273_c0_g1_i3	12,66	9,20E-10
TRINITY_DN5216_c0_g2_i1	12,83	4,55E-10
TRINITY_DN462_c0_g1_i14	13,30	2,57E-10
TRINITY_DN2177_c0_g1_i8	13,82	7,29E-11
TRINITY_DN1957_c0_g1_i1	15,77	5,54E-13
