# Supplementary material for: Surviving in the Brine: A Multi-Omics Approach for Understanding the Physiology of the Halophile Fungus Aspergillus sydowii at Saturated NaCl Concentration
Source: Front Microbiol. 2022 May 2;13:840408. doi: 10.3389/fmicb.2022.840408 (PMC9108488; doi:10.3389/fmicb.2022.840408)
Supplement: Supplementary Table S2 — Downregulated transcripts of A. sydowii EXF-12860 at 5.13 M NaCl compared to 1 M NaCl. [file Data_Sheet_2.PDF]

Supplementary Table S2. Downregulated transcripts of *Aspergillus sydowii* EXF-12860 at 5.13 M NaCl compared to 1 M NaCl.

Trinity ID	LogFC	FDR
TRINITY_DN980_c0_g1_i5	-14,85	5,66E-12
TRINITY_DN892_c0_g1_i10	-14,33	1,37E-11
TRINITY_DN1916_c0_g2_i11	-14,23	2,41E-11
TRINITY_DN110_c0_g1_i6	-13,99	4,73E-11
TRINITY_DN590_c0_g1_i4	-13,93	5,54E-13
TRINITY_DN826_c0_g1_i1	-13,88	4,01E-12
TRINITY_DN1299_c1_g1_i2	-13,57	1,31E-10
TRINITY_DN2039_c0_g1_i1	-13,51	1,51E-10
TRINITY_DN1767_c1_g1_i10	-13,44	7,96E-11
TRINITY_DN2261_c0_g1_i10	-13,44	1,77E-10
TRINITY_DN4440_c0_g1_i4	-13,25	2,86E-10
TRINITY_DN2232_c0_g1_i5	-13,18	3,13E-10
TRINITY_DN4438_c0_g1_i1	-13,04	4,55E-10
TRINITY_DN2783_c0_g1_i10	-13,03	3,13E-10
TRINITY_DN1916_c0_g2_i3	-12,95	5,19E-10
TRINITY_DN1648_c0_g1_i18	-12,89	5,71E-10
TRINITY_DN3480_c1_g1_i4	-12,89	5,71E-10
TRINITY_DN110_c0_g1_i39	-12,87	5,71E-10
TRINITY_DN2262_c0_g1_i1	-12,82	6,36E-10
TRINITY_DN353_c0_g1_i12	-12,72	8,31E-10
TRINITY_DN4050_c0_g1_i46	-12,69	8,85E-10
TRINITY_DN4306_c0_g1_i1	-12,60	9,85E-10
TRINITY_DN556_c0_g1_i13	-12,50	1,31E-09
TRINITY_DN3480_c0_g1_i1	-12,45	1,44E-09
TRINITY_DN2195_c0_g2_i4	-12,35	1,84E-09
TRINITY_DN13_c0_g2_i10	-12,16	3,00E-09
TRINITY_DN1618_c0_g1_i53	-12,15	3,00E-09
TRINITY_DN490_c0_g1_i20	-12,14	3,00E-09
TRINITY_DN610_c0_g1_i12	-12,05	3,89E-09
TRINITY_DN48_c0_g1_i4	-11,98	4,70E-09
TRINITY_DN583_c0_g1_i10	-11,97	4,87E-09
TRINITY_DN892_c0_g1_i9	-11,94	2,53E-09
TRINITY_DN326_c0_g1_i1	-11,86	1,37E-11
TRINITY_DN8_c0_g1_i14	-11,81	6,97E-09
TRINITY_DN48_c1_g1_i2	-11,80	6,97E-09
TRINITY_DN2195_c0_g2_i3	-11,80	6,97E-09
TRINITY_DN1136_c0_g1_i3	-11,76	4,88E-09
TRINITY_DN1694_c0_g1_i2	-11,75	8,02E-09
TRINITY_DN713_c0_g1_i7	-11,72	6,97E-09
TRINITY_DN1096_c1_g1_i5	-11,71	5,77E-09
TRINITY_DN1842_c0_g1_i1	-11,70	7,22E-10
TRINITY_DN3475_c0_g1_i1	-11,69	9,17E-09

Trinity ID	LogFC	FDR
TRINITY_DN3412_c0_g1_i10	-11,69	9,17E-09
TRINITY_DN1489_c0_g1_i4	-11,67	9,50E-09
TRINITY_DN447_c0_g1_i1	-11,67	9,50E-09
TRINITY_DN1316_c0_g1_i5	-11,59	8,69E-09
TRINITY_DN2107_c0_g1_i21	-11,51	1,56E-08
TRINITY_DN759_c0_g1_i6	-11,50	1,56E-08
TRINITY_DN437_c0_g1_i14	-11,49	1,57E-08
TRINITY_DN36_c0_g2_i3	-11,49	1,59E-08
TRINITY_DN447_c0_g1_i5	-11,48	1,61E-08
TRINITY_DN2_c0_g1_i25	-11,47	1,65E-08
TRINITY_DN2232_c0_g1_i7	-11,46	1,67E-08
TRINITY_DN514_c0_g2_i3	-11,42	1,87E-08
TRINITY_DN560_c0_g1_i3	-11,42	1,88E-08
TRINITY_DN2261_c0_g1_i1	-11,37	2,09E-08
TRINITY_DN4050_c0_g1_i15	-11,36	2,16E-08
TRINITY_DN682_c0_g3_i4	-11,35	2,22E-08
TRINITY_DN1249_c0_g1_i5	-11,33	2,32E-08
TRINITY_DN511_c0_g1_i19	-11,31	2,38E-08
TRINITY_DN661_c0_g1_i18	-11,30	2,38E-08
TRINITY_DN1717_c0_g1_i1	-11,30	2,38E-08
TRINITY_DN709_c0_g1_i3	-11,29	2,41E-08
TRINITY_DN1285_c0_g1_i16	-11,27	5,14E-09
TRINITY_DN1900_c0_g2_i5	-11,27	2,54E-08
TRINITY_DN892_c0_g1_i11	-11,27	2,46E-08
TRINITY_DN380_c0_g1_i3	-11,22	2,99E-08
TRINITY_DN1717_c0_g1_i19	-11,19	3,24E-08
TRINITY_DN48_c0_g1_i10	-11,14	3,72E-08
TRINITY_DN454_c0_g1_i17	-11,13	3,84E-08
TRINITY_DN3783_c0_g2_i1	-11,12	2,38E-08
TRINITY_DN546_c0_g1_i50	-11,12	3,91E-08
TRINITY_DN912_c0_g1_i9	-11,10	4,03E-08
TRINITY_DN1618_c0_g1_i29	-11,10	4,03E-08
TRINITY_DN2660_c0_g1_i7	-11,08	4,28E-08
TRINITY_DN1753_c0_g2_i3	-11,07	4,30E-08
TRINITY_DN829_c0_g2_i1	-11,06	4,41E-08
TRINITY_DN2261_c0_g1_i3	-11,05	4,58E-08
TRINITY_DN2647_c0_g1_i2	-11,03	4,65E-08
TRINITY_DN514_c0_g2_i6	-11,01	4,96E-08
TRINITY_DN546_c0_g1_i46	-11,01	5,01E-08
TRINITY_DN1243_c2_g1_i16	-10,96	5,56E-08
TRINITY_DN117_c0_g1_i4	-10,96	5,67E-08
TRINITY_DN1769_c0_g1_i5	-10,95	5,83E-08

Trinity ID	LogFC	FDR
TRINITY_DN1406_c0_g1_i11	-10,95	5,83E-08
TRINITY_DN2402_c0_g1_i13	-10,95	5,83E-08
TRINITY_DN4706_c0_g1_i2	-10,94	5,98E-08
TRINITY_DN3690_c0_g1_i1	-10,87	7,31E-08
TRINITY_DN556_c0_g1_i6	-10,86	7,45E-08
TRINITY_DN1648_c0_g1_i6	-10,85	7,55E-08
TRINITY_DN2440_c0_g1_i1	-10,83	7,92E-08
TRINITY_DN3301_c0_g1_i4	-10,83	7,92E-08
TRINITY_DN39_c0_g1_i12	-10,83	7,70E-08
TRINITY_DN826_c0_g1_i4	-10,83	5,71E-10
TRINITY_DN235_c0_g1_i2	-10,83	8,02E-08
TRINITY_DN322_c0_g1_i25	-10,81	8,43E-08
TRINITY_DN13_c0_g2_i4	-10,80	8,61E-08
TRINITY_DN2107_c0_g1_i3	-10,79	8,86E-08
TRINITY_DN3412_c0_g1_i4	-10,78	8,99E-08
TRINITY_DN4028_c0_g1_i1	-10,77	9,00E-08
TRINITY_DN1140_c0_g1_i3	-10,77	9,00E-08
TRINITY_DN1717_c0_g1_i25	-10,76	4,45E-08
TRINITY_DN3789_c0_g1_i5	-10,74	9,98E-08
TRINITY_DN48_c0_g1_i2	-10,73	1,01E-07
TRINITY_DN665_c0_g1_i33	-10,72	1,02E-07
TRINITY_DN362_c1_g1_i6	-10,72	1,04E-07
TRINITY_DN772_c0_g1_i5	-10,71	1,04E-07
TRINITY_DN439_c1_g1_i2	-10,71	1,04E-07
TRINITY_DN3436_c0_g1_i3	-10,70	1,07E-07
TRINITY_DN7922_c0_g1_i4	-10,70	1,07E-07
TRINITY_DN3412_c0_g1_i1	-10,68	1,13E-07
TRINITY_DN1234_c1_g1_i4	-10,64	1,25E-07
TRINITY_DN2940_c0_g1_i1	-10,64	1,26E-07
TRINITY_DN638_c0_g2_i19	-10,62	1,20E-07
TRINITY_DN941_c0_g1_i5	-10,61	1,36E-07
TRINITY_DN2783_c0_g1_i12	-10,61	1,36E-07
TRINITY_DN839_c0_g1_i2	-10,60	1,40E-07
TRINITY_DN2718_c0_g1_i5	-10,57	1,55E-07
TRINITY_DN4050_c0_g1_i56	-10,56	1,58E-07
TRINITY_DN1900_c0_g2_i1	-10,56	1,31E-07
TRINITY_DN3480_c0_g1_i4	-10,55	1,60E-07
TRINITY_DN486_c0_g1_i42	-10,55	1,59E-07
TRINITY_DN3603_c0_g1_i1	-10,54	1,62E-07
TRINITY_DN473_c0_g1_i3	-10,53	1,67E-07
TRINITY_DN470_c1_g2_i6	-10,53	1,67E-07
TRINITY_DN1140_c0_g1_i11	-10,52	1,70E-07

Trinity ID	LogFC	FDR
TRINITY_DN1088_c0_g1_i6	-10,52	1,70E-07
TRINITY_DN480_c0_g1_i8	-10,51	1,73E-07
TRINITY_DN2502_c0_g1_i3	-10,47	1,91E-07
TRINITY_DN3075_c0_g1_i2	-10,47	1,91E-07
TRINITY_DN124_c2_g1_i2	-10,45	2,05E-07
TRINITY_DN2555_c0_g1_i1	-10,45	3,04E-09
TRINITY_DN60_c0_g1_i4	-10,44	2,07E-07
TRINITY_DN2811_c0_g1_i5	-10,43	2,16E-07
TRINITY_DN912_c0_g1_i4	-10,42	2,19E-07
TRINITY_DN3726_c0_g1_i3	-10,42	2,10E-07
TRINITY_DN486_c0_g1_i41	-10,41	2,24E-07
TRINITY_DN1022_c0_g1_i8	-10,41	2,24E-07
TRINITY_DN605_c0_g1_i6	-10,41	2,24E-07
TRINITY_DN2647_c0_g1_i1	-10,39	2,38E-07
TRINITY_DN1214_c0_g1_i10	-10,38	2,43E-07
TRINITY_DN530_c0_g1_i21	-10,38	2,38E-07
TRINITY_DN447_c0_g1_i3	-10,38	2,44E-07
TRINITY_DN511_c0_g1_i25	-10,35	2,57E-07
TRINITY_DN115_c0_g1_i2	-10,34	2,67E-07
TRINITY_DN3567_c0_g1_i1	-10,34	2,69E-07
TRINITY_DN571_c1_g1_i6	-10,33	2,71E-07
TRINITY_DN4333_c0_g1_i2	-10,33	2,73E-07
TRINITY_DN6_c0_g3_i4	-10,32	2,77E-07
TRINITY_DN5613_c0_g1_i2	-10,31	2,91E-07
TRINITY_DN2789_c0_g1_i2	-10,30	2,97E-07
TRINITY_DN3480_c0_g1_i3	-10,29	3,01E-07
TRINITY_DN353_c0_g1_i6	-10,28	3,03E-07
TRINITY_DN2041_c0_g1_i3	-10,28	3,05E-07
TRINITY_DN2647_c0_g1_i6	-10,27	3,07E-07
TRINITY_DN48_c1_g1_i8	-10,27	3,08E-07
TRINITY_DN2660_c0_g1_i11	-10,27	3,08E-07
TRINITY_DN3185_c0_g1_i2	-10,27	3,08E-07
TRINITY_DN2606_c0_g1_i4	-10,27	2,54E-07
TRINITY_DN1687_c0_g1_i1	-10,26	3,12E-07
TRINITY_DN2238_c0_g2_i2	-10,26	3,13E-07
TRINITY_DN1236_c0_g1_i2	-10,24	3,24E-07
TRINITY_DN194_c0_g1_i1	-10,24	4,89E-09
TRINITY_DN124_c2_g1_i3	-10,23	3,37E-07
TRINITY_DN1440_c0_g1_i3	-10,22	3,42E-07
TRINITY_DN1287_c0_g1_i7	-10,22	3,42E-07
TRINITY_DN2203_c0_g1_i2	-10,22	3,42E-07
TRINITY_DN1995_c0_g1_i3	-10,22	3,44E-07

Trinity ID	LogFC	FDR
TRINITY_DN511_c0_g1_i34	-10,21	3,45E-07
TRINITY_DN4307_c0_g1_i3	-10,21	3,51E-07
TRINITY_DN1204_c0_g1_i5	-10,18	9,98E-08
TRINITY_DN1505_c0_g1_i3	-10,17	3,81E-07
TRINITY_DN863_c1_g1_i3	-10,16	3,90E-07
TRINITY_DN1717_c0_g1_i27	-10,16	3,93E-07
TRINITY_DN699_c0_g1_i19	-10,15	2,38E-08
TRINITY_DN4217_c0_g1_i2	-10,15	4,00E-07
TRINITY_DN682_c0_g3_i5	-10,15	4,03E-07
TRINITY_DN1007_c0_g1_i8	-10,14	4,14E-07
TRINITY_DN44_c0_g1_i19	-10,14	4,16E-07
TRINITY_DN2148_c0_g1_i3	-10,13	4,19E-07
TRINITY_DN1090_c0_g1_i3	-10,12	4,30E-07
TRINITY_DN388_c1_g1_i3	-10,12	4,30E-07
TRINITY_DN1302_c0_g1_i4	-10,12	4,38E-07
TRINITY_DN1342_c0_g1_i5	-10,11	4,43E-07
TRINITY_DN2907_c0_g1_i2	-10,11	4,43E-07
TRINITY_DN4361_c0_g1_i3	-10,04	5,42E-07
TRINITY_DN480_c0_g1_i5	-10,03	5,53E-07
TRINITY_DN2373_c0_g1_i6	-10,03	5,58E-07
TRINITY_DN62_c0_g1_i15	-10,03	5,62E-07
TRINITY_DN980_c0_g1_i23	-10,01	5,84E-07
TRINITY_DN1096_c1_g1_i3	-10,01	5,42E-07
TRINITY_DN1195_c0_g1_i5	-10,01	5,89E-07
TRINITY_DN2152_c0_g1_i4	-10,00	5,94E-07
TRINITY_DN1773_c0_g1_i2	-10,00	5,99E-07
TRINITY_DN1889_c1_g1_i1	-9,96	6,63E-07
TRINITY_DN3743_c0_g1_i2	-9,94	6,78E-07
TRINITY_DN1487_c0_g1_i12	-9,92	7,31E-07
TRINITY_DN182_c0_g1_i6	-9,92	2,89E-07
TRINITY_DN1007_c0_g1_i10	-9,92	7,37E-07
TRINITY_DN3214_c0_g1_i4	-9,92	7,43E-07
TRINITY_DN437_c0_g1_i23	-9,91	7,51E-07
TRINITY_DN4050_c0_g1_i47	-9,91	7,51E-07
TRINITY_DN676_c0_g1_i8	-9,89	7,99E-07
TRINITY_DN2305_c0_g1_i12	-9,88	8,28E-07
TRINITY_DN605_c0_g1_i4	-9,87	3,00E-09
TRINITY_DN3056_c0_g1_i2	-9,87	8,34E-07
TRINITY_DN883_c2_g1_i3	-9,86	8,51E-07
TRINITY_DN2232_c0_g1_i12	-9,84	9,20E-07
TRINITY_DN2292_c0_g1_i4	-9,83	9,30E-07
TRINITY_DN2371_c0_g1_i2	-9,83	9,30E-07

Trinity ID	LogFC	FDR
TRINITY_DN243_c1_g1_i1	-9,83	9,30E-07
TRINITY_DN839_c0_g1_i3	-9,83	9,30E-07
TRINITY_DN1985_c0_g1_i8	-9,82	9,47E-07
TRINITY_DN1483_c0_g2_i2	-9,80	1,01E-06
TRINITY_DN3480_c0_g1_i9	-9,77	1,09E-06
TRINITY_DN4013_c0_g1_i2	-9,77	1,09E-06
TRINITY_DN211_c0_g1_i1	-9,77	1,09E-06
TRINITY_DN853_c0_g1_i8	-9,76	1,09E-06
TRINITY_DN725_c4_g1_i2	-9,76	1,11E-06
TRINITY_DN2573_c0_g1_i3	-9,75	1,12E-06
TRINITY_DN2474_c0_g1_i6	-9,75	1,12E-06
TRINITY_DN437_c0_g1_i11	-9,74	1,15E-06
TRINITY_DN3077_c1_g1_i1	-9,74	1,18E-06
TRINITY_DN3480_c1_g1_i7	-9,73	1,19E-06
TRINITY_DN3731_c0_g1_i2	-9,72	1,20E-06
TRINITY_DN48_c0_g1_i7	-9,72	1,21E-06
TRINITY_DN1718_c0_g1_i1	-9,72	1,21E-06
TRINITY_DN2887_c0_g1_i5	-9,72	1,22E-06
TRINITY_DN1437_c0_g1_i3	-9,71	1,23E-06
TRINITY_DN2560_c0_g1_i4	-9,71	1,23E-06
TRINITY_DN2655_c0_g1_i5	-9,71	1,23E-06
TRINITY_DN4058_c0_g1_i2	-9,71	1,23E-06
TRINITY_DN292_c1_g1_i8	-9,71	1,23E-06
TRINITY_DN97_c0_g3_i8	-9,71	1,23E-06
TRINITY_DN3163_c0_g1_i5	-9,71	1,23E-06
TRINITY_DN470_c1_g2_i3	-9,70	1,24E-06
TRINITY_DN605_c0_g1_i5	-9,70	1,26E-06
TRINITY_DN1573_c0_g1_i8	-9,70	1,26E-06
TRINITY_DN448_c0_g1_i6	-9,69	1,26E-06
TRINITY_DN2928_c0_g1_i4	-9,69	1,27E-06
TRINITY_DN1287_c0_g2_i2	-9,69	1,27E-06
TRINITY_DN81_c0_g1_i3	-9,69	1,27E-06
TRINITY_DN245_c1_g3_i1	-9,68	1,30E-06
TRINITY_DN1195_c1_g1_i2	-9,68	1,30E-06
TRINITY_DN4519_c0_g1_i5	-9,68	1,31E-06
TRINITY_DN2159_c0_g1_i2	-9,67	1,31E-06
TRINITY_DN3872_c0_g1_i2	-9,67	1,33E-06
TRINITY_DN2526_c0_g1_i1	-9,66	1,39E-06
TRINITY_DN167_c1_g1_i1	-9,65	1,40E-06
TRINITY_DN2416_c0_g1_i6	-9,63	8,59E-07
TRINITY_DN2304_c1_g1_i1	-9,63	1,48E-06
TRINITY_DN2957_c0_g1_i3	-9,63	1,48E-06

Trinity ID	LogFC	FDR
TRINITY_DN1956_c0_g1_i4	-9,62	1,50E-06
TRINITY_DN4413_c0_g1_i1	-9,62	1,50E-06
TRINITY_DN4911_c0_g1_i3	-9,62	1,51E-06
TRINITY_DN1213_c0_g1_i5	-9,62	1,51E-06
TRINITY_DN1362_c1_g1_i1	-9,59	1,62E-06
TRINITY_DN4512_c0_g1_i2	-9,58	1,63E-06
TRINITY_DN8_c0_g1_i18	-9,58	1,16E-06
TRINITY_DN1549_c0_g1_i2	-9,58	1,67E-06
TRINITY_DN490_c0_g1_i28	-9,58	1,65E-06
TRINITY_DN2811_c0_g1_i2	-9,58	1,67E-06
TRINITY_DN462_c0_g1_i11	-9,57	1,69E-06
TRINITY_DN1899_c0_g1_i6	-9,57	1,71E-06
TRINITY_DN573_c0_g1_i9	-9,57	1,71E-06
TRINITY_DN3872_c0_g1_i4	-9,56	1,76E-06
TRINITY_DN1822_c1_g1_i3	-9,54	1,81E-06
TRINITY_DN1899_c0_g1_i5	-9,54	1,81E-06
TRINITY_DN486_c0_g1_i16	-9,54	1,86E-06
TRINITY_DN511_c0_g1_i27	-9,54	1,86E-06
TRINITY_DN1610_c0_g1_i2	-9,52	1,94E-06
TRINITY_DN470_c1_g2_i5	-9,52	1,96E-06
TRINITY_DN2587_c0_g1_i1	-9,52	1,96E-06
TRINITY_DN1074_c0_g1_i18	-9,51	1,96E-06
TRINITY_DN642_c0_g1_i5	-9,51	1,98E-06
TRINITY_DN2587_c0_g1_i5	-9,51	2,00E-06
TRINITY_DN255_c0_g1_i1	-9,50	2,00E-06
TRINITY_DN2373_c0_g1_i11	-9,50	2,00E-06
TRINITY_DN2795_c0_g1_i1	-9,49	2,04E-06
TRINITY_DN3480_c0_g1_i5	-9,49	2,07E-06
TRINITY_DN1490_c0_g1_i5	-9,49	2,07E-06
TRINITY_DN2177_c0_g1_i4	-9,49	3,22E-07
TRINITY_DN7815_c0_g1_i1	-9,48	2,09E-06
TRINITY_DN605_c0_g1_i7	-9,48	2,11E-06
TRINITY_DN2071_c0_g1_i3	-9,48	2,11E-06
TRINITY_DN530_c0_g1_i22	-9,47	2,14E-06
TRINITY_DN1391_c0_g1_i2	-9,46	2,21E-06
TRINITY_DN1490_c0_g1_i6	-9,46	2,24E-06
TRINITY_DN2783_c0_g1_i4	-9,45	2,09E-08
TRINITY_DN2655_c0_g1_i12	-9,44	2,34E-06
TRINITY_DN359_c0_g1_i4	-9,43	2,34E-06
TRINITY_DN3057_c0_g1_i1	-9,43	2,40E-06
TRINITY_DN3712_c0_g1_i5	-9,42	2,44E-06
TRINITY_DN211_c0_g1_i3	-9,42	2,48E-06

Trinity ID	LogFC	FDR
TRINITY_DN4445_c0_g1_i2	-9,39	2,69E-06
TRINITY_DN1618_c0_g1_i10	-9,38	2,73E-06
TRINITY_DN4081_c0_g1_i1	-9,38	2,76E-06
TRINITY_DN2867_c0_g1_i2	-9,37	2,76E-06
TRINITY_DN2236_c0_g1_i1	-9,37	1,56E-08
TRINITY_DN2435_c0_g1_i2	-9,36	2,89E-06
TRINITY_DN1487_c0_g1_i5	-9,36	2,89E-06
TRINITY_DN4245_c0_g1_i2	-9,36	2,89E-06
TRINITY_DN2860_c0_g1_i3	-9,35	2,94E-06
TRINITY_DN95_c0_g1_i26	-9,34	3,10E-06
TRINITY_DN2783_c0_g1_i6	-9,33	3,15E-06
TRINITY_DN2580_c0_g1_i7	-9,32	3,18E-06
TRINITY_DN279_c0_g1_i1	-9,32	2,81E-06
TRINITY_DN1592_c2_g1_i1	-9,31	3,28E-06
TRINITY_DN605_c0_g1_i8	-9,31	3,32E-06
TRINITY_DN2660_c0_g1_i3	-9,31	3,32E-06
TRINITY_DN1247_c0_g1_i1	-9,27	3,62E-06
TRINITY_DN2305_c0_g1_i4	-9,27	3,67E-06
TRINITY_DN9078_c0_g1_i1	-9,26	3,74E-06
TRINITY_DN699_c0_g1_i11	-9,25	8,34E-07
TRINITY_DN1675_c0_g1_i6	-9,24	4,01E-06
TRINITY_DN3480_c1_g1_i9	-9,24	4,01E-06
TRINITY_DN1715_c0_g1_i2	-9,23	4,01E-06
TRINITY_DN2371_c0_g1_i4	-9,21	4,31E-06
TRINITY_DN2007_c0_g1_i9	-9,21	4,31E-06
TRINITY_DN4050_c0_g1_i42	-9,21	4,31E-06
TRINITY_DN4456_c0_g1_i1	-9,21	4,39E-06
TRINITY_DN2371_c0_g1_i16	-9,20	4,47E-06
TRINITY_DN3434_c0_g1_i1	-9,19	4,54E-06
TRINITY_DN3032_c0_g1_i2	-9,18	4,61E-06
TRINITY_DN6057_c3_g1_i2	-9,18	4,61E-06
TRINITY_DN2815_c0_g1_i4	-9,17	4,79E-06
TRINITY_DN3480_c1_g1_i2	-9,16	4,97E-06
TRINITY_DN1452_c0_g1_i7	-9,15	5,04E-06
TRINITY_DN4164_c0_g1_i3	-9,14	5,12E-06
TRINITY_DN2373_c0_g1_i8	-9,14	5,21E-06
TRINITY_DN3624_c0_g1_i1	-9,13	5,31E-06
TRINITY_DN3071_c1_g1_i3	-9,12	5,40E-06
TRINITY_DN2983_c0_g1_i2	-9,12	5,50E-06
TRINITY_DN1618_c0_g1_i21	-9,11	5,50E-06
TRINITY_DN3454_c0_g1_i1	-9,11	5,59E-06
TRINITY_DN433_c0_g1_i5	-9,11	5,59E-06

Trinity ID	LogFC	FDR
TRINITY_DN1765_c0_g1_i3	-9,10	5,81E-06
TRINITY_DN1140_c0_g1_i13	-9,09	5,81E-06
TRINITY_DN3480_c0_g1_i6	-9,08	6,15E-06
TRINITY_DN4414_c0_g1_i2	-9,07	6,15E-06
TRINITY_DN7695_c0_g1_i4	-9,07	6,28E-06
TRINITY_DN1149_c0_g1_i1	-9,04	6,68E-06
TRINITY_DN1243_c2_g1_i15	-9,03	2,38E-07
TRINITY_DN2815_c0_g1_i3	-9,02	7,12E-06
TRINITY_DN1393_c0_g1_i8	-9,01	3,45E-07
TRINITY_DN2402_c0_g1_i10	-9,00	7,58E-06
TRINITY_DN8088_c0_g1_i1	-8,99	7,71E-06
TRINITY_DN3660_c0_g1_i3	-8,99	1,08E-06
TRINITY_DN48_c1_g1_i7	-8,99	2,09E-08
TRINITY_DN3031_c0_g1_i1	-8,99	7,71E-06
TRINITY_DN546_c0_g1_i40	-8,99	7,84E-06
TRINITY_DN3508_c0_g1_i3	-8,99	7,84E-06
TRINITY_DN1039_c0_g1_i4	-8,98	7,84E-06
TRINITY_DN5050_c0_g1_i2	-8,97	8,19E-06
TRINITY_DN669_c1_g1_i2	-8,97	8,19E-06
TRINITY_DN4013_c0_g1_i3	-8,97	8,19E-06
TRINITY_DN156_c0_g1_i4	-8,96	8,34E-06
TRINITY_DN3071_c1_g1_i1	-8,96	2,03E-06
TRINITY_DN1579_c0_g1_i5	-8,96	8,52E-06
TRINITY_DN3624_c0_g1_i3	-8,95	8,52E-06
TRINITY_DN95_c0_g1_i11	-8,95	8,67E-06
TRINITY_DN1713_c0_g1_i4	-8,93	9,04E-06
TRINITY_DN4811_c0_g1_i2	-8,93	9,04E-06
TRINITY_DN2910_c0_g1_i5	-8,92	9,47E-06
TRINITY_DN1341_c0_g1_i2	-8,91	9,70E-06
TRINITY_DN2655_c0_g1_i7	-8,90	9,91E-06
TRINITY_DN3781_c0_g1_i2	-8,89	1,01E-05
TRINITY_DN95_c0_g1_i7	-8,88	1,04E-05
TRINITY_DN883_c4_g1_i5	-8,88	1,04E-05
TRINITY_DN1573_c0_g1_i7	-8,88	1,06E-05
TRINITY_DN556_c0_g1_i3	-8,87	1,08E-05
TRINITY_DN546_c0_g1_i24	-8,87	1,08E-05
TRINITY_DN6521_c0_g1_i2	-8,86	1,44E-06
TRINITY_DN2660_c0_g1_i5	-8,86	1,11E-05
TRINITY_DN1847_c0_g1_i3	-8,85	6,40E-07
TRINITY_DN4911_c0_g1_i1	-8,85	1,14E-05
TRINITY_DN1847_c0_g1_i8	-8,84	5,81E-07
TRINITY_DN73_c3_g1_i2	-8,83	1,19E-05

Trinity ID	LogFC	FDR
TRINITY_DN3182_c0_g1_i4	-8,83	1,22E-05
TRINITY_DN296_c3_g1_i2	-8,82	1,25E-05
TRINITY_DN4450_c0_g1_i3	-8,81	1,27E-05
TRINITY_DN3089_c0_g1_i2	-8,81	1,27E-05
TRINITY_DN937_c0_g1_i11	-8,80	1,31E-05
TRINITY_DN3190_c0_g1_i2	-8,75	1,53E-05
TRINITY_DN4290_c0_g1_i1	-8,72	1,60E-05
TRINITY_DN182_c0_g1_i5	-8,72	1,64E-05
TRINITY_DN2614_c0_g1_i5	-8,72	1,64E-05
TRINITY_DN4519_c0_g1_i6	-8,72	1,64E-05
TRINITY_DN1680_c0_g1_i5	-8,72	1,64E-05
TRINITY_DN511_c0_g2_i3	-8,70	1,71E-05
TRINITY_DN1767_c1_g1_i6	-8,70	1,64E-05
TRINITY_DN2706_c0_g1_i4	-8,70	2,34E-08
TRINITY_DN4276_c0_g1_i6	-8,70	1,71E-05
TRINITY_DN2654_c0_g1_i1	-8,69	1,71E-05
TRINITY_DN4703_c0_g1_i5	-8,68	1,81E-05
TRINITY_DN2946_c0_g1_i3	-8,67	1,85E-05
TRINITY_DN2_c0_g1_i6	-8,65	1,95E-05
TRINITY_DN2121_c0_g1_i5	-8,65	1,95E-05
TRINITY_DN4081_c0_g1_i10	-8,65	2,00E-05
TRINITY_DN1249_c0_g1_i1	-8,64	2,00E-05
TRINITY_DN1039_c0_g1_i3	-8,60	2,23E-05
TRINITY_DN3031_c0_g1_i4	-8,60	2,29E-05
TRINITY_DN2502_c0_g1_i11	-8,59	2,29E-05
TRINITY_DN3872_c0_g2_i2	-8,59	2,29E-05
TRINITY_DN95_c0_g1_i1	-8,59	2,35E-05
TRINITY_DN996_c1_g1_i5	-8,58	2,35E-05
TRINITY_DN4962_c0_g1_i1	-8,58	2,40E-05
TRINITY_DN2614_c0_g1_i10	-8,58	2,40E-05
TRINITY_DN108_c0_g1_i7	-8,58	4,77E-07
TRINITY_DN1249_c0_g1_i8	-8,57	2,40E-05
TRINITY_DN2665_c0_g1_i4	-8,57	2,46E-05
TRINITY_DN1767_c1_g1_i11	-8,55	2,53E-05
TRINITY_DN3027_c0_g1_i3	-8,55	2,53E-05
TRINITY_DN3484_c0_g1_i4	-8,52	2,77E-05
TRINITY_DN1195_c0_g1_i7	-8,51	2,86E-05
TRINITY_DN1196_c0_g1_i8	-8,50	3,07E-07
TRINITY_DN2138_c0_g1_i1	-8,49	3,03E-05
TRINITY_DN1680_c0_g1_i4	-8,49	3,03E-05
TRINITY_DN2599_c0_g3_i3	-8,48	3,11E-05
TRINITY_DN1084_c0_g1_i1	-8,48	3,11E-05

Trinity ID	LogFC	FDR
TRINITY_DN1982_c0_g1_i4	-8,47	3,21E-05
TRINITY_DN44_c0_g1_i16	-8,46	3,21E-05
TRINITY_DN1402_c0_g1_i4	-8,45	3,39E-05
TRINITY_DN2121_c0_g1_i1	-8,44	3,39E-05
TRINITY_DN3378_c0_g1_i4	-8,44	3,48E-05
TRINITY_DN483_c0_g1_i6	-8,43	1,10E-07
TRINITY_DN3980_c0_g1_i1	-8,40	3,82E-05
TRINITY_DN1285_c0_g1_i27	-8,34	2,89E-06
TRINITY_DN532_c0_g1_i27	-8,32	5,40E-07
TRINITY_DN1140_c0_g1_i2	-8,32	4,81E-05
TRINITY_DN2113_c1_g1_i1	-8,31	9,98E-08
TRINITY_DN490_c0_g1_i1	-8,29	3,51E-07
TRINITY_DN4842_c0_g1_i1	-8,28	1,64E-07
TRINITY_DN3519_c0_g2_i10	-8,26	5,47E-05
TRINITY_DN5096_c0_g1_i1	-8,24	5,88E-05
TRINITY_DN3533_c0_g1_i3	-8,18	7,03E-05
TRINITY_DN665_c0_g1_i15	-8,11	8,46E-05
TRINITY_DN640_c0_g1_i2	-8,10	3,16E-06
TRINITY_DN4575_c0_g1_i1	-8,08	1,19E-05
TRINITY_DN215_c1_g1_i1	-8,06	3,02E-07
TRINITY_DN2177_c0_g1_i5	-7,96	3,86E-07
TRINITY_DN2177_c0_g1_i27	-7,89	1,37E-06
TRINITY_DN167_c1_g1_i6	-7,80	7,51E-07
TRINITY_DN312_c0_g1_i12	-7,79	1,59E-06
TRINITY_DN831_c1_g1_i9	-7,79	1,68E-05
TRINITY_DN1287_c0_g1_i9	-7,78	1,91E-07
TRINITY_DN2261_c0_g1_i12	-7,77	4,14E-07
TRINITY_DN2277_c0_g1_i2	-7,61	4,95E-05
TRINITY_DN1717_c0_g1_i6	-7,61	2,91E-07
TRINITY_DN103_c3_g1_i7	-7,48	1,02E-06
TRINITY_DN2602_c0_g1_i2	-7,48	6,61E-07
TRINITY_DN194_c0_g1_i2	-7,46	3,79E-07
TRINITY_DN11337_c0_g1_i1	-7,44	2,03E-06
TRINITY_DN1285_c0_g1_i25	-7,43	4,95E-05
TRINITY_DN713_c0_g1_i4	-7,42	1,21E-06
TRINITY_DN2076_c0_g1_i2	-7,38	1,57E-05
TRINITY_DN1172_c0_g1_i2	-7,37	1,01E-04
TRINITY_DN789_c0_g1_i80	-7,36	9,30E-07
TRINITY_DN2032_c0_g1_i1	-7,33	1,62E-06
TRINITY_DN490_c0_g1_i21	-7,33	3,11E-05
TRINITY_DN4050_c0_g1_i25	-7,30	1,62E-06
TRINITY_DN3447_c0_g1_i3	-7,28	2,12E-06

Trinity ID	LogFC	FDR
TRINITY_DN503_c0_g1_i14	-7,27	7,21E-06
TRINITY_DN941_c0_g1_i3	-7,24	1,09E-06
TRINITY_DN214_c0_g1_i8	-7,21	2,09E-06
TRINITY_DN48_c1_g1_i5	-7,14	1,93E-06
TRINITY_DN640_c0_g1_i15	-7,11	1,10E-05
TRINITY_DN351_c0_g1_i9	-7,10	4,53E-05
TRINITY_DN154_c0_g1_i5	-7,05	1,21E-06
TRINITY_DN1784_c0_g1_i4	-7,04	2,03E-06
TRINITY_DN48_c1_g1_i1	-7,04	1,12E-06
TRINITY_DN2474_c0_g1_i9	-7,01	1,48E-06
TRINITY_DN4706_c1_g1_i1	-7,01	2,01E-06
TRINITY_DN4050_c0_g1_i45	-6,99	1,43E-06
TRINITY_DN12621_c0_g1_i1	-6,99	1,63E-06
TRINITY_DN242_c0_g1_i39	-6,95	1,48E-06
TRINITY_DN1974_c0_g1_i5	-6,91	2,08E-05
TRINITY_DN2371_c0_g1_i19	-6,89	3,16E-06
TRINITY_DN164_c0_g1_i2	-6,87	2,33E-06
TRINITY_DN4_c1_g1_i7	-6,82	8,65E-06
TRINITY_DN1405_c0_g1_i1	-6,80	5,07E-06
TRINITY_DN1661_c0_g1_i3	-6,79	4,12E-06
TRINITY_DN12767_c0_g1_i1	-6,78	2,05E-05
TRINITY_DN242_c0_g1_i28	-6,76	3,20E-06
TRINITY_DN626_c3_g1_i3	-6,71	5,60E-06
TRINITY_DN823_c0_g1_i2	-6,69	1,36E-05
TRINITY_DN3178_c0_g1_i3	-6,68	3,28E-06
TRINITY_DN665_c0_g1_i62	-6,64	7,63E-06
TRINITY_DN90_c0_g1_i1	-6,62	2,21E-05
TRINITY_DN154_c0_g1_i7	-6,61	3,60E-06
TRINITY_DN640_c0_g1_i17	-6,61	6,15E-06
TRINITY_DN661_c0_g1_i80	-6,61	8,80E-06
TRINITY_DN7799_c0_g1_i1	-6,59	1,93E-05
TRINITY_DN1124_c0_g1_i4	-6,56	1,27E-05
TRINITY_DN1492_c0_g1_i2	-6,54	1,62E-05
TRINITY_DN13_c0_g1_i17	-6,54	3,47E-05
TRINITY_DN1247_c0_g1_i7	-6,54	4,01E-06
TRINITY_DN1648_c0_g1_i2	-6,53	8,84E-06
TRINITY_DN1814_c0_g1_i2	-6,52	5,42E-05
TRINITY_DN5069_c0_g1_i1	-6,51	4,14E-06
TRINITY_DN2549_c0_g1_i6	-6,51	5,94E-06
TRINITY_DN1285_c0_g1_i39	-6,47	4,95E-05
TRINITY_DN605_c0_g1_i10	-6,47	7,32E-06
TRINITY_DN4050_c0_g1_i3	-6,47	5,04E-06

Trinity ID	LogFC	FDR
TRINITY_DN447_c0_g1_i4	-6,42	5,31E-06
TRINITY_DN1285_c0_g1_i14	-6,39	1,27E-05
TRINITY_DN624_c0_g1_i4	-6,39	1,18E-05
TRINITY_DN4637_c0_g1_i3	-6,37	8,79E-06
TRINITY_DN754_c1_g2_i4	-6,34	4,61E-05
TRINITY_DN1694_c0_g1_i6	-6,34	6,37E-06
TRINITY_DN759_c0_g1_i15	-6,33	6,56E-06
TRINITY_DN224_c1_g1_i7	-6,32	1,25E-04
TRINITY_DN386_c0_g1_i1	-6,32	6,80E-06
TRINITY_DN1492_c0_g1_i12	-6,29	4,89E-05
TRINITY_DN4050_c0_g1_i53	-6,26	1,26E-05
TRINITY_DN50_c0_g1_i16	-6,25	8,00E-05
TRINITY_DN5658_c0_g1_i3	-6,24	2,06E-05
TRINITY_DN3480_c1_g1_i6	-6,24	3,39E-05
TRINITY_DN385_c0_g1_i5	-6,23	1,64E-05
TRINITY_DN224_c1_g1_i4	-6,23	4,95E-05
TRINITY_DN1709_c0_g1_i10	-6,21	5,05E-05
TRINITY_DN108_c0_g1_i5	-6,21	1,27E-05
TRINITY_DN2582_c1_g1_i1	-6,19	1,83E-05
TRINITY_DN4494_c1_g1_i1	-6,18	9,83E-06
TRINITY_DN703_c0_g1_i2	-6,18	2,28E-05
TRINITY_DN1228_c0_g1_i44	-6,16	2,40E-05
TRINITY_DN1837_c0_g1_i2	-6,14	1,58E-05
TRINITY_DN282_c0_g1_i3	-6,12	1,46E-05
TRINITY_DN1177_c0_g1_i5	-6,12	1,75E-04
TRINITY_DN2261_c0_g1_i13	-6,10	1,59E-05
TRINITY_DN13_c0_g1_i18	-6,08	1,71E-05
TRINITY_DN2195_c0_g2_i2	-6,07	3,29E-05
TRINITY_DN626_c2_g1_i4	-6,06	1,64E-05
TRINITY_DN980_c0_g1_i19	-6,05	3,29E-05
TRINITY_DN4050_c0_g1_i20	-6,05	8,46E-05
TRINITY_DN388_c0_g1_i11	-6,05	4,02E-05
TRINITY_DN564_c0_g1_i4	-6,04	2,52E-05
TRINITY_DN1333_c0_g1_i5	-6,03	3,20E-05
TRINITY_DN1191_c0_g1_i6	-6,01	3,82E-05
TRINITY_DN837_c0_g1_i1	-5,98	1,80E-05
TRINITY_DN1572_c0_g1_i1	-5,98	2,30E-05
TRINITY_DN12668_c0_g1_i1	-5,98	2,39E-04
TRINITY_DN5437_c0_g1_i1	-5,97	1,76E-05
TRINITY_DN1648_c0_g1_i10	-5,97	2,57E-05
TRINITY_DN5022_c0_g1_i1	-5,97	1,80E-05
TRINITY_DN8440_c0_g1_i2	-5,95	2,96E-05

Trinity ID	LogFC	FDR
TRINITY_DN1228_c0_g1_i5	-5,95	3,38E-05
TRINITY_DN4027_c0_g2_i2	-5,94	4,04E-05
TRINITY_DN1771_c0_g1_i2	-5,93	7,39E-05
TRINITY_DN534_c0_g1_i20	-5,93	2,04E-05
TRINITY_DN1489_c0_g1_i3	-5,91	2,42E-05
TRINITY_DN214_c0_g1_i9	-5,90	5,59E-05
TRINITY_DN945_c0_g1_i11	-5,89	1,40E-04
TRINITY_DN1007_c0_g1_i21	-5,89	2,24E-04
TRINITY_DN2783_c0_g1_i11	-5,87	7,19E-05
TRINITY_DN13_c0_g1_i6	-5,86	2,34E-05
TRINITY_DN3276_c0_g1_i1	-5,86	6,37E-05
TRINITY_DN626_c2_g1_i2	-5,86	6,22E-05
TRINITY_DN980_c0_g1_i57	-5,85	3,27E-05
TRINITY_DN153_c0_g1_i16	-5,85	1,42E-04
TRINITY_DN2056_c1_g1_i4	-5,83	5,74E-05
TRINITY_DN661_c0_g1_i7	-5,83	3,34E-05
TRINITY_DN295_c1_g1_i2	-5,82	2,84E-04
TRINITY_DN837_c0_g1_i2	-5,82	2,63E-05
TRINITY_DN2076_c0_g1_i1	-5,80	3,00E-05
TRINITY_DN2980_c0_g1_i2	-5,79	5,08E-05
TRINITY_DN201_c0_g1_i2	-5,78	3,61E-05
TRINITY_DN215_c1_g1_i5	-5,76	4,72E-05
TRINITY_DN2356_c0_g1_i3	-5,75	3,77E-05
TRINITY_DN534_c0_g1_i6	-5,75	3,54E-05
TRINITY_DN214_c0_g1_i5	-5,72	5,24E-05
TRINITY_DN1717_c0_g1_i5	-5,71	3,63E-05
TRINITY_DN2001_c0_g1_i1	-5,71	1,57E-04
TRINITY_DN1391_c0_g1_i1	-5,71	4,03E-05
TRINITY_DN2328_c0_g1_i2	-5,70	5,38E-05
TRINITY_DN560_c0_g1_i5	-5,70	5,87E-05
TRINITY_DN1140_c0_g1_i5	-5,68	1,29E-04
TRINITY_DN638_c0_g2_i7	-5,68	1,14E-04
TRINITY_DN501_c0_g1_i3	-5,63	3,67E-04
TRINITY_DN941_c0_g1_i1	-5,59	7,81E-05
TRINITY_DN945_c0_g1_i12	-5,59	1,56E-04
TRINITY_DN622_c0_g1_i4	-5,59	5,24E-05
TRINITY_DN1441_c1_g1_i10	-5,58	6,19E-05
TRINITY_DN386_c0_g1_i2	-5,58	5,56E-05
TRINITY_DN3092_c0_g1_i7	-5,56	6,94E-05
TRINITY_DN528_c0_g1_i17	-5,55	4,33E-04
TRINITY_DN983_c0_g2_i8	-5,55	7,02E-05
TRINITY_DN1776_c3_g1_i2	-5,53	8,12E-05

Trinity ID	LogFC	FDR
TRINITY_DN182_c0_g1_i11	-5,52	8,60E-05
TRINITY_DN1690_c0_g1_i1	-5,51	1,35E-04
TRINITY_DN2606_c0_g1_i3	-5,50	6,61E-05
TRINITY_DN4704_c0_g1_i1	-5,50	1,88E-04
TRINITY_DN190_c0_g1_i7	-5,49	7,55E-05
TRINITY_DN640_c0_g1_i3	-5,48	7,04E-05
TRINITY_DN1717_c0_g1_i10	-5,48	7,45E-05
TRINITY_DN2380_c1_g1_i3	-5,47	1,78E-04
TRINITY_DN1000_c0_g1_i4	-5,45	2,94E-04
TRINITY_DN154_c0_g1_i16	-5,45	7,98E-05
TRINITY_DN733_c0_g1_i12	-5,44	1,02E-04
TRINITY_DN0_c0_g2_i2	-5,42	8,15E-05
TRINITY_DN4081_c0_g1_i7	-5,40	1,27E-04
TRINITY_DN583_c0_g1_i2	-5,38	8,99E-05
TRINITY_DN385_c0_g1_i8	-5,38	2,88E-04
TRINITY_DN583_c0_g1_i8	-5,37	9,29E-05
TRINITY_DN39_c0_g1_i10	-5,37	1,18E-04
TRINITY_DN2815_c0_g1_i2	-5,37	1,41E-04
TRINITY_DN4820_c0_g1_i1	-5,36	2,03E-04
TRINITY_DN996_c1_g1_i7	-5,36	1,14E-04
TRINITY_DN1929_c0_g1_i5	-5,35	1,81E-04
TRINITY_DN1113_c0_g1_i2	-5,32	1,73E-04
TRINITY_DN3142_c0_g1_i1	-5,31	1,26E-04
TRINITY_DN1140_c0_g1_i6	-5,31	2,56E-04
TRINITY_DN1196_c0_g1_i7	-5,30	1,31E-04
TRINITY_DN1022_c0_g1_i4	-5,26	1,83E-04
TRINITY_DN941_c1_g1_i2	-5,26	1,78E-04
TRINITY_DN2665_c0_g1_i3	-5,26	9,75E-04
TRINITY_DN2159_c0_g1_i1	-5,25	1,73E-04
TRINITY_DN2082_c0_g1_i4	-5,25	7,98E-04
TRINITY_DN13_c0_g1_i53	-5,25	2,69E-04
TRINITY_DN351_c0_g1_i8	-5,24	7,40E-04
TRINITY_DN206_c0_g1_i19	-5,22	3,30E-04
TRINITY_DN9223_c0_g1_i1	-5,22	1,55E-04
TRINITY_DN1840_c0_g1_i1	-5,22	2,82E-04
TRINITY_DN2023_c0_g1_i2	-5,21	2,01E-04
TRINITY_DN3208_c0_g1_i1	-5,20	1,59E-04
TRINITY_DN528_c0_g1_i11	-5,20	1,71E-04
TRINITY_DN5375_c0_g1_i1	-5,19	1,61E-04
TRINITY_DN534_c0_g1_i7	-5,19	1,71E-04
TRINITY_DN5145_c0_g2_i2	-5,19	2,17E-04
TRINITY_DN1661_c0_g1_i4	-5,19	4,63E-04

Trinity ID	LogFC	FDR
TRINITY_DN11870_c0_g1_i1	-5,18	1,59E-04
TRINITY_DN3376_c0_g1_i2	-5,17	2,96E-04
TRINITY_DN153_c0_g1_i11	-5,17	1,65E-04
TRINITY_DN432_c0_g1_i8	-5,15	4,33E-04
TRINITY_DN1899_c0_g1_i2	-5,14	2,47E-04
TRINITY_DN640_c0_g1_i1	-5,14	1,83E-04
TRINITY_DN0_c0_g1_i3	-5,13	2,14E-04
TRINITY_DN2580_c0_g1_i4	-5,13	2,28E-04
TRINITY_DN18_c0_g1_i22	-5,13	2,48E-04
TRINITY_DN411_c0_g1_i56	-5,10	2,79E-04
TRINITY_DN28_c1_g1_i14	-5,10	2,41E-04
TRINITY_DN13_c0_g1_i7	-5,09	3,36E-04
TRINITY_DN306_c0_g1_i14	-5,09	4,25E-04
TRINITY_DN2282_c0_g1_i2	-5,09	3,21E-04
TRINITY_DN1971_c0_g1_i2	-5,08	5,98E-04
TRINITY_DN850_c0_g1_i1	-5,08	2,74E-04
TRINITY_DN892_c0_g1_i6	-5,08	2,69E-04
TRINITY_DN4326_c0_g1_i2	-5,08	2,56E-04
TRINITY_DN1675_c0_g1_i4	-5,07	7,58E-04
TRINITY_DN800_c0_g1_i6	-5,05	7,27E-04
TRINITY_DN2690_c0_g1_i12	-5,03	1,62E-03
TRINITY_DN1275_c0_g1_i11	-5,02	2,90E-04
TRINITY_DN1441_c1_g1_i9	-5,02	2,47E-04
TRINITY_DN1039_c0_g1_i1	-5,02	7,05E-04
TRINITY_DN462_c1_g2_i1	-5,01	2,54E-04
TRINITY_DN1978_c0_g1_i3	-4,99	9,87E-04
TRINITY_DN18_c0_g1_i25	-4,99	5,63E-04
TRINITY_DN11334_c0_g1_i1	-4,98	3,24E-04
TRINITY_DN2203_c0_g1_i4	-4,96	2,95E-04
TRINITY_DN4367_c0_g1_i1	-4,96	3,17E-04
TRINITY_DN1694_c0_g1_i4	-4,96	3,48E-04
TRINITY_DN4577_c0_g2_i3	-4,96	4,96E-04
TRINITY_DN2347_c0_g1_i5	-4,94	1,19E-03
TRINITY_DN2690_c0_g1_i8	-4,92	3,67E-04
TRINITY_DN1022_c0_g1_i5	-4,92	3,47E-04
TRINITY_DN1929_c0_g1_i6	-4,92	6,14E-04
TRINITY_DN996_c1_g1_i2	-4,91	3,47E-04
TRINITY_DN170_c0_g1_i40	-4,91	8,07E-04
TRINITY_DN1405_c0_g1_i2	-4,90	3,50E-04
TRINITY_DN1253_c0_g1_i2	-4,90	6,04E-04
TRINITY_DN738_c0_g1_i2	-4,89	5,66E-04
TRINITY_DN13_c0_g2_i9	-4,89	3,64E-04

Trinity ID	LogFC	FDR
TRINITY_DN12654_c0_g1_i1	-4,86	4,06E-04
TRINITY_DN154_c0_g1_i10	-4,86	4,14E-04
TRINITY_DN3496_c0_g1_i3	-4,86	8,92E-04
TRINITY_DN1487_c0_g1_i2	-4,86	4,85E-04
TRINITY_DN789_c0_g1_i14	-4,86	5,15E-04
TRINITY_DN519_c0_g1_i7	-4,86	8,46E-04
TRINITY_DN279_c0_g1_i3	-4,86	5,67E-04
TRINITY_DN665_c0_g1_i7	-4,86	2,38E-03
TRINITY_DN1661_c0_g1_i25	-4,85	4,84E-04
TRINITY_DN1287_c0_g1_i4	-4,85	4,60E-04
TRINITY_DN4731_c0_g1_i1	-4,84	4,24E-04
TRINITY_DN3039_c0_g1_i1	-4,84	5,90E-04
TRINITY_DN449_c1_g1_i36	-4,83	1,80E-03
TRINITY_DN1419_c0_g1_i1	-4,83	5,52E-04
TRINITY_DN640_c0_g1_i7	-4,82	9,59E-04
TRINITY_DN62_c0_g1_i4	-4,82	5,98E-04
TRINITY_DN7427_c0_g1_i2	-4,82	7,16E-04
TRINITY_DN665_c0_g1_i27	-4,82	7,16E-04
TRINITY_DN2126_c1_g1_i2	-4,82	4,98E-04
TRINITY_DN683_c0_g1_i12	-4,81	6,27E-04
TRINITY_DN1615_c0_g1_i4	-4,81	3,22E-03
TRINITY_DN1196_c0_g1_i4	-4,81	5,05E-04
TRINITY_DN986_c0_g1_i7	-4,81	6,17E-04
TRINITY_DN2877_c0_g1_i1	-4,80	5,63E-04
TRINITY_DN412_c0_g1_i4	-4,80	4,55E-04
TRINITY_DN570_c0_g1_i1	-4,80	8,63E-04
TRINITY_DN853_c0_g1_i2	-4,80	1,33E-03
TRINITY_DN224_c1_g1_i15	-4,80	7,23E-04
TRINITY_DN13_c0_g1_i63	-4,80	5,12E-04
TRINITY_DN164_c0_g1_i3	-4,78	5,11E-04
TRINITY_DN1777_c2_g1_i1	-4,77	8,22E-04
TRINITY_DN2262_c0_g1_i8	-4,77	7,00E-04
TRINITY_DN4366_c0_g1_i1	-4,76	1,39E-03
TRINITY_DN39_c0_g1_i14	-4,76	6,86E-04
TRINITY_DN556_c0_g1_i14	-4,75	5,39E-04
TRINITY_DN945_c0_g1_i5	-4,75	1,00E-03
TRINITY_DN3137_c0_g1_i5	-4,75	1,24E-03
TRINITY_DN560_c0_g1_i12	-4,74	5,38E-04
TRINITY_DN718_c0_g1_i12	-4,74	5,98E-04
TRINITY_DN1268_c0_g1_i2	-4,74	6,40E-04
TRINITY_DN4844_c0_g1_i1	-4,74	1,45E-03
TRINITY_DN665_c0_g1_i6	-4,74	7,76E-04

Trinity ID	LogFC	FDR
TRINITY_DN1276_c0_g1_i2	-4,74	1,28E-03
TRINITY_DN1377_c0_g2_i8	-4,73	6,86E-04
TRINITY_DN4830_c0_g2_i1	-4,72	8,88E-04
TRINITY_DN2126_c1_g1_i1	-4,72	5,67E-04
TRINITY_DN5658_c0_g1_i4	-4,72	5,90E-04
TRINITY_DN583_c0_g1_i7	-4,72	1,87E-03
TRINITY_DN95_c0_g1_i9	-4,71	6,22E-04
TRINITY_DN1916_c0_g2_i14	-4,71	1,07E-03
TRINITY_DN3787_c1_g1_i4	-4,71	1,27E-03
TRINITY_DN2907_c0_g1_i1	-4,70	6,35E-04
TRINITY_DN528_c0_g1_i5	-4,70	8,10E-04
TRINITY_DN1254_c0_g1_i2	-4,70	6,14E-04
TRINITY_DN2690_c0_g3_i2	-4,70	6,22E-04
TRINITY_DN217_c0_g1_i16	-4,69	6,22E-04
TRINITY_DN835_c0_g1_i5	-4,69	8,76E-04
TRINITY_DN3188_c0_g1_i4	-4,69	8,10E-04
TRINITY_DN2690_c0_g1_i2	-4,69	9,69E-04
TRINITY_DN638_c0_g2_i17	-4,68	1,08E-03
TRINITY_DN1045_c0_g1_i2	-4,68	7,07E-04
TRINITY_DN590_c0_g1_i9	-4,68	6,37E-04
TRINITY_DN255_c0_g1_i5	-4,67	1,35E-03
TRINITY_DN388_c0_g1_i8	-4,67	1,32E-03
TRINITY_DN1848_c0_g1_i2	-4,67	8,05E-04
TRINITY_DN3216_c1_g1_i3	-4,67	7,02E-04
TRINITY_DN556_c0_g1_i4	-4,65	6,85E-04
TRINITY_DN426_c0_g1_i2	-4,64	1,05E-03
TRINITY_DN149_c0_g1_i11	-4,64	1,09E-03
TRINITY_DN638_c0_g2_i35	-4,64	7,08E-04
TRINITY_DN1184_c0_g1_i10	-4,63	1,14E-03
TRINITY_DN2718_c0_g1_i3	-4,63	9,37E-04
TRINITY_DN1016_c0_g1_i4	-4,63	1,11E-03
TRINITY_DN4474_c0_g2_i1	-4,63	9,83E-04
TRINITY_DN2304_c1_g1_i2	-4,61	8,07E-04
TRINITY_DN2304_c1_g1_i3	-4,61	1,35E-03
TRINITY_DN90_c0_g1_i2	-4,61	8,81E-04
TRINITY_DN144_c0_g1_i2	-4,60	7,81E-04
TRINITY_DN9110_c0_g1_i1	-4,60	3,44E-03
TRINITY_DN2371_c0_g1_i9	-4,60	8,88E-04
TRINITY_DN215_c1_g1_i7	-4,59	9,11E-04
TRINITY_DN893_c0_g1_i3	-4,58	9,27E-04
TRINITY_DN560_c0_g1_i1	-4,58	3,02E-03
TRINITY_DN4438_c0_g1_i3	-4,58	1,47E-03

Trinity ID	LogFC	FDR
TRINITY_DN36_c0_g2_i2	-4,58	1,09E-03
TRINITY_DN1078_c1_g1_i2	-4,57	1,71E-03
TRINITY_DN312_c0_g1_i9	-4,57	1,60E-03
TRINITY_DN18_c0_g1_i16	-4,57	9,50E-04
TRINITY_DN217_c0_g1_i26	-4,56	1,46E-03
TRINITY_DN23_c2_g1_i1	-4,56	8,88E-04
TRINITY_DN4013_c0_g1_i1	-4,55	8,99E-04
TRINITY_DN3646_c0_g1_i2	-4,55	9,09E-04
TRINITY_DN240_c1_g1_i1	-4,55	9,07E-04
TRINITY_DN1333_c0_g1_i1	-4,55	1,34E-03
TRINITY_DN1777_c2_g1_i4	-4,54	1,24E-03
TRINITY_DN306_c1_g1_i6	-4,54	9,07E-04
TRINITY_DN154_c0_g1_i13	-4,54	9,43E-04
TRINITY_DN610_c0_g1_i31	-4,54	9,20E-04
TRINITY_DN661_c0_g1_i70	-4,54	1,14E-03
TRINITY_DN661_c0_g1_i30	-4,54	9,17E-04
TRINITY_DN3466_c0_g1_i4	-4,53	2,51E-03
TRINITY_DN2063_c0_g1_i13	-4,53	2,02E-03
TRINITY_DN18_c0_g1_i3	-4,53	1,14E-03
TRINITY_DN3201_c0_g1_i1	-4,53	1,49E-03
TRINITY_DN2644_c0_g1_i1	-4,52	1,36E-03
TRINITY_DN1302_c0_g1_i7	-4,51	1,05E-03
TRINITY_DN1403_c0_g1_i10	-4,51	3,24E-03
TRINITY_DN362_c0_g1_i12	-4,51	1,05E-03
TRINITY_DN1606_c0_g1_i1	-4,50	1,47E-03
TRINITY_DN5993_c0_g1_i1	-4,50	1,23E-03
TRINITY_DN3089_c0_g1_i1	-4,50	1,12E-03
TRINITY_DN1879_c3_g1_i2	-4,49	1,05E-03
TRINITY_DN10543_c0_g1_i1	-4,49	1,06E-03
TRINITY_DN306_c1_g1_i4	-4,48	1,08E-03
TRINITY_DN1969_c0_g2_i3	-4,48	1,42E-03
TRINITY_DN638_c0_g2_i14	-4,48	1,07E-03
TRINITY_DN1450_c0_g1_i8	-4,48	1,19E-03
TRINITY_DN388_c0_g1_i2	-4,47	1,31E-03
TRINITY_DN1452_c0_g1_i11	-4,47	1,15E-03
TRINITY_DN1595_c0_g1_i7	-4,46	2,53E-03
TRINITY_DN3466_c0_g1_i1	-4,46	1,97E-03
TRINITY_DN2663_c0_g1_i4	-4,46	1,31E-03
TRINITY_DN12649_c0_g1_i1	-4,45	2,46E-03
TRINITY_DN298_c0_g1_i11	-4,45	1,78E-03
TRINITY_DN7552_c0_g1_i10	-4,45	2,51E-03
TRINITY_DN214_c0_g1_i6	-4,45	1,15E-03

Trinity ID	LogFC	FDR
TRINITY_DN3013_c1_g1_i4	-4,44	1,23E-03
TRINITY_DN863_c4_g1_i1	-4,44	1,16E-03
TRINITY_DN3703_c0_g1_i1	-4,44	1,23E-03
TRINITY_DN1940_c0_g1_i2	-4,44	1,35E-03
TRINITY_DN1805_c0_g1_i6	-4,44	1,50E-03
TRINITY_DN1240_c0_g1_i1	-4,43	1,45E-03
TRINITY_DN13_c0_g2_i8	-4,43	1,32E-03
TRINITY_DN1278_c0_g1_i2	-4,42	1,32E-03
TRINITY_DN2416_c0_g1_i4	-4,42	1,65E-03
TRINITY_DN912_c0_g1_i8	-4,42	2,53E-03
TRINITY_DN946_c1_g1_i1	-4,41	1,28E-03
TRINITY_DN693_c0_g1_i1	-4,41	1,60E-03
TRINITY_DN662_c0_g1_i3	-4,40	1,35E-03
TRINITY_DN2758_c0_g1_i4	-4,40	1,42E-03
TRINITY_DN1452_c0_g1_i5	-4,40	3,09E-03
TRINITY_DN1243_c2_g1_i4	-4,40	1,78E-03
TRINITY_DN624_c0_g1_i1	-4,39	2,68E-03
TRINITY_DN3706_c0_g1_i2	-4,39	2,40E-03
TRINITY_DN368_c1_g1_i2	-4,39	1,50E-03
TRINITY_DN22_c0_g1_i6	-4,39	1,42E-03
TRINITY_DN2874_c0_g1_i1	-4,39	2,00E-03
TRINITY_DN4149_c0_g1_i1	-4,39	1,54E-03
TRINITY_DN560_c0_g1_i13	-4,39	1,43E-03
TRINITY_DN1217_c0_g1_i10	-4,39	1,53E-03
TRINITY_DN1969_c0_g2_i2	-4,37	1,38E-03
TRINITY_DN1548_c0_g1_i10	-4,37	1,53E-03
TRINITY_DN3521_c0_g1_i1	-4,37	1,89E-03
TRINITY_DN167_c1_g1_i30	-4,36	3,59E-03
TRINITY_DN767_c0_g1_i6	-4,36	1,45E-03
TRINITY_DN13_c0_g1_i34	-4,35	2,07E-03
TRINITY_DN3566_c0_g1_i1	-4,34	2,30E-03
TRINITY_DN509_c3_g1_i2	-4,34	2,47E-03
TRINITY_DN3383_c2_g1_i1	-4,34	4,14E-03
TRINITY_DN1299_c1_g1_i1	-4,34	1,57E-03
TRINITY_DN503_c0_g1_i1	-4,34	1,78E-03
TRINITY_DN3032_c0_g1_i1	-4,33	1,95E-03
TRINITY_DN1225_c0_g1_i7	-4,33	1,92E-03
TRINITY_DN2146_c0_g1_i2	-4,33	1,61E-03
TRINITY_DN509_c0_g1_i3	-4,33	1,74E-03
TRINITY_DN5526_c0_g1_i9	-4,33	1,62E-03
TRINITY_DN1944_c0_g1_i1	-4,33	1,71E-03
TRINITY_DN153_c0_g1_i18	-4,32	1,71E-03

Trinity ID	LogFC	FDR
TRINITY_DN167_c1_g1_i32	-4,32	2,21E-03
TRINITY_DN2835_c0_g1_i2	-4,32	2,47E-03
TRINITY_DN2184_c0_g1_i1	-4,32	1,78E-03
TRINITY_DN1958_c1_g1_i3	-4,31	3,15E-03
TRINITY_DN231_c0_g2_i9	-4,31	4,22E-03
TRINITY_DN754_c1_g1_i2	-4,31	1,65E-03
TRINITY_DN292_c1_g1_i1	-4,30	2,15E-03
TRINITY_DN1241_c0_g1_i2	-4,30	2,36E-03
TRINITY_DN1861_c0_g1_i4	-4,30	2,41E-03
TRINITY_DN154_c0_g1_i6	-4,30	1,70E-03
TRINITY_DN447_c0_g1_i7	-4,30	1,95E-03
TRINITY_DN548_c0_g1_i1	-4,29	1,70E-03
TRINITY_DN3551_c0_g1_i2	-4,29	2,01E-03
TRINITY_DN835_c0_g1_i6	-4,29	2,83E-03
TRINITY_DN2256_c0_g1_i1	-4,28	1,90E-03
TRINITY_DN738_c0_g1_i1	-4,27	1,87E-03
TRINITY_DN5435_c0_g1_i1	-4,27	3,12E-03
TRINITY_DN2196_c0_g1_i3	-4,27	1,83E-03
TRINITY_DN2056_c1_g1_i10	-4,26	2,69E-03
TRINITY_DN2876_c0_g1_i5	-4,26	1,94E-03
TRINITY_DN323_c0_g1_i12	-4,25	1,90E-03
TRINITY_DN640_c0_g1_i9	-4,25	2,01E-03
TRINITY_DN530_c0_g1_i26	-4,25	2,03E-03
TRINITY_DN1489_c0_g1_i2	-4,24	1,95E-03
TRINITY_DN8247_c0_g1_i1	-4,24	3,20E-03
TRINITY_DN3149_c0_g1_i6	-4,23	2,05E-03
TRINITY_DN1899_c0_g1_i4	-4,23	2,02E-03
TRINITY_DN179_c0_g1_i7	-4,23	2,05E-03
TRINITY_DN13_c0_g1_i25	-4,22	2,06E-03
TRINITY_DN2416_c0_g1_i1	-4,22	2,06E-03
TRINITY_DN341_c2_g1_i1	-4,21	2,08E-03
TRINITY_DN201_c0_g1_i5	-4,21	4,22E-03
TRINITY_DN3411_c0_g1_i1	-4,21	3,35E-03
TRINITY_DN170_c0_g1_i22	-4,20	3,97E-03
TRINITY_DN509_c0_g1_i11	-4,20	4,86E-03
TRINITY_DN1940_c0_g1_i3	-4,20	4,16E-03
TRINITY_DN18_c0_g1_i2	-4,20	2,71E-03
TRINITY_DN3519_c0_g2_i9	-4,20	2,30E-03
TRINITY_DN2203_c0_g1_i1	-4,19	2,32E-03
TRINITY_DN206_c0_g1_i17	-4,19	3,58E-03
TRINITY_DN1727_c0_g1_i2	-4,19	4,76E-03
TRINITY_DN1979_c0_g1_i8	-4,19	2,94E-03

Trinity ID	LogFC	FDR
TRINITY_DN1979_c0_g1_i3	-4,18	5,58E-03
TRINITY_DN583_c0_g1_i9	-4,18	2,36E-03
TRINITY_DN2063_c0_g1_i5	-4,18	2,36E-03
TRINITY_DN767_c0_g1_i3	-4,18	2,39E-03
TRINITY_DN437_c0_g1_i27	-4,18	2,60E-03
TRINITY_DN2247_c0_g1_i3	-4,17	4,04E-03
TRINITY_DN4575_c0_g1_i2	-4,17	2,50E-03
TRINITY_DN13_c0_g1_i32	-4,16	2,59E-03
TRINITY_DN9329_c0_g1_i1	-4,15	5,47E-03
TRINITY_DN2744_c0_g1_i1	-4,15	3,71E-03
TRINITY_DN150_c0_g2_i4	-4,15	4,35E-03
TRINITY_DN1184_c0_g1_i7	-4,15	2,68E-03
TRINITY_DN1450_c0_g1_i2	-4,14	4,34E-03
TRINITY_DN640_c0_g1_i14	-4,13	3,75E-03
TRINITY_DN800_c0_g1_i1	-4,13	3,87E-03
TRINITY_DN911_c1_g1_i1	-4,12	2,70E-03
TRINITY_DN892_c0_g1_i3	-4,12	2,57E-03
TRINITY_DN3188_c0_g1_i1	-4,12	2,60E-03
TRINITY_DN2687_c0_g1_i3	-4,12	3,05E-03
TRINITY_DN1609_c0_g1_i1	-4,12	5,04E-03
TRINITY_DN8707_c0_g1_i1	-4,12	2,91E-03
TRINITY_DN774_c0_g1_i6	-4,11	5,12E-03
TRINITY_DN4562_c0_g2_i1	-4,11	4,52E-03
TRINITY_DN249_c0_g1_i10	-4,11	6,76E-03
TRINITY_DN632_c0_g1_i8	-4,11	4,36E-03
TRINITY_DN5044_c0_g1_i1	-4,11	2,68E-03
TRINITY_DN853_c0_g1_i4	-4,10	2,78E-03
TRINITY_DN95_c0_g1_i6	-4,10	3,88E-03
TRINITY_DN3783_c0_g1_i8	-4,10	3,89E-03
TRINITY_DN622_c0_g1_i3	-4,10	2,73E-03
TRINITY_DN0_c0_g2_i3	-4,09	2,95E-03
TRINITY_DN1140_c0_g1_i15	-4,09	3,74E-03
TRINITY_DN8620_c1_g1_i1	-4,09	3,25E-03
TRINITY_DN93_c0_g1_i32	-4,09	2,91E-03
TRINITY_DN3623_c0_g1_i2	-4,08	4,75E-03
TRINITY_DN1215_c5_g1_i1	-4,08	2,89E-03
TRINITY_DN2584_c0_g1_i1	-4,08	3,23E-03
TRINITY_DN528_c0_g1_i14	-4,08	2,99E-03
TRINITY_DN583_c0_g1_i4	-4,07	2,91E-03
TRINITY_DN4408_c0_g1_i4	-4,07	3,47E-03
TRINITY_DN665_c0_g1_i37	-4,07	4,07E-03
TRINITY_DN326_c0_g1_i3	-4,07	2,95E-03

Trinity ID	LogFC	FDR
TRINITY_DN912_c0_g1_i3	-4,07	3,16E-03
TRINITY_DN48_c0_g1_i1	-4,07	2,99E-03
TRINITY_DN2045_c0_g1_i4	-4,06	3,62E-03
TRINITY_DN1940_c0_g1_i1	-4,06	3,43E-03
TRINITY_DN411_c0_g1_i53	-4,06	3,78E-03
TRINITY_DN4474_c0_g1_i1	-4,05	3,08E-03
TRINITY_DN353_c0_g1_i8	-4,05	3,83E-03
TRINITY_DN682_c0_g3_i8	-4,05	3,05E-03
TRINITY_DN652_c0_g1_i2	-4,05	3,80E-03
TRINITY_DN640_c0_g1_i8	-4,05	3,17E-03
TRINITY_DN721_c0_g1_i5	-4,05	4,36E-03
TRINITY_DN665_c0_g1_i29	-4,05	3,22E-03
TRINITY_DN3533_c0_g1_i5	-4,05	7,40E-03
TRINITY_DN5196_c0_g1_i1	-4,05	3,25E-03
TRINITY_DN13_c0_g1_i11	-4,05	3,20E-03
TRINITY_DN893_c0_g1_i7	-4,04	3,92E-03
TRINITY_DN270_c0_g1_i16	-4,04	3,87E-03
TRINITY_DN1548_c0_g1_i5	-4,04	4,45E-03
TRINITY_DN1709_c0_g1_i3	-4,04	3,38E-03
TRINITY_DN1405_c0_g1_i6	-4,04	3,89E-03
TRINITY_DN1606_c0_g1_i3	-4,03	3,93E-03
TRINITY_DN1847_c0_g1_i10	-4,03	3,52E-03
TRINITY_DN1528_c0_g1_i7	-4,03	4,64E-03
TRINITY_DN2348_c0_g1_i2	-4,03	5,10E-03
TRINITY_DN190_c0_g1_i1	-4,02	3,75E-03
TRINITY_DN1712_c0_g1_i2	-4,02	3,87E-03
TRINITY_DN44_c0_g1_i6	-4,02	4,78E-03
TRINITY_DN2296_c0_g1_i3	-4,01	4,06E-03
TRINITY_DN7343_c0_g1_i1	-4,01	3,53E-03
TRINITY_DN989_c0_g1_i2	-4,01	4,12E-03
TRINITY_DN2186_c0_g1_i6	-4,00	5,08E-03
TRINITY_DN1287_c0_g1_i3	-4,00	3,51E-03
TRINITY_DN1403_c0_g1_i6	-4,00	5,85E-03
TRINITY_DN610_c0_g1_i25	-3,99	3,72E-03
TRINITY_DN62_c0_g1_i12	-3,99	4,33E-03
TRINITY_DN4029_c0_g1_i11	-3,99	6,60E-03
TRINITY_DN1376_c0_g1_i2	-3,99	4,94E-03
TRINITY_DN3603_c0_g1_i2	-3,99	3,63E-03
TRINITY_DN3461_c0_g1_i3	-3,99	4,18E-03
TRINITY_DN3440_c0_g1_i3	-3,98	6,92E-03
TRINITY_DN240_c1_g1_i2	-3,98	3,76E-03

Trinity ID	LogFC	FDR
TRINITY_DN323_c0_g1_i4	-3,98	3,85E-03
TRINITY_DN1452_c0_g1_i12	-3,98	4,17E-03
TRINITY_DN8508_c0_g1_i1	-3,98	6,90E-03
TRINITY_DN1961_c0_g1_i2	-3,98	5,23E-03
TRINITY_DN5686_c0_g1_i4	-3,97	3,80E-03
TRINITY_DN2816_c0_g1_i2	-3,97	4,53E-03
TRINITY_DN5332_c0_g1_i1	-3,96	3,85E-03
TRINITY_DN2082_c0_g1_i1	-3,96	4,11E-03
TRINITY_DN2409_c0_g1_i1	-3,96	5,27E-03
TRINITY_DN995_c0_g1_i8	-3,95	7,49E-03
TRINITY_DN1191_c0_g1_i7	-3,95	3,90E-03
TRINITY_DN2186_c0_g1_i8	-3,94	5,03E-03
TRINITY_DN437_c0_g1_i16	-3,94	4,39E-03
TRINITY_DN6050_c0_g1_i1	-3,94	4,16E-03
TRINITY_DN2356_c0_g1_i2	-3,94	4,09E-03
TRINITY_DN388_c0_g1_i13	-3,93	4,89E-03
TRINITY_DN1177_c0_g1_i9	-3,93	5,06E-03
TRINITY_DN1693_c0_g1_i1	-3,93	4,12E-03
TRINITY_DN2195_c0_g1_i6	-3,93	5,91E-03
TRINITY_DN1554_c0_g1_i1	-3,92	4,37E-03
TRINITY_DN1595_c0_g1_i3	-3,92	5,49E-03
TRINITY_DN1045_c0_g1_i4	-3,92	4,51E-03
TRINITY_DN214_c0_g1_i1	-3,92	4,41E-03
TRINITY_DN28_c1_g1_i39	-3,92	4,51E-03
TRINITY_DN1358_c0_g1_i13	-3,92	6,67E-03
TRINITY_DN1899_c0_g1_i1	-3,92	5,58E-03
TRINITY_DN3513_c0_g1_i1	-3,91	6,78E-03
TRINITY_DN2135_c0_g1_i3	-3,91	5,32E-03
TRINITY_DN1618_c0_g1_i9	-3,91	5,89E-03
TRINITY_DN7013_c0_g1_i1	-3,90	5,17E-03
TRINITY_DN4797_c0_g1_i2	-3,90	6,06E-03
TRINITY_DN282_c0_g1_i4	-3,90	4,48E-03
TRINITY_DN224_c1_g1_i13	-3,89	7,60E-03
TRINITY_DN754_c1_g2_i11	-3,89	5,38E-03
TRINITY_DN2330_c0_g1_i4	-3,89	8,34E-03
TRINITY_DN1449_c6_g1_i1	-3,89	6,94E-03
TRINITY_DN3756_c0_g1_i1	-3,89	6,71E-03
TRINITY_DN306_c1_g1_i1	-3,89	4,56E-03
TRINITY_DN546_c0_g1_i22	-3,89	4,61E-03
TRINITY_DN1826_c0_g1_i9	-3,88	4,79E-03
TRINITY_DN789_c0_g1_i24	-3,88	5,78E-03
TRINITY_DN312_c1_g1_i10	-3,88	5,39E-03

Trinity ID	LogFC	FDR
TRINITY_DN1247_c0_g1_i6	-3,88	4,66E-03
TRINITY_DN1228_c0_g1_i38	-3,87	7,81E-03
TRINITY_DN1057_c0_g1_i4	-3,87	5,53E-03
TRINITY_DN2647_c0_g1_i5	-3,87	5,04E-03
TRINITY_DN2320_c0_g1_i3	-3,87	4,90E-03
TRINITY_DN154_c0_g1_i8	-3,86	4,88E-03
TRINITY_DN239_c0_g2_i3	-3,86	5,43E-03
TRINITY_DN623_c1_g1_i1	-3,85	5,95E-03
TRINITY_DN97_c0_g3_i10	-3,85	6,24E-03
TRINITY_DN2151_c0_g1_i5	-3,85	6,95E-03
TRINITY_DN286_c0_g1_i4	-3,85	5,22E-03
TRINITY_DN164_c0_g1_i4	-3,85	5,04E-03
TRINITY_DN1693_c0_g1_i2	-3,85	5,61E-03
TRINITY_DN38_c0_g1_i5	-3,84	7,67E-03
TRINITY_DN164_c0_g1_i1	-3,84	5,11E-03
TRINITY_DN11940_c0_g1_i1	-3,84	5,31E-03
TRINITY_DN1489_c0_g1_i1	-3,84	5,14E-03
TRINITY_DN7922_c0_g1_i9	-3,83	5,30E-03
TRINITY_DN1487_c0_g1_i1	-3,83	6,02E-03
TRINITY_DN12644_c0_g1_i1	-3,83	7,12E-03
TRINITY_DN1415_c0_g1_i4	-3,82	6,08E-03
TRINITY_DN693_c0_g1_i2	-3,82	6,18E-03
TRINITY_DN13_c0_g1_i35	-3,82	6,94E-03
TRINITY_DN4450_c0_g1_i1	-3,82	5,78E-03
TRINITY_DN583_c0_g1_i6	-3,81	5,43E-03
TRINITY_DN486_c0_g1_i7	-3,81	6,49E-03
TRINITY_DN2347_c0_g2_i10	-3,81	7,35E-03
TRINITY_DN2542_c0_g1_i14	-3,81	5,75E-03
TRINITY_DN2375_c0_g1_i2	-3,81	5,87E-03
TRINITY_DN3135_c0_g1_i2	-3,81	5,78E-03
TRINITY_DN3071_c0_g1_i10	-3,80	7,47E-03
TRINITY_DN693_c0_g1_i9	-3,80	7,48E-03
TRINITY_DN10496_c0_g1_i1	-3,80	5,94E-03
TRINITY_DN4549_c0_g1_i2	-3,80	8,91E-03
TRINITY_DN1916_c0_g2_i8	-3,80	7,69E-03
TRINITY_DN710_c0_g1_i10	-3,78	6,50E-03
TRINITY_DN2032_c0_g1_i2	-3,78	5,87E-03
TRINITY_DN4855_c0_g1_i2	-3,78	5,98E-03
TRINITY_DN2816_c0_g1_i3	-3,78	5,88E-03
TRINITY_DN3528_c0_g1_i2	-3,78	5,95E-03
TRINITY_DN2173_c2_g1_i2	-3,77	7,74E-03
TRINITY_DN2236_c0_g1_i4	-3,77	7,39E-03

Trinity ID	LogFC	FDR
TRINITY_DN6521_c0_g1_i1	-3,77	6,10E-03
TRINITY_DN709_c0_g1_i1	-3,77	6,02E-03
TRINITY_DN4827_c0_g1_i1	-3,77	6,08E-03
TRINITY_DN13_c0_g1_i64	-3,76	6,33E-03
TRINITY_DN62_c0_g1_i9	-3,76	6,21E-03
TRINITY_DN2654_c0_g1_i13	-3,76	6,95E-03
TRINITY_DN2111_c0_g1_i4	-3,76	8,91E-03
TRINITY_DN1853_c0_g1_i1	-3,75	6,71E-03
TRINITY_DN693_c0_g1_i20	-3,75	6,44E-03
TRINITY_DN835_c0_g1_i8	-3,75	6,40E-03
TRINITY_DN40_c0_g1_i10	-3,75	6,42E-03
TRINITY_DN128_c0_g1_i4	-3,75	8,06E-03
TRINITY_DN4713_c0_g1_i1	-3,75	6,78E-03
TRINITY_DN1660_c0_g1_i3	-3,74	7,99E-03
TRINITY_DN3652_c0_g1_i2	-3,74	6,71E-03
TRINITY_DN1835_c0_g1_i1	-3,74	8,63E-03
TRINITY_DN2866_c0_g1_i1	-3,74	6,69E-03
TRINITY_DN5066_c0_g1_i1	-3,74	7,40E-03
TRINITY_DN227_c1_g1_i5	-3,74	8,03E-03
TRINITY_DN465_c0_g1_i4	-3,73	7,65E-03
TRINITY_DN611_c0_g1_i22	-3,73	6,80E-03
TRINITY_DN1429_c0_g1_i44	-3,73	9,26E-03
TRINITY_DN1402_c0_g1_i5	-3,73	6,73E-03
TRINITY_DN769_c0_g1_i2	-3,73	6,75E-03
TRINITY_DN1228_c0_g1_i34	-3,73	8,32E-03
TRINITY_DN2881_c0_g1_i2	-3,73	6,85E-03
TRINITY_DN437_c0_g1_i24	-3,72	7,16E-03
TRINITY_DN3301_c0_g1_i2	-3,72	7,48E-03
TRINITY_DN2399_c0_g1_i2	-3,72	7,30E-03
TRINITY_DN306_c1_g1_i8	-3,72	7,57E-03
TRINITY_DN11174_c0_g1_i1	-3,71	8,67E-03
TRINITY_DN6_c0_g3_i2	-3,71	6,94E-03
TRINITY_DN8620_c0_g1_i1	-3,71	9,08E-03
TRINITY_DN1761_c0_g2_i1	-3,71	7,66E-03
TRINITY_DN1045_c0_g1_i3	-3,71	7,22E-03
TRINITY_DN270_c0_g1_i14	-3,71	6,94E-03
TRINITY_DN1411_c0_g1_i1	-3,71	7,86E-03
TRINITY_DN470_c0_g1_i10	-3,71	9,75E-03
TRINITY_DN1969_c0_g2_i1	-3,71	7,91E-03
TRINITY_DN1440_c0_g1_i5	-3,71	8,98E-03
TRINITY_DN2910_c0_g1_i4	-3,70	8,74E-03
TRINITY_DN475_c1_g1_i3	-3,70	7,49E-03

Trinity ID	LogFC	FDR
TRINITY_DN1140_c0_g1_i14	-3,70	8,55E-03
TRINITY_DN1811_c0_g1_i2	-3,70	7,16E-03
TRINITY_DN4811_c0_g1_i1	-3,69	7,45E-03
TRINITY_DN1889_c1_g1_i2	-3,69	8,06E-03
TRINITY_DN1253_c0_g1_i5	-3,69	8,65E-03
TRINITY_DN217_c0_g1_i11	-3,68	7,74E-03
TRINITY_DN828_c0_g1_i10	-3,68	8,21E-03
TRINITY_DN3119_c0_g1_i1	-3,67	7,51E-03
TRINITY_DN5578_c0_g1_i2	-3,67	8,17E-03
TRINITY_DN775_c0_g1_i2	-3,67	7,65E-03
TRINITY_DN317_c0_g2_i1	-3,67	7,55E-03
TRINITY_DN312_c0_g1_i2	-3,67	8,32E-03
TRINITY_DN1196_c0_g1_i5	-3,67	8,03E-03
TRINITY_DN2712_c0_g1_i3	-3,66	9,05E-03
TRINITY_DN3660_c0_g1_i4	-3,66	7,89E-03
TRINITY_DN1419_c0_g1_i5	-3,66	8,33E-03
TRINITY_DN1517_c0_g1_i4	-3,66	8,03E-03
TRINITY_DN590_c0_g1_i6	-3,65	8,07E-03
TRINITY_DN119_c1_g1_i4	-3,64	8,27E-03
TRINITY_DN1441_c1_g1_i6	-3,64	8,36E-03
TRINITY_DN2891_c0_g1_i2	-3,64	9,58E-03
TRINITY_DN70_c0_g1_i3	-3,64	8,30E-03
TRINITY_DN1441_c3_g1_i2	-3,63	8,42E-03
TRINITY_DN5385_c0_g2_i1	-3,63	9,23E-03
TRINITY_DN1240_c0_g1_i2	-3,63	9,00E-03
TRINITY_DN3343_c0_g2_i1	-3,63	9,37E-03
TRINITY_DN1763_c0_g1_i4	-3,62	8,58E-03
TRINITY_DN2876_c0_g1_i2	-3,62	9,34E-03
TRINITY_DN973_c0_g2_i4	-3,62	9,21E-03
TRINITY_DN166_c0_g1_i5	-3,61	8,95E-03
TRINITY_DN286_c0_g1_i2	-3,61	8,76E-03
TRINITY_DN1238_c1_g1_i1	-3,61	9,44E-03
TRINITY_DN480_c0_g1_i4	-3,61	8,80E-03
TRINITY_DN2194_c0_g1_i1	-3,61	9,48E-03
TRINITY_DN3637_c0_g1_i2	-3,61	8,88E-03
TRINITY_DN877_c0_g1_i1	-3,59	8,95E-03
TRINITY_DN1777_c2_g1_i2	-3,59	9,06E-03
TRINITY_DN486_c0_g1_i3	-3,59	9,44E-03
TRINITY_DN13_c0_g1_i36	-3,59	9,39E-03
TRINITY_DN347_c1_g1_i1	-3,59	9,09E-03
TRINITY_DN764_c0_g1_i16	-3,59	9,44E-03
TRINITY_DN412_c0_g1_i2	-3,58	9,36E-03

Trinity ID	LogFC	FDR
TRINITY_DN1433_c0_g1_i1	-3,58	9,58E-03
TRINITY_DN2111_c0_g1_i1	-3,56	1,00E-02
TRINITY_DN1191_c0_g1_i3	-3,56	9,96E-03
TRINITY_DN4552_c0_g1_i1	-3,55	1,00E-02
TRINITY_DN1848_c0_g1_i1	-3,55	9,91E-03
