# Supplementary material for: Surviving in the Brine: A Multi-Omics Approach for Understanding the Physiology of the Halophile Fungus Aspergillus sydowii at Saturated NaCl Concentration
Source: Front Microbiol. 2022 May 2;13:840408. doi: 10.3389/fmicb.2022.840408 (PMC9108488; doi:10.3389/fmicb.2022.840408)
Supplement: Supplementary Table S3 — Transcriptomic expression of A. sydowii EXF-12860’s genes related to amino acid metabolism at 5.13 M NaCl compared to 1 M NaCl. [file Data_Sheet_3.PDF]

Supplementary Table S3. Transcriptomic expression of *Aspergillus sydowii* EXF-12860's genes related to amino acid metabolism at 5.13 M NaCl compared to 1 M NaCl.

Amino acid metabolism	Transcript ID	Description	Gene ID	LogFC	FDR
GLU	TRINITY_DN713_c0_g1_i7	glutamate dehydrogenase	*gdh2*	-11.72	6.97E-09
	TRINITY_DN713_c0_g1_i4			-7.42	1.21E-06
TYR/PHE	TRINITY_DN759_c0_g1_i6	3-deoxy-7-phosphoheptulonate synthase	*aroG*	-11.50	1.56E-08
	TRINITY_DN759_c0_g1_i15			-6.33	6.56E-06
HIS	TRINITY_DN380_c0_g1_i3	glucose-6-phosphate 1-dehydrogenase	*g6pd*	-11.22	2.99E-08
GLY	TRINITY_DN1487_c0_g1_i12	Cys-Gly metallodipeptidase	*dug1*	-9.92	7.31E-07
	TRINITY_DN1487_c0_g1_i5			-9.36	2.89E-06
	TRINITY_DN1487_c0_g1_i2			-4.86	4.85E-04
	TRINITY_DN1487_c0_g1_i1			-3.83	6.02E-03
HIS	TRINITY_DN182_c0_g1_i6	triosephosphate isomerase	*tpi1*	-9.92	2.89E-07
CYS	TRINITY_DN573_c0_g1_i9	thiosulfate/3-mercaptopyruvate sulfurtransferase	*tst*	-9.57	1.71E-06
MET	TRINITY_DN95_c0_g1_i26	methylthioribose-1-phosphate isomerase	*mtnA*	-9.34	3.10E-06
	TRINITY_DN95_c0_g1_i11			-8.95	8.67E-06
	TRINITY_DN95_c0_g1_i7			-8.88	1.04E-05
	TRINITY_DN95_c0_g1_i1			-8.59	2.35E-05
	TRINITY_DN95_c0_g1_i9			-4.71	6.22E-04
	TRINITY_DN95_c0_g1_i6			-4.10	3.88E-03
	TRINITY_DN95_c0_g1_i32			9.82	8.42E-07
HIS	TRINITY_DN9078_c0_g1_i1	glyceraldehyde-3-phosphate dehydrogenase	*gpdA*	-9.26	3.74E-06
TYR/PHE	TRINITY_DN1765_c0_g1_i3	3-dehydroquinate dehydratase	*aroD*	-9.10	5.81E-06
VAL/LEU/ILE	TRINITY_DN5050_c0_g1_i2	dihydroxy-acid dehydratase	*ilvD*	-8.97	8.19E-06
CYS	TRINITY_DN1285_c0_g1_i27	cysteine synthase	*cysK*	-8.34	2.89E-06
	TRINITY_DN1285_c0_g1_i15			6.75	3.82E-06
	TRINITY_DN1285_c0_g1_i25	cystathionine beta-synthase	*cbs*	-7.43	4.95E-05
	TRINITY_DN1285_c0_g1_i43			4.06	4.16E-03
HIS	TRINITY_DN12621_c0_g1_i1	6-phosphogluconate dehydrogenase	*gnd*	-6.99	1.63E-06
SER	TRINITY_DN242_c0_g1_i39	D-3-phosphoglycerate dehydrogenase	*3-pgdh*	-6.95	1.48E-06
	TRINITY_DN242_c0_g1_i28			-6.76	3.20E-06
	TRINITY_DN242_c0_g1_i48			7.49	9.10E-07
SER	TRINITY_DN28_c1_g1_i14	phosphoglycerate kinase	*pgk1*	-5.10	2.41E-04
ILE	TRINITY_DN800_c0_g1_i6	acetolactate synthase	*ilvH*	-5.05	7.27E-04
	TRINITY_DN800_c0_g1_i1			-4.13	3.87E-03
HIS	TRINITY_DN4830_c0_g2_i1	hercynylcysteine S-oxide synthase	*egt1*	-4.72	8.88E-04
PRO	TRINITY_DN217_c0_g1_i16	4-hydroxy-2-oxoglutarate aldolase	*hoga1*	-4.69	6.22E-04
	TRINITY_DN217_c0_g1_i26			-4.56	1.46E-03
	TRINITY_DN217_c0_g1_i11			-3.68	7.74E-03

Amino acid metabolism	Transcript ID	Description	Gene ID	LogFC	FDR
PRO	TRINITY_DN217_c0_g1_i14	4-hydroxy-2-oxoglutarate aldolase	*hoga1*	5.32	2.31E-04
	TRINITY_DN217_c0_g1_i35			5.34	2.17E-04
	TRINITY_DN217_c0_g1_i22			6.42	1.36E-05
	TRINITY_DN217_c0_g1_i18			6.57	2.52E-05
	TRINITY_DN217_c0_g1_i27			6.89	2.29E-06
	TRINITY_DN217_c0_g1_i32			6.96	3.22E-05
	TRINITY_DN217_c0_g1_i21			7.08	9.35E-06
	TRINITY_DN217_c0_g1_i23			9.56	2.38E-08
	TRINITY_DN217_c0_g1_i20			10.43	3.84E-08
	TRINITY_DN217_c0_g1_i5			10.73	6.49E-08
THR	TRINITY_DN38_c0_g1_i5	O-acetylhomoserine/O-acetylserine sulfhydrylase	*met17*	-3.84	7.67E-03
HIS	TRINITY_DN11174_c0_g1_i1	glutamine synthetase	*glnA*	-3.71	8.67E-03
	TRINITY_DN775_c0_g1_i2	probable transketolase	*tktA*	-3.67	7.65E-03
MET	TRINITY_DN775_c0_g1_i2	spermidine synthase	*speE*	-3.67	7.65E-03
GLY	TRINITY_DN2342_c0_g1_i3	glutathionase	*ggt*	3.66	8.26E-03
	TRINITY_DN2342_c0_g1_i10			3.82	7.35E-03
	TRINITY_DN2402_c0_g1_i23			4.53	1.09E-03
	TRINITY_DN2342_c0_g1_i7			5.05	3.14E-04
CYS	TRINITY_DN2996_c0_g1_i1	homoserine O-acetyltransferase	*metX*	3.69	8.91E-03
		homoserine O-succinyltransferase	*metA*	3.69	8.91E-03
GLU	TRINITY_DN9836_c0_g1_i1	5-oxoprolinase	*oplah*	3.77	6.02E-03
HIS	TRINITY_DN745_c2_g1_i33	glutaminase	*hisF/H*	3.98	5.23E-03
	TRINITY_DN2038_c0_g1_i3	amidophosphoribosyltransferase	*purF*	4.19	2.73E-03
GLY	TRINITY_DN681_c0_g1_i14	peroxiredoxin	*prx1*	4.73	7.03E-04
MET	TRINITY_DN1888_c0_g2_i3	homocysteine S-methyltransferase	*mmuM*	4.75	1.12E-03
TRP	TRINITY_DN1700_c0_g1_i5	anthranilate synthase	*trpG*	5.63	1.83E-04
HIS	TRINITY_DN345_c0_g1_i9	6-phosphogluconolactonase	*pgls*	6.23	2.52E-05
	TRINITY_DN345_c0_g1_i5			7.28	4.84E-06
	TRINITY_DN345_c0_g1_i1			7.52	6.93E-07
	TRINITY_DN345_c0_g1_i2			8.52	1.23E-07
CYS	TRINITY_DN1197_c0_g1_i7	cystathionine gamma-lyase	*cth*	7.20	3.63E-06
VAL/LEU/ILE	TRINITY_DN5216_c0_g2_i1	branched-chain-amino-acid aminotransferase	*ilvE*	12.83	4.55E-10
