# Supplementary material for: Surviving in the Brine: A Multi-Omics Approach for Understanding the Physiology of the Halophile Fungus Aspergillus sydowii at Saturated NaCl Concentration
Source: Front Microbiol. 2022 May 2;13:840408. doi: 10.3389/fmicb.2022.840408 (PMC9108488; doi:10.3389/fmicb.2022.840408)
Supplement: Supplementary Table S4 — Transcriptomic expression of A. sydowii EXF-12860’s genes related to fatty acids metabolism at 5.13 M NaCl compared to 1 M NaCl. [file Data_Sheet_4.PDF]

Supplementary Table S4. Transcriptomic expression of *Aspergillus sydowii* EXF-12860's genes related to fatty acids metabolism at 5.13 M NaCl compared to 1 M NaCl.

Process	Transcript ID	Description	Gene ID	LogFC	FDR
Glycerophospholipid metabolism	TRINITY_DN560_c0_g1_i3	phosphatidylserine decarboxylase	*psd*	-11.42	1.88E-08
	TRINITY_DN2907_c0_g1_i2			-10.11	4.43E-07
	TRINITY_DN560_c0_g1_i5			-5.70	5.87E-05
	TRINITY_DN560_c0_g1_i12			-4.74	5.38E-04
	TRINITY_DN2907_c0_g1_i1			-4.70	6.35E-04
	TRINITY_DN560_c0_g1_i1			-4.58	3.02E-03
	TRINITY_DN560_c0_g1_i13			-4.39	1.43E-03
	TRINITY_DN1717_c0_g1_i1	phospholipase D1/2	*pld1_2*	-11.30	2.38E-08
	TRINITY_DN1717_c0_g1_i19			-11.19	3.24E-08
	TRINITY_DN1717_c0_g1_i25			-10.76	4.45E-08
	TRINITY_DN1717_c0_g1_i27			-10.16	3.93E-07
	TRINITY_DN1717_c0_g1_i6			-7.61	2.91E-07
	TRINITY_DN1717_c0_g1_i5			-5.71	3.63E-05
	TRINITY_DN1717_c0_g1_i10			-5.48	7.45E-05
	TRINITY_DN2866_c0_g1_i1			-3.74	6.69E-03
	TRINITY_DN2706_c0_g1_i4	phosphatidylglycerophosphatase	*gep4*	-8.70	2.34E-08
	TRINITY_DN640_c0_g1_i2	glycerol-3-phosphate dehydrogenase [NAD+]	*gpd1*	-8.10	3.16E-06
	TRINITY_DN640_c0_g1_i15			-7.11	1.10E-05
	TRINITY_DN640_c0_g1_i9			-4.25	2.01E-03
	TRINITY_DN640_c0_g1_i17			-6.61	6.15E-06
	TRINITY_DN534_c0_g1_i20			-5.93	2.04E-05
	TRINITY_DN534_c0_g1_i6			-5.75	3.54E-05
	TRINITY_DN640_c0_g1_i3			-5.48	7.04E-05
	TRINITY_DN534_c0_g1_i7			-5.19	1.71E-04
	TRINITY_DN640_c0_g1_i1			-5.14	1.83E-04
	TRINITY_DN640_c0_g1_i7			-4.82	9.59E-04
	TRINITY_DN640_c0_g1_i14			-4.13	3.75E-03
	TRINITY_DN640_c0_g1_i8			-4.05	3.17E-03
	TRINITY_DN149_c0_g1_i11	glycerol-3-phosphate dehydrogenase	*glpA*	-4.64	1.09E-03
	TRINITY_DN774_c0_g1_i6	CDP-diacylglycerol--serine O-phosphatidyltransferase	*pssA*	-4.11	5.12E-03
	TRINITY_DN5377_c1_g1_i3	CDP-diacylglycerol--inositol 3-phosphatidyltransferase	*cdipt*	3.82	6.10E-03
	TRINITY_DN5377_c1_g1_i2			4.26	2.33E-03
Glycerolipid metabolism	TRINITY_DN194_c0_g1_i1	alcohol dehydrogenase (NADP+)	*adh*	-10.24	4.89E-09
	TRINITY_DN194_c0_g1_i2			-7.46	3.79E-07
	TRINITY_DN4195_c0_g2_i1			6.11	4.61E-05
	TRINITY_DN590_c0_g1_i9	diacylglycerol diphosphate phosphatase	*dpp1*	-4.68	6.37E-04
	TRINITY_DN1861_c0_g1_i4	glycerol kinase	*glpK*	-4.30	2.41E-03
	TRINITY_DN1262_c0_g1_i5	acylglycerol lipase	*mgll*	3.88	5.40E-03

Process	Transcript ID	Description	Gene ID	LogFC	FDR
Fatty acid biosynthesis	TRINITY_DN1784_c0_g1_i4	fatty acid synthase beta subunit	*fas1*	-7.04	2.03E-06
	TRINITY_DN1405_c0_g1_i1			-6.80	5.07E-06
	TRINITY_DN1405_c0_g1_i2	fatty acid synthase alpha subunit	*fas2*	-4.90	3.50E-04
	TRINITY_DN1405_c0_g1_i6			-4.04	3.89E-03
	TRINITY_DN231_c0_g2_i9	long chain fatty acyl-CoA synthetase	*fadD*	-4.31	4.22E-03
	TRINITY_DN12104_c0_g1_i1	3-oxoacyl-[acyl-carrier-protein] reductase	*fabG*	4.26	1.90E-03
Fatty acid elongation	TRINITY_DN1007_c0_g1_i8	very-long-chain (3R)-3-hydroxyacyl-CoA dehydratase	*hacd*	-10.14	4.14E-07
	TRINITY_DN1007_c0_g1_i10			-9.92	7.37E-07
	TRINITY_DN1007_c0_g1_i21			-5.89	2.23E-04
	TRINITY_DN1007_c0_g1_i19			9.73	1.19E-06
	TRINITY_DN1007_c0_g1_i3			10.53	1.64E-07
Synthesis and degradation of ketone bodies	TRINITY_DN1899_c0_g1_i6	3-oxoacid CoA-transferas	*oxct*	-9.57	1.71E-06
	TRINITY_DN1899_c0_g1_i5			-9.54	1.81E-06
	TRINITY_DN1899_c0_g1_i2			-5.14	2.47E-04
	TRINITY_DN1899_c0_g1_i1			-3.92	5.58E-03
	TRINITY_DN1899_c0_g1_i4			-4.23	2.02E-03
Steroid biosynthesis	TRINITY_DN1548_c0_g1_i10	sterol esterase	*lipa*	-4.37	1.53E-03
	TRINITY_DN1548_c0_g1_i5			-4.04	4.45E-03
	TRINITY_DN3521_c0_g1_i1	lanosterol synthase	*lss*	-4.37	1.89E-03
	TRINITY_DN509_c0_g1_i3	sterol O-acyltransferase	*soat*	-4.33	1.74E-03
	TRINITY_DN509_c0_g1_i11			-4.20	4.86E-03
