# Supplementary material for: Surviving in the Brine: A Multi-Omics Approach for Understanding the Physiology of the Halophile Fungus Aspergillus sydowii at Saturated NaCl Concentration
Source: Front Microbiol. 2022 May 2;13:840408. doi: 10.3389/fmicb.2022.840408 (PMC9108488; doi:10.3389/fmicb.2022.840408)
Supplement: Supplementary Table S5 — Transcriptomic expression of A. sydowii EXF-12860’s genes related to cell cycle, high glycerol pathway (HOG), compatible solutes, cell wall, pheromone synthesis, cellular membrane transporters, calcium pathway, and morphology at 5.13 M NaCl compared to 1 M NaCl. [file Data_Sheet_5.PDF]

Supplementary Table S5. Transcriptomic expression of *Aspergillus sydowii* EXF-12860's genes related to cell cycle, high glycerol pathway (HOG), compatible solutes, cell wall, pheromone synthesis, cellular membrane transporters, calcium pathway, and morphology at 5.13 M NaCl compared to 1 M NaCl.

Process	Trinity ID	Description	Gene ID	LogFC	FDR
Cell cycle	TRINITY_DN511_c0_g1_i19	cullin 1	*cdc53*	-11,31	2,38E-08
	TRINITY_DN511_c0_g1_i25			-10,35	2,57E-07
	TRINITY_DN511_c0_g1_i34			-10,21	3,45E-07
	TRINITY_DN511_c0_g1_i27			-9,54	1,86E-06
	TRINITY_DN3603_c0_g1_i1	G2/mitotic-specific cyclin 3/4	*clb3_4*	-10,54	1,62E-07
	TRINITY_DN3603_c0_g1_i2			-3,99	3,63E-03
	TRINITY_DN1440_c0_g1_i3	serine/threonine-protein kinase	*chek2*	-10,22	3,42E-07
	TRINITY_DN1440_c0_g1_i5			-3,71	8,98E-03
	TRINITY_DN4013_c0_g1_i2	structural maintenance of chromosome 2	*smc2*	-9,77	1,09E-06
	TRINITY_DN4013_c0_g1_i3			-8,97	8,19E-06
	TRINITY_DN4013_c0_g1_i1			-4,55	8,99E-04
	TRINITY_DN1956_c0_g1_i4	mitotic spindle assembly checkpoint protein	*mad1*	-9,62	1,50E-06
	TRINITY_DN2580_c0_g1_i7	mitosis inhibitor protein kinase	*swe1*	-9,32	3,18E-06
	TRINITY_DN2580_c0_g1_i4			-5,13	2,28E-04
	TRINITY_DN4414_c0_g1_i2	anaphase-promoting complex subunit 3	*apc3*	-9,07	6,15E-06
	TRINITY_DN4637_c0_g1_i3	separase	*esp1*	-6,37	8,79E-06
	TRINITY_DN385_c0_g1_i5	anaphase-promoting complex subunit 5	*apc5*	-6,23	1,64E-05
	TRINITY_DN385_c0_g1_i8			-5,38	2,88E-04
	TRINITY_DN945_c0_g1_i11	serine/threonine-protein phosphatase 2A regulatory subunit B	*cdc55*	-5,89	1,40E-04
	TRINITY_DN945_c0_g1_i12			-5,59	1,56E-04
	TRINITY_DN945_c0_g1_i5			-4,75	1,00E-03
	TRINITY_DN1000_c0_g1_i4	cyclin-dependent kinase regulatory subunit	*cks1*	-5,45	2,94E-04
	TRINITY_DN2023_c0_g1_i2	checkpoint serine/threonine-protein kinase	*bub1*	-5,21	2,01E-04
	TRINITY_DN240_c1_g1_i1	structural maintenance of chromosome 4	*smc4*	-4,55	9,07E-04
	TRINITY_DN240_c1_g1_i2			-3,98	3,76E-03
	TRINITY_DN986_c0_g1_i7	condensin complex subunit 1	*ycs4*	-4,81	6,17E-04
	TRINITY_DN1240_c0_g1_i1	G2/mitotic-specific cyclin 2	*clb2*	-4,43	1,45E-03
	TRINITY_DN1240_c0_g1_i2			-3,63	9,00E-03
	TRINITY_DN767_c0_g1_i6	M-phase inducer tyrosine phosphatase	*mih1*	-4,36	1,45E-03
	TRINITY_DN767_c0_g1_i3			-4,18	2,39E-03
	TRINITY_DN2184_c0_g1_i1	F-box and WD-40 domain protein	*cdc4*	-4,32	1,78E-03
	TRINITY_DN5435_c0_g1_i1	F-box and leucine-rich repeat protein	*grr1*	-4,27	3,12E-03
	TRINITY_DN603_c0_g1_i8	condensin complex subunit 2	*brn1*	5,57	5,88E-05
	TRINITY_DN603_c0_g1_i12			6,37	2,97E-05

Process	Trinity ID	Description	Gene ID	LogFC	FDR
HOG	TRINITY_DN2041_c0_g1_i3	Serine/threonine-protein kinase	*ste20*	-10,28	3,05E-07
	TRINITY_DN511_c0_g2_i3	mitogen-activated protein kinase hog1	*hog1*	-8,70	1,71E-05
	TRINITY_DN1709_c0_g1_i10			-6,21	5,05E-05
	TRINITY_DN1709_c0_g1_i3			-4,04	3,38E-03
	TRINITY_DN2375_c0_g1_i2	mitogen-activated protein kinase kinase kinase	*ssk2*	-3,81	5,87E-03
	TRINITY_DN702_c1_g1_i8	osomolarity two-component system	*ssk1*	11,80	6,97E-09
	TRINITY_DN702_c1_g1_i9			11,49	1,59E-08
	TRINITY_DN702_c1_g1_i8	high osmolarity signaling protein sho1	*sho1*	11,80	6,97E-09
	TRINITY_DN702_c1_g1_i9			11,49	1,59E-08
	TRINITY_DN8213_c1_g1_i3	serine/threonine-protein kinase	*cla4*	4,15	2,42E-03
Compatible solutes	TRINITY_DN640_c0_g1_i1	glycerol-3-phosphate dehydrogenase (NAD+)	*gpd1*	-8,10	3,16E-06
	TRINITY_DN640_c0_g1_i15			-7,11	1,10E-05
	TRINITY_DN640_c0_g1_i14			-6,61	6,15E-06
	TRINITY_DN534_c0_g1_i20			-5,93	2,04E-05
	TRINITY_DN534_c0_g1_i6			-5,75	3,54E-05
	TRINITY_DN640_c0_g1_i17			-5,48	7,04E-05
	TRINITY_DN534_c0_g1_i7			-5,19	1,71E-04
	TRINITY_DN640_c0_g1_i2			-5,14	1,83E-04
	TRINITY_DN640_c0_g1_i3			-4,82	9,59E-04
	TRINITY_DN640_c0_g1_i7			-4,25	2,01E-03
	TRINITY_DN640_c0_g1_i9			-4,13	3,75E-03
	TRINITY_DN640_c0_g1_i8			-4,05	3,17E-03
	TRINITY_DN892_c0_g1_i10	catalase A	*katE*	-14,33	1,37E-11
	TRINITY_DN892_c0_g1_i9			-11,94	2,53E-09
	TRINITY_DN892_c0_g1_i11			-11,27	2,46E-08
	TRINITY_DN892_c0_g1_i6			-5,08	2,69E-04
	TRINITY_DN892_c0_g1_i3			-4,12	2,57E-03
Pheromone synthesis	TRINITY_DN1776_c3_g1_i2	bud emergence protein 1	*bem1*	-5,53	8,12E-05
	TRINITY_DN530_c0_g1_i39	E3 ubiquitin-protein ligase	*rsp5*	4,19	2,73E-03

Process	Trinity ID	Description	Gene ID	LogFC	FDR
Transporters	TRINITY_DN2138_c0_g1_i1	solute carrier family 2 (myo-inositol transporter)	*itr*	-8,94	3,03E-05
	TRINITY_DN3980_c0_g1_i1	hexose carrier protein	*hex6*	-8,40	3,82E-05
	TRINITY_DN39_c0_g1_i10	low-affinity potassium transport protein	*trk2*	-5,37	1,18E-04
	TRINITY_DN39_c0_g1_i14			-4,76	6,86E-04
	TRINITY_DN565_c1_g2_i10	copper transporter	*ctr*	3,61	8,84E-03
	TRINITY_DN565_c1_g2_i2			7,04	1,50E-06
	TRINITY_DN254_c0_g1_i5	amino acid permease-domain-containing protein	*aap*	4,03	3,51E-03
	TRINITY_DN254_c0_g1_i27			4,08	3,61E-03
	TRINITY_DN254_c0_g1_i25			4,83	5,98E-04
	TRINITY_DN3563_c0_g2_i7	putative amino acid permease		4,32	2,43E-03
	TRINITY_DN2714_c0_g1_i2			4,49	2,66E-03
	TRINITY_DN2714_c0_g1_i1			5,13	3,87E-04
	TRINITY_DN1077_c0_g1_i5			9,37	2,76E-06
	TRINITY_DN3908_c0_g1_i3	sugar:H+ symporter	*hxt*	4,04	4,12E-03
	TRINITY_DN4861_c0_g2_i5			4,39	2,22E-03
	TRINITY_DN4861_c0_g2_i2			4,62	8,65E-04
	TRINITY_DN151_c0_g1_i16	putative sugar transporter	*stl1*	4,57	1,29E-03
	TRINITY_DN151_c0_g1_i17			4,63	2,50E-03
	TRINITY_DN991_c0_g1_i3			5,08	3,18E-04
	TRINITY_DN264_c0_g1_i24	sodium transport ATPase 5	*ena5*	4,69	7,12E-04
	TRINITY_DN264_c0_g1_i26			4,99	3,26E-04
	TRINITY_DN264_c0_g1_i8			5,68	1,02E-04
	TRINITY_DN264_c0_g1_i22			6,93	3,37E-06
	TRINITY_DN62_c0_g1_i17	P-type cation-transporting ATPase	*pca1*	6,79	1,54E-05
Calcium pathway	TRINITY_DN759_c0_g1_i6	calcium permeable stress-gated cation channel 1	*csc1*	-11,50	1,56E-08
	TRINITY_DN759_c0_g1_i15			-6,33	6,56E-06
	TRINITY_DN4050_c0_g1_i15	calcium/calmodulin-dependent protein kinase kinase 2	*camk2*	-11,36	2,16E-08
	TRINITY_DN4050_c0_g1_i13			4,42	1,53E-03
	TRINITY_DN3533_c0_g1_i3	P-type Ca2+ transporter type 2C	*atp2c*	-8,18	7,03E-05
	TRINITY_DN3533_c0_g1_i5			-4,05	7,40E-03
	TRINITY_DN946_c1_g1_i1	calcium uniporter protein, mitochondrial	*mcu*	-4,41	1,28E-03
	TRINITY_DN2758_c0_g1_i4	Ca2+:H+ antiporter	*chaA*	-4,40	1,42E-03
	TRINITY_DN791_c0_g1_i1	calmodulin	*calm*	4,62	1,39E-03

Process	Trinity ID	Description	Gene ID	LogFC	FDR
Morphology	TRINITY_DN1136_c0_g1_i3	polarized growth protein	*boi2*	-11,57	4,88E-09
	TRINITY_DN454_c0_g1_i17	Rho GTPase-activating protein	*rgd1*	-11,13	3,84E-08
	TRINITY_DN2560_c0_g1_i4	myosin V	*myo5*	-9,71	1,93E-08
	TRINITY_DN3781_c0_g1_i2	dynein light intermediate chain 1, cytosolic	*dync1li*	-8,89	1,01E-05
	TRINITY_DN4474_c0_g1_i1	dynein heavy chain 1, cytosolic	*dync1h*	-4,63	3,08E-03
	TRINITY_DN4474_c0_g2_i1			-4,05	9,83E-04
	TRINITY_DN167_c1_g1_i6	actin cortical patch SUR7	*sur7*	-7,8	7,51E-07
	TRINITY_DN167_c1_g1_i30			-4,36	3,59E-03
	TRINITY_DN167_c1_g1_i32			-4,32	2,21E-03
	TRINITY_DN1837_c0_g1_i2	abnormal spindle-like microcephaly-associated protein	*asp*	-6,14	4,38E-07
	TRINITY_DN1776_c3_g1_i2	bud emergence protein 1	*bem1*	-5,53	2,91E-06
	TRINITY_DN5375_c0_g1_i1	myosin heavy chain	*myh9s*	-5,19	6,16E-06
	TRINITY_DN4731_c0_g1_i1	Ras GTPase-activating-like protein IQGAP2/3	*iqgap*	-4,84	1,81E-05
	TRINITY_DN1240_c0_g1_i1	G2/mitotic-specific cyclin 2	*clb2*	-4,43	1,45E-03
	TRINITY_DN1240_c0_g1_i2			-3,63	9,00E-03
	TRINITY_DN368_c1_g1_i2	Ras-related GTP-binding protein C/D	*rragc*	-4,39	8,17E-05
	TRINITY_DN150_c0_g2_i4	actin related protein 2/3 complex, subunit 2	*arpc2*	-4,15	2,96E-04
	TRINITY_DN7343_c0_g1_i1	dynactin 1	*dctn1*	-4,05	3,53E-03
	TRINITY_DN5196_c0_g1_i1	protein NUD1	*nud1*	-4,05	2,07E-04
	TRINITY_DN3603_c0_g1_i2	G2/mitotic-specific cyclin 3/4	*clb3_4*	-3,99	3,63E-03
	TRINITY_DN593_c0_g2_i7	actin-related protein 5	*arp5*	4,54	1,27E-03
Cell wall	TRINITY_DN590_c0_g1_i4	GPI anchored cell wall protein, putative	*gpi*	-13,93	5,54E-13
	TRINITY_DN1767_c1_g1_i10	mitogen-activated protein kinase kinase	*mkk1_2*	-13,44	7,96E-11
	TRINITY_DN1767_c1_g1_i6			-8,70	1,64E-05
	TRINITY_DN1767_c1_g1_i11			-8,55	2,53E-05
	TRINITY_DN470_c1_g2_i6	mitogen-activated protein kinase kinase kinase	*bck1*	-10,53	1,67E-07
	TRINITY_DN470_c1_g2_i3			-9,70	1,24E-06
	TRINITY_DN470_c1_g2_i5			-9,52	1,96E-06
	TRINITY_DN2789_c0_g1_i2	chitin synthase D	*chsD*	-10,30	2,97E-07
	TRINITY_DN863_c1_g1_i3	cell wall biogenesis protein phosphatase	*ssd1*	-10,16	3,90E-07
	TRINITY_DN2159_c0_g1_i2	mannose-6-phosphate isomerase	*mpi*	-9,67	1,31E-06
	TRINITY_DN2159_c0_g1_i1			-5,25	1,73E-04
	TRINITY_DN359_c0_g1_i4	glycolipid 2-alpha-mannosyltransferase-domain-containing protein	*mnt1*	-9,43	2,34E-06
	TRINITY_DN2867_c0_g1_i2	phosphatidylinositol 4-kinase	*stt4*	-9,37	2,76E-06
	TRINITY_DN1393_c0_g1_i8	putative chitin synthase activator	*chs3*	-9,01	3,45E-07

Process	Trinity ID	Description	Gene ID	LogFC	FDR
Cell wall	TRINITY_DN164_c0_g1_i2	mannosyltransferase putative-domain-containing protein	*gmpp*	-6,87	2,33E-06
	TRINITY_DN164_c0_g1_i3			-4,78	5,11E-04
	TRINITY_DN164_c0_g1_i4			-3,85	5,04E-03
	TRINITY_DN164_c0_g1_i1			-3,84	5,11E-03
	TRINITY_DN1113_c0_g1_i2	cytokinesis protein	*bni1*	-5,32	1,73E-04
	TRINITY_DN12654_c0_g1_i1	endochitinase A	*chiA*	-4,86	4,06E-04
	TRINITY_DN1016_c0_g1_i4	mannose-1-phosphate guanyltransferase	*psa1*	-4,63	1,11E-03
	TRINITY_DN2146_c0_g1_i2	1,3-beta-glucan synthase component	*fks2*	-4,32	1,61E-03
	TRINITY_DN2196_c0_g1_i3	cell wall biogenesis protein	*mhp1*	-4,27	1,83E-03
	TRINITY_DN2816_c0_g1_i2	rho1 guanine nucleotide exchange factor 1	*rgf1*	-3,97	4,53E-03
	TRINITY_DN2816_c0_g1_i3			-3,78	5,88E-03
	TRINITY_DN2816_c0_g1_i2	guanine nucleotide exchange factor 1	*rom1,2*	-3,97	4,53E-03
	TRINITY_DN764_c0_g1_i16	chitin synthase regulatory factor 3	*chr3*	-3,59	9,44E-03
	TRINITY_DN93_c0_g1_i37	glucanosyltransferase-domain-containing protein	*gas1*	3,81	8,10E-03
	TRINITY_DN93_c0_g1_i55			3,92	4,22E-03
	TRINITY_DN93_c0_g1_i65			3,94	4,38E-03
	TRINITY_DN93_c0_g1_i47			3,98	4,99E-03
	TRINITY_DN93_c0_g1_i33			4,14	2,70E-03
	TRINITY_DN93_c0_g1_i59			5,42	2,38E-04
	TRINITY_DN93_c0_g1_i58			5,72	4,33E-05
	TRINITY_DN93_c0_g1_i27			6,04	7,05E-05
	TRINITY_DN93_c0_g1_i39			6,73	8,33E-06
	TRINITY_DN93_c0_g1_i12			9,62	1,48E-06
	TRINITY_DN1685_c0_g1_i3	GTPase-activating protein	*sac7*	3,73	7,69E-03
	TRINITY_DN2146_c0_g1_i2	1,3-beta-glucan synthase component	*fks1*	4,33	1,61E-03
	TRINITY_DN4861_c0_g2_i5	probable mannan endo-1,4-beta-mannosidase A	*man1*	4,39	2,22E-03
